# Selective serotonin reuptake inhibitors and immune checkpoint blockade in solid tumors: A target trial emulation with pan-cancer transcriptomic analyses

**DOI:** 10.1371/journal.pmed.1005262

**Published:** 2026-09-29

**Authors:** Po-Huang Chen, Ming-Shen Dai, Mao-Hsuan Huang, Hsin-Yu Chen, Li-Ting Kao, Tina Yi-Jin Hsieh, Hong-Jie Jhou, Cho-Hao Lee

**Affiliations:** 1 Division of Hematology and Oncology, Department of Internal Medicine, Tri-Service General Hospital, National Defense Medical University, Taipei, Taiwan; 2 Department of Oncology, Tri-Service General Hospital, National Defense Medical University, Taipei, Taiwan; 3 Department of Psychiatry, Cheng Hsin General Hospital, Taipei, Taiwan; 4 Department of Family Medicine, Chi Mei Medical Center, Tainan, Taiwan; 5 Graduate Institute of Life Sciences, National Defense Medical University, Taipei, Taiwan; 6 School of Pharmacy, National Defense Medical University, Taipei, Taiwan; 7 Department of Pharmacy Practice, Tri-Service General Hospital, Taipei, Taiwan; 8 Department of Obstetrics and Gynecology, Beth Israel Deaconess Medical Center, Boston, Massachusetts, United States of America; 9 Neurological Institute, Changhua Christian Hospital, Changhua, Taiwan; University of the Ryukyus: Ryukyu Daigaku, JAPAN

## Abstract

**Background:**

Preclinical evidence identifies the serotonin transporter (SERT, encoded by SLC6A4) as an immune checkpoint on CD8+ T cells, with selective serotonin reuptake inhibitors (SSRIs) enhancing antitumor immunity in synergy with anti-programmed cell death protein 1 (anti-PD-1) therapy. Whether this translates into clinical benefit for patients receiving immune checkpoint inhibitors (ICIs) is unknown. Using a target trial emulation framework, we compared outcomes between SSRI users and benzodiazepine (BZD) users among patients with a solid tumor and depression or anxiety who were receiving an ICI. Separately, in The Cancer Genome Atlas (TCGA), which covers a different set of patients, we examined how SLC6A4 expression related to tumor immune-cell infiltration across cancers.

**Methods and findings:**

We emulated a target trial in the TriNetX federated electronic health record network (over 187 million patients), comparing SSRI with BZD treatment at first ICI administration under an intention-to-treat-analogue estimand. Eligible patients were adults with a solid tumor, a first ICI administration between 2015 and 2025, and a depressive or anxiety disorder recorded within the prior 365 days. Exposure required an active SSRI or BZD prescription overlapping the index date or starting within the preceding 90 days, not necessarily on that date. The primary outcome was 2-year overall survival; 20 secondary outcomes across 4 mechanistic pathways were prespecified. Propensity score matching (1:1) on 49 baseline covariates yielded 1,567 pairs. Estimates are hazard ratios (HRs) with 95% confidence intervals (CIs). Transcriptomic analyses used TCGA (8,272 tumor samples from 8,172 patients, 20 tumor types). Among 3,134 matched patients, SSRI use was associated with lower 2-year all-cause mortality (23.5% versus 34.4%; HR 0.627; 95% CI [0.549, 0.716]; *p* < 0.001; *E*-value 2.57). Composite immune-related adverse events (irAEs) were more frequent with SSRI use (HR 1.162; 95% CI [1.039, 1.299]; *p* = 0.008), driven by thyroid dysfunction (HR 1.199; 95% CI [1.053, 1.366]; *p* = 0.006), without increases in hepatitis, pneumonitis, or colitis (all *p* > 0.09). Newly coded distant metastases were less frequent in the SSRI group (HR 0.629; 95% CI [0.487, 0.811]; *p* < 0.001), and the 3 prespecified negative controls (nephrolithiasis, cholelithiasis, cataract) showed null associations (all *p* > 0.76). All 5 individually analyzable SSRIs were associated with lower mortality (HR range 0.61 to 0.71). In TCGA, SLC6A4 expression was inversely correlated with T-cell inflammation in 13 of 20 cancer types, and this organ-level pattern matched that of organ-specific irAEs. The main limitation is residual confounding: performance status, a clinician’s rating of everyday functional ability that strongly predicts cancer survival, is not recorded in the network, and BZDs are prescribed in different circumstances from SSRIs, so the groups may differ in ways matching cannot capture. The TCGA analyses used bulk tumor tissue from patients not treated with ICIs, so they cannot localize SLC6A4 to particular cells or show that SSRIs alter the tumor microenvironment.

**Conclusions:**

In this study, SSRI use was associated with lower mortality and organ-selective immune activation compared with BZD use among ICI-treated patients with psychiatric comorbidity. Transcriptomic evidence supports biological plausibility. These findings warrant prospective evaluation of SSRI repurposing as an ICI adjuvant.

## Author summary

**Why was this study done?:**

- Depression and anxiety affect 20–40% of people with cancer who receive immunotherapy, and the psychiatric medicines prescribed for them differ in how they act on the immune system.
- Laboratory studies have shown that the serotonin transporter acts as a brake on cancer-fighting T cells, and that selective serotonin reuptake inhibitors (SSRIs) release that brake in mice treated with immunotherapy.
- It was not known whether the choice between an SSRI and a benzodiazepine, the other psychiatric medicine commonly prescribed in this setting, is related to how people fare on immunotherapy.

**What did the researchers do and find?:**

- Using electronic health records from a large international network, we compared 1,567 people taking an SSRI with 1,567 people taking a benzodiazepine, all of whom had a solid tumor and depression or anxiety and were beginning immunotherapy, matched on 49 clinical characteristics.
- Over 2 years, 23.5% of the SSRI group died compared with 34.4% of the benzodiazepine group, and the SSRI group also had fewer newly recorded secondary tumors and fewer referrals to hospital and to palliative care.
- The SSRI group had more immune-related side effects overall, almost entirely thyroid problems, with no increase in inflammation of the liver, lungs, or bowel, and in tumor tissue from more than 8,000 cancers in a separate group of patients, higher activity of the serotonin transporter gene went with fewer immune cells inside the tumor.

**What do these findings mean?:**

- SSRIs, which are already widely prescribed to people with cancer, were associated with better outcomes during immunotherapy, and both the pattern of side effects and the tumor gene activity data are consistent with an effect on the immune system; a randomized trial is needed to test this directly.
- Because people were not randomly assigned to receive one medicine or the other, these results show an association and cannot show that SSRIs caused the better outcomes.
- The records do not capture how well or unwell a person was when treatment started, and benzodiazepines are often given at times of acute distress or worsening illness, so part of the difference may reflect how ill people were rather than the medicine itself.

## Abstract 3

In this target trial emulation, Po-Huang Chen and colleagues compare outcomes between selective serotonin reuptake inhibitor and benzodiazepine users with solid tumors and depression or anxiety and find that patients receiving immune checkpoint inhibitors who initiated selective serotonin reuptake inhibitors had lower mortality than those initiating benzodiazepines.

## Introduction

Immune checkpoint inhibitors (ICIs) have transformed treatment across multiple solid tumor types, yet only a fraction of patients achieve durable responses [1]. Depression and anxiety affect 20–40% of patients with cancer who receive immunotherapy [2] and are associated with worse treatment outcomes and survival [3]; both depression and serotonergic signaling are increasingly recognized to interact with immune function [4,5], underscoring the clinical importance of psychotropic medication choices in this population.

A landmark preclinical study by Li and colleagues [6] identified the serotonin transporter (SERT, encoded by SLC6A4) as an immune checkpoint on CD8+ T cells. SERT-mediated serotonin reuptake suppressed T-cell receptor signaling through the 5-hydroxytryptamine receptor (5-HTR) and mitogen-activated protein kinase (MAPK) pathway, while pharmacologic SERT inhibition with selective serotonin reuptake inhibitors (SSRIs) restored intratumoral serotonin and enhanced antitumor immunity. In murine models, SSRI combined with anti-programmed cell death protein 1 (anti-PD-1) therapy produced synergistic tumor regression, establishing a direct mechanistic link between serotonergic signaling and checkpoint blockade efficacy. These findings were independently supported by studies demonstrating enhanced immunosurveillance through serotonergic mechanisms, including peripheral serotonin attenuation [7], pleiotropic SSRI-mediated immune enhancement [8], and tumor-associated macrophage modulation [9].

Because SLC6A4 encodes the molecular target of SSRIs and the principal route by which serotonin is cleared from the tumor microenvironment, it is the logical node linking serotonergic signaling to checkpoint immunity, and, among the genes of the serotonin pathway, the one most likely to be modulated by routine clinical SSRI exposure.

Clinical evidence for this SSRI-ICI interaction remains limited. Magagnoli and colleagues [10] reported a suggestive survival benefit with fluoxetine among Veterans Affairs patients receiving PD-1/programmed death ligand 1 (PD-L1) therapy, but the analysis included only 34 exposed patients. Observational studies have linked emotional distress to worse ICI outcomes and reduced immune-related adverse event (irAE) incidence [11,12], but none have directly compared serotonergic versus nonserotonergic psychotropic agents in a design that reduces confounding by indication through an active comparator. Large-scale clinical evidence with active-comparator controls and translational validation is therefore lacking, leaving the question unresolved between preclinical discovery and clinical application.

Because no randomized trial has compared serotonergic with nonserotonergic psychotropic agents in this setting, we used target trial emulation [13], an approach that applies the design principles of a randomized trial, namely explicit eligibility, defined treatment strategies, a common time zero, and a prespecified estimand, to observational data, reducing biases such as immortal-time and prevalent-user bias and making the intended comparison explicit when a randomized trial is unavailable. Because treatment was not randomly assigned, the resulting estimates remain associations rather than randomized comparisons.

Here, we report a target trial emulation comparing SSRI versus benzodiazepine (BZD) use among 3,134 propensity score-matched patients with a solid tumor and depression or anxiety who were receiving ICIs, drawn from the TriNetX federated electronic health record network. The active-comparator design isolates the serotonergic mechanism by contrasting SERT inhibition (SSRI) against gamma-aminobutyric acid (GABA) signaling (BZD) in patients with equivalent psychiatric comorbidity. Twenty prespecified outcomes organized across 4 mechanistic pathways (immune enhancement, disease trajectory, BZD-specific harm, and negative controls) are used to discriminate among competing mechanistic explanations. To investigate the biological basis of these clinical findings, we examine SLC6A4 expression and immune microenvironment features across 20 solid tumor types in The Cancer Genome Atlas (TCGA), including an organ-level cross-validation linking tissue-specific SLC6A4-immune associations to clinical irAE patterns. We asked whether the choice of psychotropic agent at ICI initiation is associated with subsequent outcomes, and hypothesized that, among patients with depression or anxiety who initiate an ICI, initiation of an SSRI rather than a benzodiazepine is associated with lower 2-year all-cause mortality, and that any such association is accompanied by an organ-selective increase in immune-related adverse events in the tissues where SLC6A4 expression tracks most closely with reduced immune-cell infiltration.

## Methods

### Study design and data source

This retrospective cohort study used an active-comparator, new-user design [14] within the TriNetX Global Collaborative Network, a federated electronic health record (EHR) platform integrating de-identified data from over 187 million patients across 172 healthcare organizations. We compared outcomes between SSRI and benzodiazepine (BZD) users among patients with a solid tumor and depression or anxiety who received immune checkpoint inhibitor (ICI) therapy between January 2015 and December 2025. The active-comparator design reduces confounding by indication [15], as both groups carry psychiatric diagnoses and receive psychotropic treatment, while providing a mechanistic contrast between serotonergic (SERT inhibition) and GABAergic (GABA-A agonism) pathways. This study is reported in accordance with the STROBE reporting guideline [16] (S1 Checklist) and the TARGET guideline for emulating a target trial [17] (S2 Checklist). The study was approved by the Institutional Review Board of Tri-Service General Hospital, National Defense Medical Center (TSGHIRB No. E202516013), with a certificate of review exemption.

The hypothetical target trial and its emulation are specified in full in the pre-specified analysis plan (S1 Protocol): eligible adults with a solid tumor and a documented depressive or anxiety disorder would be randomly assigned, at the time of first ICI administration (time zero), to initiate an SSRI or a benzodiazepine and followed for overall survival and prespecified secondary outcomes under an intention-to-treat estimand. In the emulation, randomization is replaced by 1:1 propensity score matching on 49 baseline covariates, time zero is the first ICI administration, and follow-up, outcomes, and the causal contrast are operationalized from the electronic health record. Variables that a trial would balance but that are unmeasured here, notably Eastern Cooperative Oncology Group (ECOG) performance status, PD-L1 expression, tumor mutational burden, and microsatellite-instability status, are addressed through negative-control outcomes, *E*-values, and the sensitivity analyses described below. The target trial and its emulation are also specified component by component in S1 Protocol (Section 3) and shown schematically in Fig 1.

**Fig 1:**
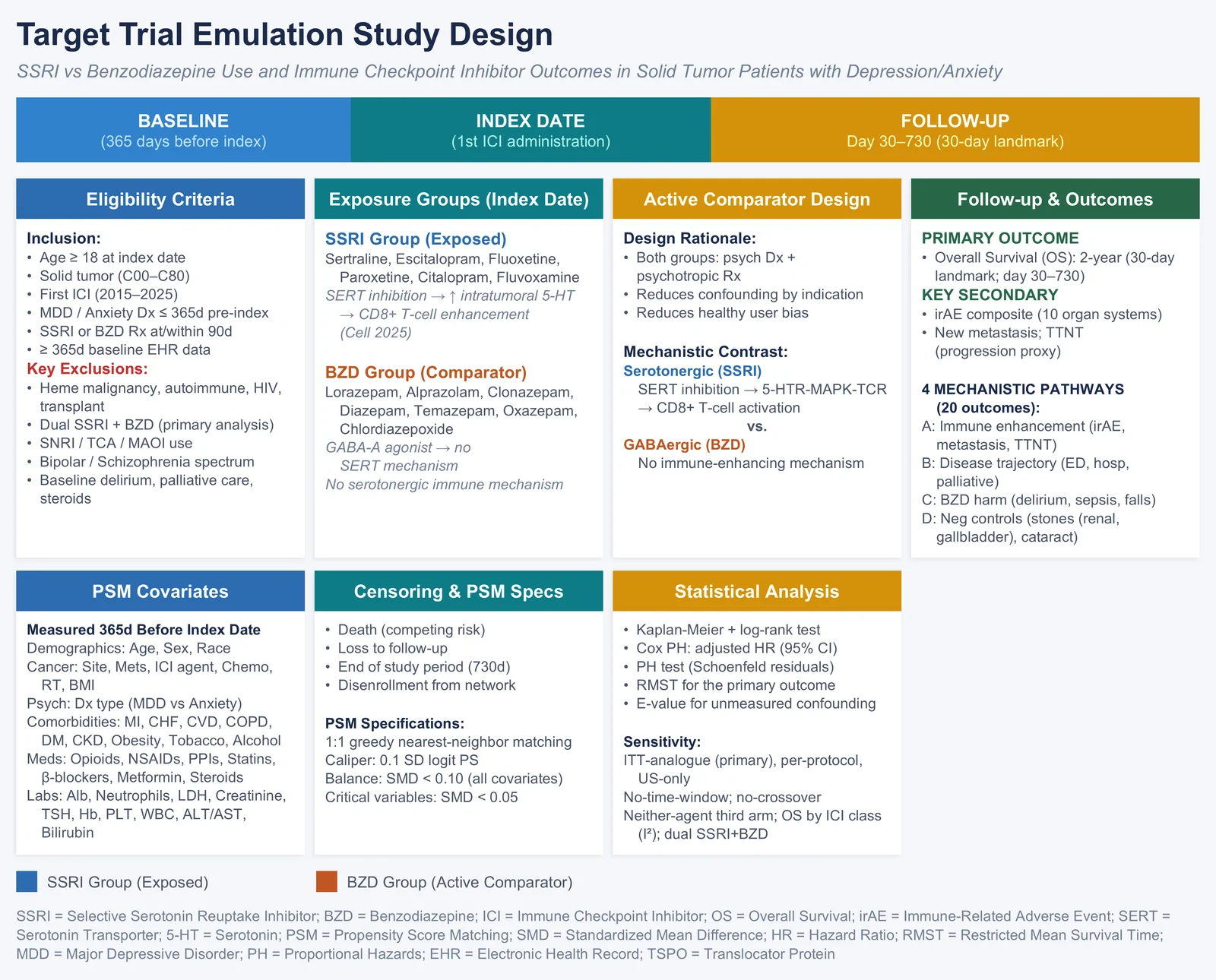
Target trial emulation study design. Schematic diagram of the target trial emulation framework comparing SSRI with benzodiazepine use among patients with a solid tumor and depression or anxiety who were receiving immune checkpoint inhibitors. The study timeline is divided into baseline (365 days before the index date), the index date (first ICI administration, time zero), and follow-up (days 30 to 730 after the index date, beginning at the prespecified 30-day landmark). Abbreviations: BZD, benzodiazepine; ICI, immune checkpoint inhibitor; SSRI, selective serotonin reuptake inhibitor.

### Study population

Patients were eligible if they met all of the following criteria: (1) age 18 years or older at the index date; (2) a solid malignant neoplasm (International Classification of Diseases, Tenth Revision, Clinical Modification [ICD-10-CM] C00-C80) documented on or before the index date; (3) a first administration of any of the 13 US Food and Drug Administration-approved immune checkpoint inhibitors listed in S8 Table (including pembrolizumab, nivolumab, atezolizumab, durvalumab, and ipilimumab) between January 1, 2015 and December 31, 2025, which defined the index date (time zero); (4) major depressive disorder (F32, F33), generalized anxiety disorder (F41.1), or anxiety disorder not otherwise specified (F41.9) recorded within 365 days before the index date; (5) an active prescription for an SSRI or a benzodiazepine within 90 days before to 14 days after the index date; (6) at least 365 days of electronic health record data before the index date; and (7) at least 1 day of follow-up after the index date. The SSRI group comprised users of sertraline, escitalopram, fluoxetine, paroxetine, citalopram, or fluvoxamine without concurrent benzodiazepine use, and the comparator group comprised users of lorazepam, alprazolam, clonazepam, diazepam, temazepam, oxazepam, or chlordiazepoxide without concurrent antidepressant use (S8 and S9 Tables). Exposure was therefore ascertained from an active prescription in the window from 90 days before to 14 days after the index date: patients were new users of ICI therapy, and their psychotropic treatment could already have been in place before the first ICI infusion rather than starting on that day.

Patients were excluded if they had any of the following: (1) hematologic malignancy (C81-C96) at any time before the index date; (2) pre-existing autoimmune disease requiring immunosuppressive therapy; (3) HIV infection (B20) at any time; (4) a history of solid organ or hematopoietic stem cell transplantation (Z94); (5) a schizophrenia spectrum disorder (F20-F29) or bipolar disorder (F30-F31) at any time; (6) systemic corticosteroid therapy at 10 mg or more of prednisone equivalent per day for 14 days or longer within the 30 days before the index date; (7) delirium (F05) within 30 days before the index date; (8) palliative care (Z51.5) within 30 days before the index date; (9) concurrent prescriptions for both an SSRI and a benzodiazepine overlapping the index date; or (10) a prescription for any non-SSRI antidepressant (serotonin-norepinephrine reuptake inhibitor, tricyclic antidepressant, or monoamine oxidase inhibitor) overlapping the index date. Mutual exclusivity of SSRI and benzodiazepine use was assessed at baseline, within the exposure window of 90 days before to 14 days after ICI initiation, rather than across the entire follow-up period. Sequential application of these criteria is shown in Fig 2, and the operational code lists are provided in S7–S9 Tables.

**Fig 2:**
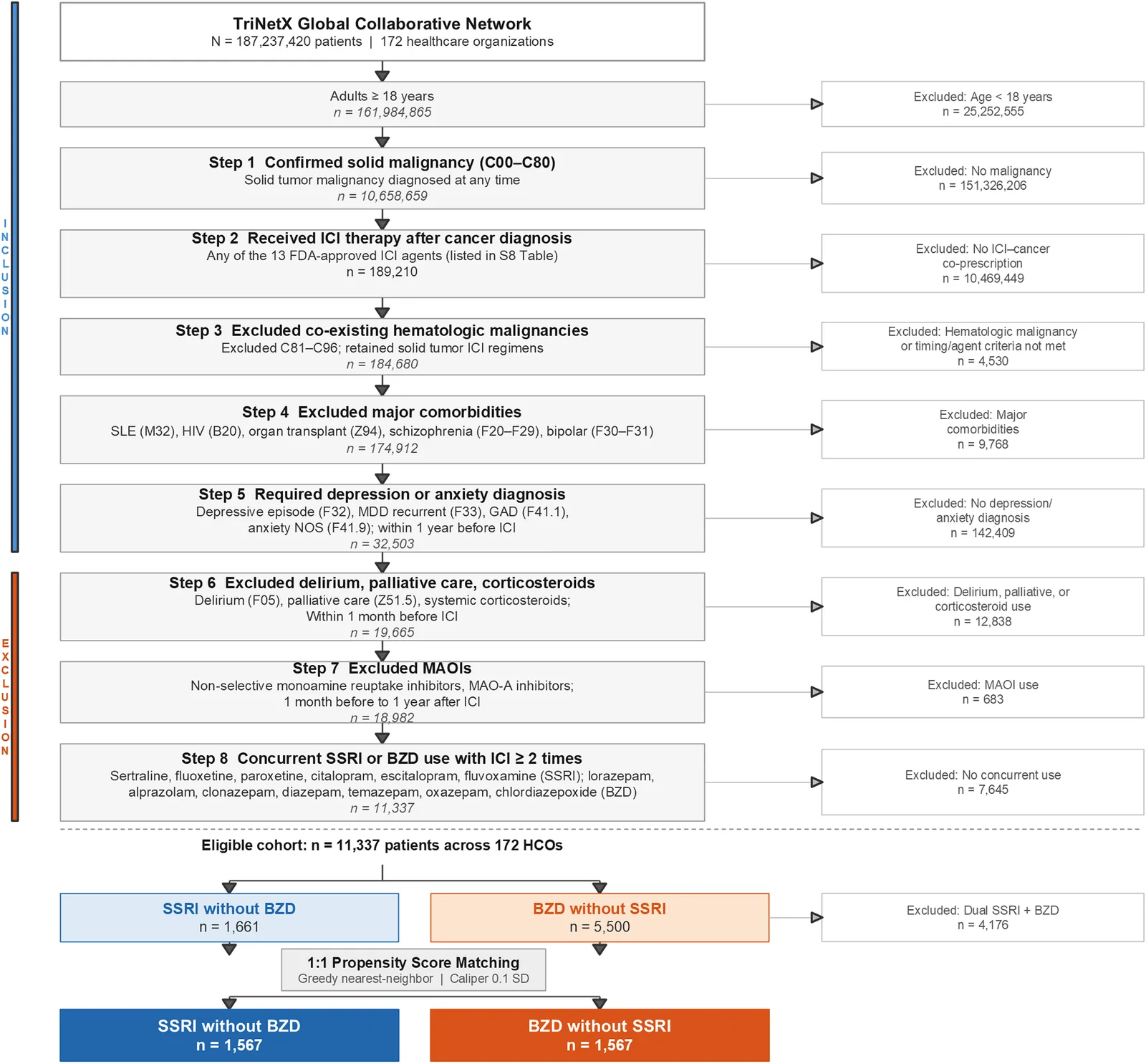
Patient selection flow diagram. Sequential application of the inclusion and exclusion criteria within the TriNetX Global Collaborative Network (*N* = 187,237,420 patients; 172 healthcare organizations). Eight selection steps yielded 11,337 eligible patients. After exclusion of 4,176 patients with concurrent SSRI and benzodiazepine use, 1,661 SSRI-only and 5,500 benzodiazepine-only patients remained. Propensity score matching (1:1, caliper 0.1 SD of the logit propensity score) produced 1,567 matched pairs. Abbreviations: BZD, benzodiazepine; ICI, immune checkpoint inhibitor; SSRI, selective serotonin reuptake inhibitor.

### Propensity score matching

One-to-one greedy nearest-neighbor matching (caliper, 0.1 SD of the logit propensity score) [18] included 49 covariates: demographics, tumor characteristics, psychiatric diagnosis type, 12 medical comorbidities, 11 baseline medications, 11 laboratory values, and 3 baseline treatment and clinical variables (chemotherapy, radiotherapy, and body-mass index) (S7 Table). Adequate balance was confirmed with all standardized mean differences (SMDs) less than 0.10.

### Outcomes

The primary outcome was overall survival (time from first ICI to death; 2-year window). Follow-up began on day 30 after ICI initiation, a prespecified 30-day landmark that aligns the survival window with the new-diagnosis outcome window and reduces immortal-time and early-death bias [19], and continued until death, last EHR contact, or day 730, whichever came first. The 2-year window was prespecified as the clinically relevant horizon for first-line ICI benefit and to harmonize follow-up across organizations with differing data maturity; a sensitivity analysis without time-window restriction was also performed. Twenty secondary outcomes were organized across 4 prespecified mechanistic pathways, which label the hypothesized mechanisms the study was designed to discriminate among and are not claims of established causation: path A (SSRI immune enhancement), path B (better disease trajectory), path C (BZD-specific harm), and path D (negative controls). Path A comprised 6 outcomes: thyroid dysfunction (new thyroid immune-related adverse event; E03, E06), a composite of new immune-related adverse events across 10 organ systems, a new systemic corticosteroid prescription, newly coded distant metastasis (C77, C78, C79), time to next treatment, and the number of ICI administrations during follow-up. Path B comprised 6 outcomes: an emergency department visit, unplanned hospitalization, palliative or hospice referral (Z51.5), a new psychiatric diagnosis (F32, F33, F41, F43), opioid dose escalation, and suicidal ideation or self-harm (R45.851, T14.91, X71-X83). Path C comprised 5 outcomes: falls (W00-W19, R29.6), fractures (traumatic or osteoporotic fragility), delirium (F05, R41.0, R41.82), sepsis or severe infection (A41, R65.2, A49), and venous thromboembolism (I26, I82). Path D comprised 3 outcomes without biological plausibility for differential SSRI-BZD association, serving as bias indicators: nephrolithiasis (N20), cholelithiasis (K80), and cataract (H25, H26). Unless otherwise specified, outcomes were ascertained between days 30 and 730 after ICI initiation; the per-outcome ascertainment rules and the complete code lists are given in S10 and S11 Tables.

As a progression-sensitive proxy, because radiographic progression-free survival cannot be ascertained in this network, we defined time to next treatment as the time from ICI initiation to the start of a new systemic anticancer agent that was not part of the baseline regimen (chemotherapy, a protein kinase inhibitor, or a non-ICI therapeutic monoclonal antibody or antibody-drug conjugate), with continued or re-initiated ICI not counted as an event and death censored. To assess whether differential surveillance could account for differences in newly coded metastases, we also compared the per-patient frequency of cross-sectional imaging (computed tomography, magnetic resonance imaging, positron emission tomography-computed tomography, and bone scintigraphy) between groups during follow-up.

### Statistical analysis

Time-to-event outcomes were analyzed using Kaplan-Meier estimation with log-rank tests and Cox proportional hazards regression. The single primary outcome (overall survival) was prespecified; secondary-outcome *p* values are descriptive and unadjusted for multiplicity, and secondary findings are interpreted as hypothesis-generating. The proportional hazards assumption was tested using Schoenfeld residuals, and restricted mean survival time (RMST) is reported for the primary outcome. Palliative or hospice referral (*p* = 0.032) and venous thromboembolism (*p* = 0.042) violated it, so their hazard ratios are period-averaged effects. E-values were calculated following VanderWeele and Ding [20] to quantify the minimum strength of association an unmeasured confounder would need with both exposure and outcome to explain away the observed result (S6 Table). Three prespecified sensitivity analyses were performed: per-protocol (*N* = 695 matched pairs), US-only (*N* = 1,450 pairs), and dual SSRI plus BZD versus BZD-only (*N* = 2,457 pairs).

The primary analysis estimates the intention-to-treat-analogue effect of SSRI versus benzodiazepine treatment at ICI initiation, consistent with the pre-specified analysis plan; the per-protocol analysis is a sensitivity analysis. We prioritized the intention-to-treat analogue because it matches the pre-specified estimand and does not require censoring at treatment switching, which on this federated platform cannot be corrected by inverse-probability-of-censoring weighting because patient-level data cannot be exported. Additional sensitivity analyses, prompted in part by peer review, comprised overall survival with no time-window restriction, exclusion of patients who initiated the opposite drug class during follow-up, a third comparator arm of patients receiving neither an SSRI nor a benzodiazepine matched 1:1 on the same covariates, and overall survival estimated within each ICI class with between-class heterogeneity assessed by the I^2^ statistic.

Prespecified and post hoc analyses. The hypotheses, outcomes, and analytical methods described above were fixed in the analysis plan (S1 Protocol) before data extraction. The prespecified primary hypothesis was that, among patients with depression or anxiety who initiate an ICI, initiation of an SSRI rather than a benzodiazepine is associated with lower 2-year all-cause mortality. The prespecified secondary hypotheses were that SSRI use is associated with more frequent immune-related adverse events and better tumor control (path A), a more favorable disease trajectory (path B), and fewer benzodiazepine-specific harms (path C), with 3 negative control outcomes specified as bias indicators (path D). The planned analyses were 1:1 propensity score matching followed by Kaplan–Meier estimation, log-rank testing, and Cox proportional hazards regression, with per-protocol, US-only, and dual-exposure sensitivity analyses, *E*-values, and prespecified subgroup analyses. All of these analyses were performed as planned, without deviation. Seven further analyses were added during peer review and are therefore post hoc rather than prespecified: overall survival without a time-window restriction; overall survival excluding patients who initiated the opposite drug class during follow-up; the comparison with a third arm of patients receiving neither agent; overall survival within each ICI class; the refined time-to-next-treatment endpoint; the comparison of imaging-surveillance frequency; and the extension of the transcriptomic analysis to the full 20-gene serotonin pathway. These 7 analyses are listed as post hoc in S1 Protocol (Section 11) and are interpreted as hypothesis-generating and supportive rather than confirmatory. No analysis reported here was chosen on the basis of an unanticipated pattern in the data.

### TCGA pan-cancer transcriptomic analyses

To assess whether the serotonin transporter gene SLC6A4 associates with the extent of immune-cell infiltration across human cancers, we analyzed RNA-seq data from 8,272 tumor samples contributed by 8,172 patients across 20 solid tumor types in The Cancer Genome Atlas (TCGA); 100 patients contributed more than one tumor sample, and no patient appeared in more than one tumor type. Single-sample gene set enrichment analysis (ssGSEA) [21] was used to compute immune function scores (T-cell Inflamed Gene Expression Profile [GEP] [22], Cytotoxic Score [23]) and classify tumors into 3 immune phenotypes (Inflamed, Excluded, and Desert) [24]. Spearman correlations between SLC6A4 expression and immune scores were computed per cancer type with Benjamini–Hochberg false discovery rate (FDR) correction. Organ-level concordance was assessed by mapping tissue-specific SLC6A4-immune correlations to clinical organ-specific irAE hazard ratios for 5 organ systems with direct anatomical correspondence. A random-effects meta-analysis (restricted maximum likelihood estimator, Hartung–Knapp confidence interval) of SLC6A4 expression and overall survival was performed across 18 evaluable cancer types. Full details are provided in the S1 Text and S13, S14 Tables.

To test whether the focus on SLC6A4 was justified rather than arbitrary, we extended the transcriptomic analysis to the entire serotonin pathway, computing the correlation of each of 20 pathway genes (the transporter SLC6A4, 14 serotonin receptors, the synthesis enzymes TPH1, TPH2, and DDC, and the degradation enzymes MAOA and MAOB) with three orthogonal immune signatures across the 20 tumor types, with Benjamini–Hochberg correction. The TCGA analyses are cross-sectional and use bulk tumor tissue from patients not treated with ICIs, so they cannot localize SLC6A4 to particular cells or show that SSRIs alter the tumor microenvironment.

### Use of artificial intelligence tools

Claude (Anthropic) was used, through the Claude Code interface between 2025 and August 2026, for language editing of the manuscript text and for assistance in writing and debugging the R code used in the transcriptomic analyses; successive releases of the Claude Opus and Claude Sonnet models were used over this period, most recently Claude Opus 5. No AI tool was used to generate, extract, or interpret the clinical or transcriptomic data. All AI-assisted text was checked against the underlying results and revised by the authors, and all AI-assisted code was executed by the authors and its output verified against the source data. The authors retain full responsibility for the content of this manuscript, including its analyses, results, interpretation, and conclusions.

## Results

### Study cohort and primary outcome

From 187,237,420 patients in the TriNetX Global Collaborative Network, 11,337 met eligibility criteria (Fig 2). After excluding 4,176 dual SSRI plus BZD users, propensity score matching yielded 1,567 pairs (Table 1). All covariates were well balanced (all SMD < 0.10; complete covariate balance in S20 Table). Median follow-up was 395 days (SSRI) and 349 days (BZD).

**Table 1:** Baseline characteristics before and after propensity score matching.

Characteristic	Before matching				After matching			
	SSRI (*n* = 1,661)	BZD (*n* = 5,500)	*p value*	SMD	SSRI (*n* = 1,567)	BZD (*n* = 1,567)	*p value*	SMD
***Demographics***								
Age at index date, yr, mean ± s.d.	66.9 ± 12.4	63.2 ± 12.3	<0.001	0.301	66.4 ± 12.4	66.5 ± 11.2	0.933	0.003
*Sex*								
Female	926 (55.7)	3,137 (57.0)	0.354	0.026	880 (56.2)	893 (57.0)	0.639	0.017
Male	735 (44.3)	2,363 (43.0)	0.354	0.026	687 (43.8)	674 (43.0)	0.639	0.017
*Race or ethnic group*								
White	1,396 (84.0)	4,355 (79.2)	<0.001	0.126	1,314 (83.9)	1,316 (84.0)	0.923	0.003
Black or African American	115 (6.9)	464 (8.4)	0.047	0.057	111 (7.1)	107 (6.8)	0.779	0.010
Asian	28 (1.7)	287 (5.2)	<0.001	0.194	28 (1.8)	26 (1.7)	0.784	0.010
Unknown or other	76 (4.6)	248 (4.5)	0.909	0.003	70 (4.5)	71 (4.5)	0.931	0.003
***Primary tumor site***								
Head and neck	102 (6.1)	436 (7.9)	0.015	0.070	102 (6.5)	105 (6.7)	0.829	0.008
Gastrointestinal tract	349 (21.0)	1,081 (19.7)	0.225	0.034	327 (20.9)	329 (21.0)	0.930	0.003
Thoracic	543 (32.7)	2,283 (41.5)	<0.001	0.183	534 (34.1)	545 (34.8)	0.679	0.015
Melanoma or skin	351 (21.1)	939 (17.1)	<0.001	0.103	333 (21.3)	330 (21.1)	0.896	0.005
Breast	133 (8.0)	569 (10.3)	0.005	0.081	131 (8.4)	141 (9.0)	0.526	0.023
Genitourinary	370 (22.3)	790 (14.4)	<0.001	0.206	324 (20.7)	315 (20.1)	0.690	0.014
***Metastatic disease at baseline***								
Visceral metastasis	434 (26.1)	1,774 (32.3)	<0.001	0.135	419 (26.7)	417 (26.6)	0.936	0.003
Other distant metastasis	483 (29.1)	2,083 (37.9)	<0.001	0.187	473 (30.2)	446 (28.5)	0.289	0.038
***Psychiatric diagnosis***								
Depressive disorder	1,193 (71.8)	2,212 (40.2)	<0.001	0.672	1,099 (70.1)	1,091 (69.6)	0.755	0.011
Anxiety disorder	1,018 (61.3)	4,641 (84.4)	<0.001	0.538	1,012 (64.6)	1,007 (64.3)	0.852	0.007
***Comorbidities***								
Ischemic heart disease	469 (28.2)	1,308 (23.8)	<0.001	0.102	437 (27.9)	431 (27.5)	0.811	0.009
Heart failure	216 (13.0)	596 (10.8)	0.015	0.067	201 (12.8)	194 (12.4)	0.706	0.013
Cerebrovascular disease	258 (15.5)	700 (12.7)	0.003	0.081	239 (15.3)	255 (16.3)	0.433	0.028
Dementia	45 (2.7)	70 (1.3)	<0.001	0.103	40 (2.6)	39 (2.5)	0.909	0.004
COPD	401 (24.1)	1,299 (23.6)	0.660	0.012	384 (24.5)	389 (24.8)	0.836	0.007
Chronic liver disease	331 (19.9)	1,162 (21.1)	0.292	0.030	317 (20.2)	315 (20.1)	0.929	0.003
Type 2 diabetes mellitus	505 (30.4)	1,226 (22.3)	<0.001	0.185	453 (28.9)	459 (29.3)	0.813	0.008
Chronic kidney disease	347 (20.9)	823 (15.0)	<0.001	0.155	313 (20.0)	297 (19.0)	0.470	0.026
Overweight or obesity	422 (25.4)	1,059 (19.3)	<0.001	0.148	389 (24.8)	370 (23.6)	0.428	0.028
Tobacco use disorder	413 (24.9)	1,545 (28.1)	0.010	0.073	403 (25.7)	414 (26.4)	0.654	0.016
Alcohol use disorder	134 (8.1)	375 (6.8)	0.082	0.048	125 (8.0)	131 (8.4)	0.696	0.014
***Baseline treatment and clinical data***								
Systemic chemotherapy	1,655 (99.6)	5,474 (99.5)	0.550	0.017	1,561 (99.6)	1,561 (99.6)	1.000	<0.001
Radiation therapy	420 (25.3)	1,807 (32.9)	<0.001	0.167	413 (26.4)	413 (26.4)	1.000	<0.001
Body-mass index, kg/m², mean ± s.d.	28.1 ± 7.1	27.0 ± 6.9	<0.001	0.150	28.0 ± 7.2	27.7 ± 7.1	0.340	0.037

Data are *n* (%) unless otherwise indicated. SSRI: selective serotonin reuptake inhibitor without concurrent BZD; BZD: benzodiazepine without concurrent SSRI.

*p* values: chi-squared test (categorical) or independent-samples *t* test (continuous). SMD < 0.10 indicates adequate balance; operational ICD-10-CM definitions for all baseline covariates are provided in S7 Table; the complete covariate balance table is provided in S20 Table.

Abbreviations: BZD, benzodiazepine; COPD, chronic obstructive pulmonary disease; s.d., standard deviation; SMD, standardized mean difference; SSRI, selective serotonin reuptake inhibitor.

SSRI use was associated with significantly lower 2-year all-cause mortality compared with BZD use (368 of 1,567 [23.5%] versus 539 of 1,567 [34.4%]; hazard ratio [HR] 0.627; 95% confidence interval [CI] [0.549, 0.716]; *p* < 0.001; Fig 3). The proportional hazards assumption was met (Schoenfeld *p* = 0.825). The Kaplan–Meier estimated absolute risk difference increased from 3.0% at 3 months to 14.9% at 24 months. RMST analysis showed a 2.1-month difference in restricted mean survival time (SSRI, 19.1 months versus BZD, 17.0 months; S2 Fig). The *E*-value for the primary outcome was 2.57 (95% CI bound, 2.14).

**Fig 3:**
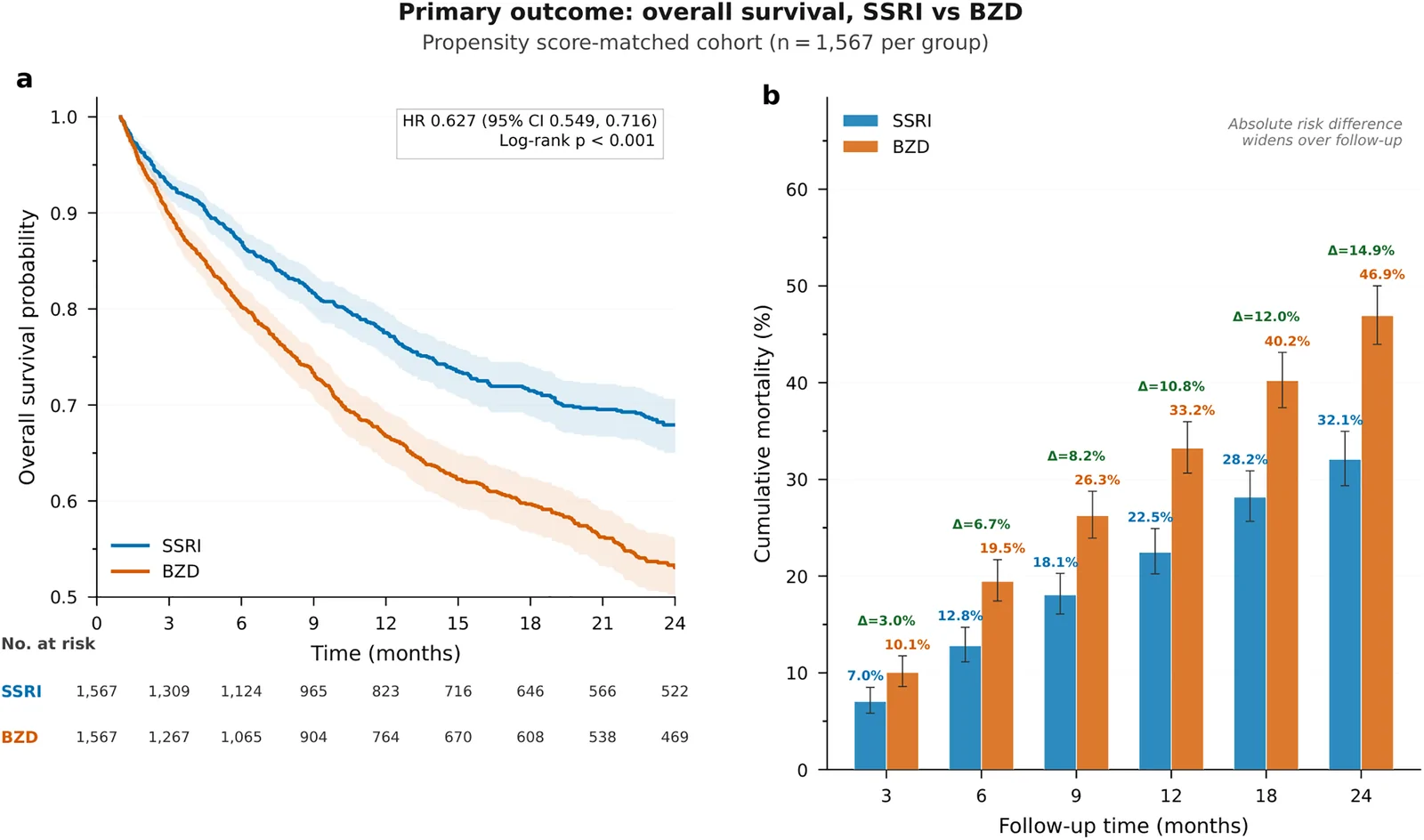
Primary outcome: Overall survival. **a**, Kaplan–Meier survival curves for SSRI (blue) vs. BZD (orange) cohorts with 95% CIs and number-at-risk table. HR 0.627; 95% CI [0.549, 0.716]; log-rank *p* < 0.001. **b**, Cumulative mortality at prespecified time points with absolute risk differences. The absolute difference in survival increased from 3.0% at 3 months to 14.9% at 24 months. Panel *b* values are Kaplan–Meier estimates, which account for censoring and are therefore higher than the crude proportions reported in the Abstract and Table 2. *N* = 1,567 propensity score-matched pairs per group.

### Mechanistic pathway outcomes and organ-selective irAE

Twenty secondary outcomes were assessed across 4 pathways (Fig 4 and S5 Table; key outcomes summarized in Table 2).

**Fig 4:**
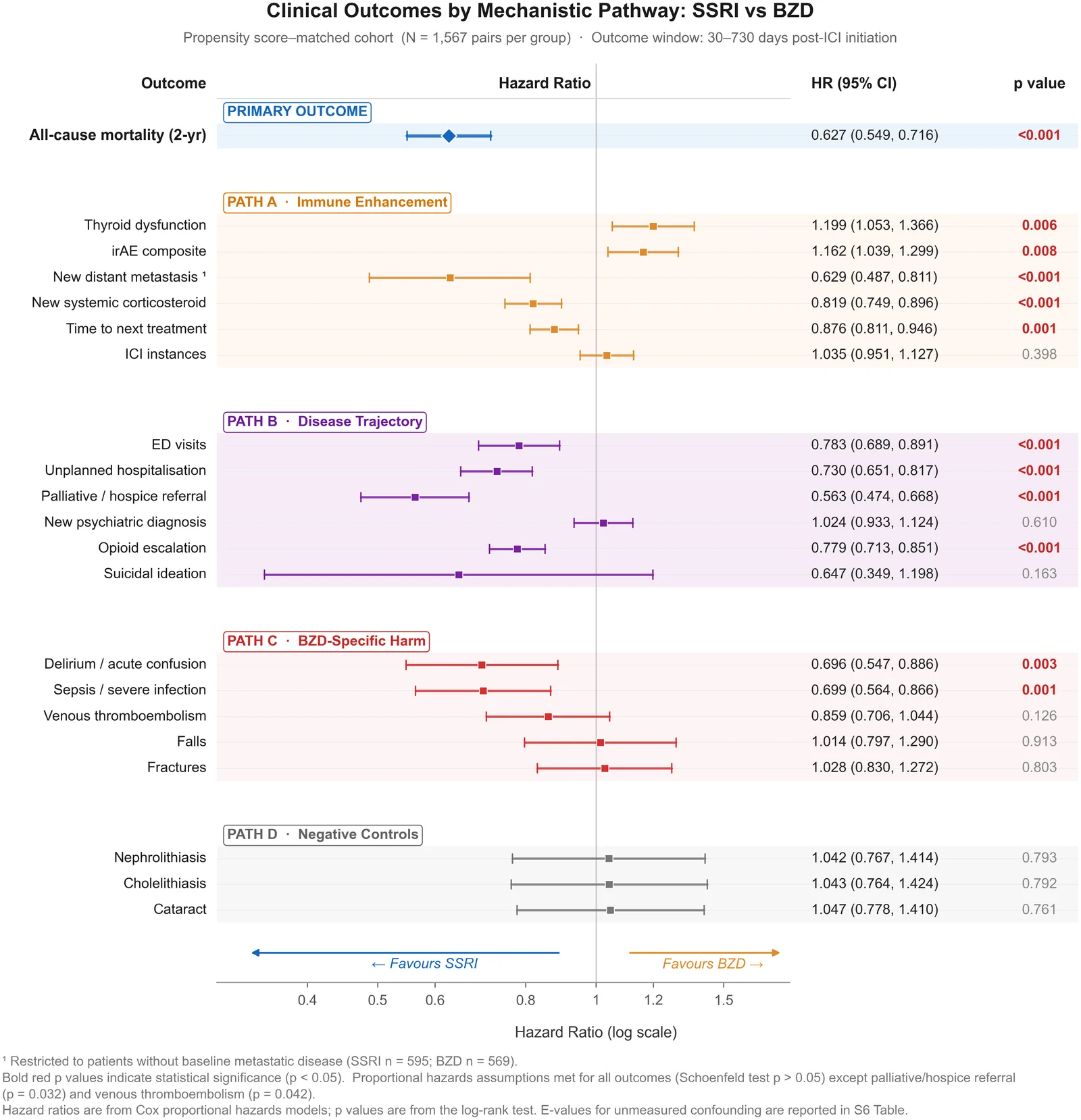
Clinical outcomes by mechanistic pathway. Forest plot of hazard ratios with 95% CIs for 20 prespecified secondary outcomes plus the primary outcome (all-cause mortality), organized by 4 mechanistic pathways: path A (SSRI immune enhancement), path B (disease trajectory), path C (BZD-specific harm), and path D (negative controls). Outcome window: 30 to 730 days after first ICI administration. *N* = 1,567 pairs per group. Restricted denominator for new distant metastasis (SSRI, *n* = 595; BZD, *n* = 569). Bold *p* values indicate *p* < 0.05. Hazard ratios are from Cox proportional hazards models; *p* values are from the log-rank test.

**Table 2:** Clinical outcomes after propensity score matching, organized by prespecified mechanistic pathways.

Outcome	Events, *n/N* (%)		Effect estimates		*p* value	*E*-value (95% CI)
	SSRI group	BZD group	Risk ratio (95% CI)	Hazard ratio (95% CI)		
**Primary outcome**						
All-cause mortality	368/1,567 (23.5)	539/1,567 (34.4)	0.683 (0.610, 0.764)	0.627 (0.549, 0.716)	<0.001	2.57 (2.14)
**Pathway A: SSRI immune enhancement**						
Thyroid dysfunction	495/1,567 (31.6)	419/1,567 (26.7)	1.181 (1.059, 1.318)	1.199 (1.053, 1.366)	0.006	1.69 (1.29)
Immune-related adverse event (composite)	771/1,567 (49.2)	682/1,567 (43.5)	1.130 (1.048, 1.219)	1.162 (1.039, 1.299)	0.008	1.60 (1.24)
New distant metastasis †	102/595 (17.1)	142/569 (25.0)	0.687 (0.547, 0.862)	0.629 (0.487, 0.811)	<0.001	2.56 (1.77)
Time to next treatment (TTNT) ‡	1,273/1,567 (81.2)	1,308/1,567 (83.5)	**0.973 (0.942, 1.005)**	0.876 (0.811, 0.946)	0.001	**1.54 (1.30)**
**Pathway B: Disease trajectory and adherence**						
Palliative or hospice referral	211/1,567 (13.5)	344/1,567 (22.0)	0.613 (0.525, 0.717)	0.563 (0.474, 0.668)	<0.001	2.95 (2.36)
Unplanned hospitalization	543/1,567 (34.7)	668/1,567 (42.6)	0.813 (0.744, 0.889)	0.730 (0.651, 0.817)	<0.001	2.08 (1.75)
**Pathway C: BZD-specific harm**						
Delirium or acute confusional state	115/1,567 (7.3)	155/1,567 (9.9)	0.742 (0.589, 0.935)	0.696 (0.547, 0.886)	0.003	2.23 (1.51)
Sepsis or severe infection	146/1,567 (9.3)	196/1,567 (12.5)	0.745 (0.608, 0.912)	0.699 (0.564, 0.866)	0.001	2.22 (1.58)
**Pathway D: Negative control outcomes**						
Nephrolithiasis	86/1,567 (5.5)	79/1,567 (5.0)	1.089 (0.809, 1.466)	1.042 (0.767, 1.414)	0.793	1.25 (1.00)
Cholelithiasis	83/1,567 (5.3)	76/1,567 (4.9)	1.092 (0.806, 1.479)	1.043 (0.764, 1.424)	0.792	1.25 (1.00)
Cataract	91/1,567 (5.8)	83/1,567 (5.3)	1.096 (0.821, 1.464)	1.047 (0.778, 1.410)	0.761	1.27 (1.00)

Values are risk (%) within each 1:1 propensity-score–matched cohort (1,567 pairs per group). Hazard ratios (HR) are from Cox models comparing SSRI with BZD (HR < 1 = lower risk with SSRI); p from the log-rank test. Outcome window: day 30–730 after ICI initiation (prespecified 30-day landmark). Hazard ratios are estimated within the propensity score-matched cohorts and are therefore adjusted for the 49 baseline covariates used in matching (demographics, tumor characteristics, psychiatric diagnosis type, 12 comorbidities, 11 medications, 11 laboratory values, and 3 baseline treatment and clinical variables; the complete list is given in S7 Table). Unadjusted comparisons of the two cohorts before matching are shown in Table 1, and the before-matching counterpart of every outcome estimate below is given in S21 Table.

† New distant metastasis: restricted to patients without baseline metastatic disease; surveillance-imaging frequency was comparable between groups (S17 Table; HR 0.957; 95% CI [0.875, 1.047]; *p* = 0.34), indicating the difference is not attributable to differential detection.

‡ TTNT (time to next treatment): time to a different antineoplastic agent, with continuation or re-dosing of the index ICI not counted as an event and death censored; a progression proxy, not Response Evaluation Criteria in Solid Tumors (RECIST)-based progression-free survival.

*E*-value: minimum strength of association an unmeasured confounder would need with both exposure and outcome to explain away the estimate; the 95% CI *E*-value uses the confidence bound nearest the null.

In path A (immune enhancement), composite irAE incidence was significantly higher with SSRIs (771 of 1,567 [49.2%] versus 682 of 1,567 [43.5%]; HR 1.162; 95% CI [1.039, 1.299]; *p* = 0.008). Thyroid dysfunction was the primary driver (HR 1.199; 95% CI [1.053, 1.366]; *p* = 0.006), while hepatitis (HR 0.711; 95% CI [0.474, 1.065]; *p* = 0.096), pneumonitis (HR 1.023; 95% CI [0.734, 1.427]; *p* = 0.892), and colitis (HR 1.130; 95% CI [0.913, 1.397]; *p* = 0.260) did not differ significantly (S4 Table). New distant metastases were significantly less frequent with SSRI use among patients without baseline metastases (102 of 595 [17.1%] versus 142 of 569 [25.0%]; HR 0.629; 95% CI [0.487, 0.811]; *p* < 0.001). Time to next treatment, a progression proxy, was also longer with SSRI use (HR 0.876; 95% CI [0.811, 0.946]; *p* = 0.001; Tables 2 and S5).

In path B (better disease trajectory), SSRI users had lower rates of emergency department visits (HR 0.783; 95% CI [0.689, 0.891]; *p* < 0.001), hospitalization (HR 0.730; 95% CI [0.651, 0.817]; *p* < 0.001), palliative or hospice referral (HR 0.563; 95% CI [0.474, 0.668]; *p* < 0.001), and opioid escalation (HR 0.779; 95% CI [0.713, 0.851]; *p* < 0.001). New psychiatric diagnoses did not differ (HR 1.024; 95% CI [0.933, 1.124]; *p* = 0.610).

In path C (BZD-specific harm), delirium (HR 0.696; 95% CI [0.547, 0.886]; *p* = 0.003) and sepsis (HR 0.699; 95% CI [0.564, 0.866]; *p* = 0.001) were significantly lower with SSRIs. Falls (HR 1.014; 95% CI [0.797, 1.290]; *p* = 0.913) and fractures (HR 1.028; 95% CI [0.830, 1.272]; *p* = 0.803) did not differ.

In path D (negative controls), all 3 outcomes showed null associations: nephrolithiasis (HR 1.042; 95% CI [0.767, 1.414]; *p* = 0.793), cholelithiasis (HR 1.043; 95% CI [0.764, 1.424]; *p* = 0.792), and cataract (HR 1.047; 95% CI [0.778, 1.410]; *p* = 0.761).

### Subgroup and sensitivity analyses

The association with lower mortality was consistent across prespecified subgroups (S1 Fig). The association was strongest among patients with comorbid depression and anxiety (HR 0.57; 95% CI [0.49, 0.68]; *p* < 0.001) and was observed across age groups and both sexes; a within-ICI-class analysis showed directionally consistent associations across classes. All 5 individually analyzable SSRIs were associated with significantly lower mortality (HR range 0.61 to 0.71; all *p* < 0.05), consistent with a class-wide serotonin transporter inhibition mechanism; fluvoxamine was not analyzed individually because fewer than 10 patients received it. Head and neck cancer was the only subgroup in which the estimate did not favor SSRI use (HR 1.08; 95% CI [0.69, 1.68]; *p* = 0.736).

The per-protocol analysis yielded a stronger effect (HR 0.575; 95% CI [0.472, 0.702]; *p* < 0.001; *N* = 695 pairs), and the US-only cohort showed consistent results (HR 0.581; 95% CI [0.507, 0.667]; *p* < 0.001; *N* = 1,450 pairs) (S1, S2 Tables).

The dual SSRI plus BZD analysis provided a mechanistic probe: concurrent users showed no mortality difference versus BZD-only users (HR 0.977; 95% CI [0.891, 1.071]; *p* = 0.624; *N* = 2,457 pairs) yet retained the irAE signal (HR 1.154; 95% CI [1.063, 1.253]; *p* = 0.001) (S3 Table).

Overall survival without any time-window restriction yielded a similar estimate (HR 0.596; 95% CI [0.532, 0.667]; S18 Table), and excluding patients who switched drug class during follow-up gave an HR of 0.668 (95% CI [0.583, 0.767]; S19 Table). Compared with patients receiving neither agent, SSRI use remained associated with lower mortality (HR 0.753; 95% CI [0.605, 0.939]; *p* = 0.011; S15 Table). The survival association was directionally consistent across ICI classes (anti-PD-1: HR 0.683; 95% CI [0.591, 0.790]; anti-PD-L1: HR 0.657; 95% CI [0.549, 0.786]; anti-cytotoxic T-lymphocyte-associated protein 4 (anti-CTLA-4): HR 0.692; 95% CI [0.451, 1.060]; combination: HR 0.890; 95% CI [0.536, 1.477]), with no detectable between-class heterogeneity (I^2^ = 0%; Cochran Q *p* = 0.75; S16 Table, S6 Fig); the anti-CTLA-4 and combination subgroups were underpowered, with confidence intervals crossing 1, so their class-specific estimates are exploratory. The frequency of surveillance imaging did not differ between groups (HR 0.957; 95% CI [0.875, 1.047]; *p* = 0.34; S17 Table), arguing against detection bias for the metastasis finding.

### SLC6A4 expression and tumor immune response across human cancers

SLC6A4 expression was negatively correlated with T-cell Inflamed GEP scores in 13 of 20 cancer types (FDR < 0.05; Fig 5A), with the strongest associations in bladder urothelial carcinoma (BLCA; rho = −0.430), thyroid carcinoma (THCA; rho = −0.335), and colon adenocarcinoma (COAD; rho = −0.326). An equivalent pattern was observed for Cytotoxic Scores (13 of 20 significant). Pancreatic adenocarcinoma (PAAD) was the sole exception, showing a positive correlation (rho = +0.165; FDR = 0.039).

**Fig 5:**
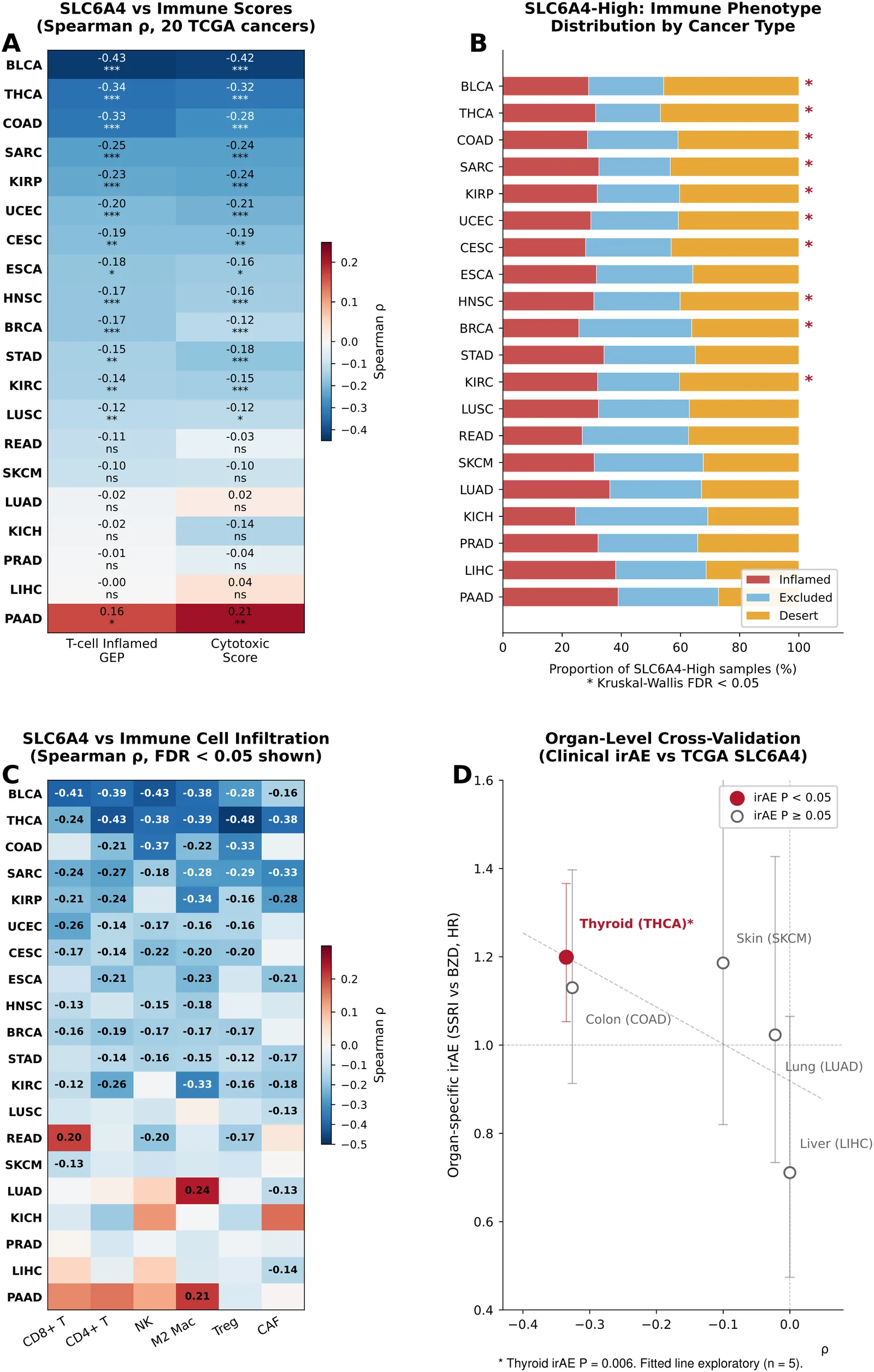
TCGA pan-cancer transcriptomic analyses and organ-level cross-validation. **A,** Spearman correlation between SLC6A4 expression and immune function scores (T-cell Inflamed GEP and Cytotoxic Score) across 20 TCGA solid tumor types. Asterisks denote FDR < 0.05. **B,** Distribution of immune phenotypes (Inflamed, Excluded, Desert) among SLC6A4-high samples by cancer type. Asterisks denote Kruskal-Wallis FDR < 0.05. **C,** Spearman correlation between SLC6A4 and 6 immune cell-type scores. Only values with FDR < 0.05 are displayed. **D,** Organ-level cross-validation: clinical irAE hazard ratios (y-axis) mapped to TCGA SLC6A4-immune correlation coefficients (x-axis) for 5 organ systems. Red point indicates thyroid irAE *p* < 0.05 (log-rank test). The fitted line is exploratory (*n* = 5).

In 10 of 20 cancer types with significant phenotype redistribution (Kruskal–Wallis FDR < 0.05), SLC6A4-high tumors were over-represented in the Immune Desert phenotype (Fig 5B). At the cell-type level, SLC6A4 was negatively correlated with CD8+ T-cell and natural killer (NK) cell infiltration, and, in most cancer types, with M2 macrophage and cancer-associated fibroblast signatures as well (Fig 5C).

SLC6A4 was not significantly associated with survival: the pooled HR across the 18 cancer types with sufficient survival events (of the 20 analyzed) was 1.04 (95% CI [0.94, 1.15]; *p* = 0.42; I^2^ = 13.0%), with only PAAD (HR 1.70; 95% CI [1.12, 2.57]; *p* = 0.011, low versus high expression) reaching significance (S3 Fig and S12 Table); in PAAD, higher SLC6A4 expression was associated with better survival (S4 Fig). This null finding is expected, as TCGA predates the immunotherapy era.

Across the serotonin pathway, SLC6A4 was the gene most consistently inversely correlated with immune activation: it ranked first of 20 genes by pooled correlation (median Spearman rho, −0.16), reaching significance in the negative direction in 37 of 60 gene-by-signature tests (20 genes across 3 immune signatures) and in the positive direction in only 2. The pattern was mechanistically coherent, with the serotonin-degrading enzyme MAOB correlating positively and most receptors showing weak or positive associations, indicating that the inverse association with immune signatures tracks specifically with the serotonin-handling transporter rather than the pathway at large (S5 Fig).

### Organ-level cross-validation

Mapping clinical irAE HRs to TCGA SLC6A4-immune correlations across 5 organs revealed concordance between reduced tumor immune-cell infiltration in the transcriptomic data and clinical immune activation (Fig 5D; S14 Table). The organ with the strongest clinical irAE enhancement (thyroid: HR 1.199; 95% CI [1.053, 1.366]; *p* = 0.006) corresponded to one of the strongest inverse SLC6A4-immune associations (THCA: rho = −0.335; FDR < 0.001). Conversely, the organ without clinical irAE enhancement (liver: HR 0.711; 95% CI [0.474, 1.065]; *p* = 0.096) corresponded to no SLC6A4-immune association (liver hepatocellular carcinoma [LIHC]: rho = −0.000; FDR = 0.997). Colon, skin, and lung fell along this gradient, with lung near the null for both measures.

## Discussion

In this target trial emulation of 3,134 patients with a solid tumor and depression or anxiety who were receiving ICIs, SSRI use was associated with a 37% lower hazard of death over 2 years compared with BZD use (HR 0.627; 95% CI [0.549, 0.716]). This mortality association was accompanied by increased immune-related adverse events, reduced metastatic progression, and a favorable disease trajectory including lower rates of hospitalization and palliative referral. Three null negative control outcomes, consistent results across per-protocol and US-only sensitivity analyses, and an *E*-value of 2.57 support the robustness of these findings.

The prespecified mechanistic pathway framework helps distinguish among competing mechanisms. Path A (immune enhancement) showed the most mechanistically informative pattern: composite irAE was significantly increased, driven selectively by thyroid dysfunction (HR 1.199; 95% CI [1.053, 1.366]) without significant increases in hepatitis, pneumonitis, or colitis. This organ selectivity suggests serotonergic modulation of T-cell activity concentrated in the hypothalamic-pituitary-thyroid axis [25,26] rather than generalized immune activation, and the absence of high-severity irAE increase provides a favorable safety signal. Reduced coded new metastases among patients without baseline metastases are consistent with, but does not by itself establish, an antitumor immune effect; comparable imaging-surveillance frequency between groups argues against differential detection; death is, however, a competing event for metastasis coding, and because we did not perform a formal competing-risk analysis this association is cause-specific rather than a subdistribution estimate. Path B (disease trajectory) showed consistently favorable results including significantly reduced hospitalization, palliative referral, and opioid escalation, reflecting both potential tumor control and better depression management. Path C (BZD-specific harm) showed significantly less frequent delirium and sepsis, consistent with known BZD-associated central nervous system (CNS) toxicity [27]. All 5 individual SSRIs were associated with significantly lower mortality, supporting a class-wide SERT inhibition mechanism rather than agent-specific off-target effects.

The TCGA analysis addresses whether the inverse association between SLC6A4 expression and tumor immune-cell infiltration is tissue-specific, a question with direct clinical relevance for predicting organ-selective irAE. Across 20 solid tumor types, SLC6A4 expression was inversely correlated with T-cell inflammation in the majority of cancers and associated with the Immune Desert phenotype, a pan-cancer pattern rather than a single-tumor observation. Critically, organ-level cross-validation showed that the thyroid, where the inverse SLC6A4-immune association was strongest, was also the organ with a significant clinical irAE increase (HR 1.199; 95% CI [1.053, 1.366]; *p* = 0.006), while tissues with no SLC6A4-immune association (liver, lung) showed no irAE increase. Colon showed a comparable transcriptomic correlation (rho −0.326; FDR < 0.001) without a significant clinical increase (HR 1.130; 95% CI [0.913, 1.397]; *p* = 0.260). SLC6A4 was not independently prognostic in the pre-immunotherapy TCGA cohort, reinforcing that this axis becomes clinically relevant only in the context of checkpoint blockade.

These transcriptomic findings are best read as an independent, mechanism-oriented line of evidence rather than a demonstration that SSRIs cause the clinical associations: the TCGA samples are not from patients treated with SSRIs, so they support biological plausibility, not causation. Whether SSRI exposure actually shifts an immune-desert tumor toward an inflamed phenotype cannot be answered with cross-sectional data and would require a prospective cohort treated with ICIs with or without an SSRI, with paired pre- and post-treatment sampling and peripheral immune biomarkers, to establish such a conversion directly.

This study has several limitations. First, unmeasured confounders may introduce residual bias despite 49 propensity score-matched covariates. ECOG performance status, the most concerning potential confounder, is not captured in the TriNetX database; if SSRI users had systematically better functional status, this could partly explain the survival difference. PD-L1 expression, tumor mutational burden, and microsatellite instability status were similarly unavailable. Established pharmacologic modifiers of ICI efficacy, notably baseline systemic corticosteroids [28] and antibiotic exposure [29], were addressed by excluding baseline systemic corticosteroid users and matching on baseline medication classes, although residual effects cannot be excluded. However, the *E*-value of 2.57 indicates that an unmeasured confounder would need a strong association with both exposure and outcome to fully explain the result, and the 3 null negative control outcomes [30] argue against systematic residual confounding. Second, ICD-10-based irAE identification lacks specificity for immune-mediated etiology [31–33]; thyroid events may include non-ICI-related hypothyroidism, and neuropathy codes capture both immune-mediated and chemotherapy-induced neuropathy. Nonetheless, this misclassification would bias irAE results toward the null, making the observed thyroid signal conservative. Third, the two drug classes are prescribed in different clinical circumstances: benzodiazepines are frequently given as needed for acute anxiety, insomnia, or distress around disease progression, so the comparator group may carry a worse underlying trajectory, a form of confounding by indication that would favor SSRIs. Several findings argue against this fully explaining the result, including preservation of the association across the prespecified sensitivity analyses, an *E*-value of 2.57, and an association that persisted against a comparator receiving neither agent (HR 0.753; 95% CI [0.605, 0.939]); the per-protocol estimate was directionally stronger (HR 0.575; 95% CI [0.472, 0.702]) but, being susceptible to informative censoring that cannot be corrected on this federated platform, is treated only as a sensitivity analysis; residual differences in psychiatric severity or care intensity nonetheless cannot be excluded, and the neither-agent comparison may itself be biased if untreated patients have milder illness or lower healthcare engagement. Fourth, SSRI prescription may proxy for integrated psycho-oncology care or more comprehensive healthcare engagement rather than a direct pharmacologic effect; however, the consistent association across all 5 individual SSRIs and the organ-selective irAE pattern are difficult to explain by differential care engagement alone. Fifth, the TCGA analysis uses bulk RNA-seq data that cannot resolve cell-type-specific SLC6A4 expression on tumor-infiltrating lymphocytes, and the organ-level cross-validation involves only 5 data points, limiting formal statistical inference.

In conclusion, SSRI use was associated with lower mortality, organ-selective immune activation, and a more favorable disease trajectory compared with BZD use among patients with psychiatric comorbidity who were receiving ICIs. Transcriptomic evidence that SLC6A4 expression is inversely associated with tumor immune-cell infiltration across human cancers provides biological plausibility. These findings support prospective evaluation of SSRI repurposing as an ICI adjuvant in a randomized trial, with immune-related adverse events and peripheral immune biomarkers as co-primary end points to confirm the proposed mechanism.

### Ethics approval

This study was approved by the Institutional Review Board of Tri-Service General Hospital, National Defense Medical Center (TSGHIRB No. E202516013; certificate of review exemption issued March 10, 2025). Informed consent was waived due to the use of de-identified data. The bioinformatics analysis was approved by the Institutional Review Board of Changhua Christian Hospital (CCH IRB No. 260601). Because the analysis used only publicly available, de-identified datasets, the requirement for documentation of informed consent was waived.

## Supporting information

S1 Text Detailed study methodology. (DOCX)

S1 Protocol Pre-specified analysis plan. (DOCX)

S1 Checklist STROBE statement. The STROBE checklist is best used in conjunction with this article (freely available on the Web sites of PLoS Medicine at http://www.plosmedicine.org/, Annals of Internal Medicine at http://www.annals.org/, and Epidemiology at http://www.epidem.com/). Information on the STROBE Initiative is available at www.strobe-statement.org.
(DOCX)

S2 Checklist The TARGET Checklist. Cashin AG, Hansford HJ, Hernán MA, Swanson SA, Lee H, Jones MD, et al. Transparent reporting of observational studies emulating a target trial: The TARGET statement. JAMA. 2025; https://doi.org/10.1001/jama.2025.13350. 2025 Cashin and colleagues. This is an Open Access article distributed under the terms of the Creative Commons Attribution-NoDerivatives License (CC BY-ND 4.0), which permits redistribution, commercial and noncommercial, provided the work is passed along unchanged and in whole, with credit to the original author(s).
(DOCX)

S1 Fig Subgroup analysis: mortality hazard ratio. Forest plot of hazard ratios with 95% CIs for SSRI versus BZD across prespecified subgroups including age, sex, race, cancer type, metastatic status, psychiatric diagnosis type, ICI class, BMI, and individual SSRI agents (fluvoxamine omitted from the individual-agent panel owing to fewer than 10 users). The association with lower mortality was consistent across most subgroups. The association was strongest among patients with comorbid depression and anxiety (HR 0.57; 95% CI [0.49, 0.68]). All 5 individually analyzable SSRIs were associated with significantly lower mortality (HR range 0.61 to 0.71). Head and neck cancer was the only subgroup in which the estimate did not favor SSRI use (HR 1.08; 95% CI [0.69, 1.68]; *p* = 0.736). Hazard ratios are from Cox proportional hazards models fitted within each subgroup; *p* values are from log-rank tests.
(TIF)

S2 Fig Restricted mean survival time analysis. a, Kaplan–Meier survival curves for SSRI (blue) versus BZD (orange) with restricted mean survival time (RMST) areas shaded. At 24 months, SSRI RMST = 19.1 months versus BZD RMST = 17.0 months (delta = 2.1 months). b, Delta-RMST (SSRI minus BZD) as a function of restriction time. The difference in restricted mean survival time increases monotonically: 0.2 months at 6 months, 0.7 months at 12 months, 1.4 months at 18 months, and 2.1 months at 24 months. The 95% CI remains entirely above zero from approximately 4 months onward. RMST analysis provides a model-free estimate that does not rely on the proportional hazards assumption. It is shown here for overall survival, which met the assumption (Schoenfeld *p* = 0.825). Two secondary outcomes did violate it (palliative care *p* = 0.032; venous thromboembolism *p* = 0.042; see S5 Table).
(TIF)

S3 Fig TCGA pan-cancer survival forest plot: SLC6A4 low versus high. Random-effects meta-analysis of overall survival by SLC6A4 expression (per-cancer median split; hazard ratios expressed for low versus high) across 18 TCGA solid tumor types. Pooled HR = 1.04 (95% CI [0.94, 1.15]; *p* = 0.42; I^2^ = 13.0%). SLC6A4 expression was not independently prognostic, consistent with TCGA representing a pre-immunotherapy era cohort where the immune desert phenotype driven by SLC6A4 would not directly affect survival in the absence of immune checkpoint inhibitors. PAAD (HR 1.70; 95% CI [1.12, 2.57]; *p* = 0.011) was the only individually significant cancer type; higher SLC6A4 was associated with better survival. Per-cancer hazard ratios and *p* values are from Cox proportional hazards models; the pooled estimate, its *p* value, and I^2^ are from a random-effects meta-analysis (restricted maximum likelihood, Hartung–Knapp confidence interval); the heterogeneity *p* value printed in the figure is from Cochran Q.
(TIF)

S4 Fig PAAD kaplan–meier survival by SLC6A4 expression. Kaplan–Meier overall survival curves for pancreatic adenocarcinoma (PAAD) stratified by SLC6A4 expression (High versus Low, median split). SLC6A4-low was associated with significantly worse overall survival (hazard ratio for low versus high 1.70; 95% CI [1.12, 2.57]; log-rank *p* = 0.011; *n* = 178; events = 93); equivalently, higher SLC6A4 expression was associated with better survival in this cancer type, the largest individual-cancer association observed.
(TIF)

S5 Fig Serotonin-pathway genes versus immune signatures across TCGA cancers. Median Spearman correlation of each of 20 serotonin-pathway genes (the transporter SLC6A4, 14 serotonin receptors, the synthesis enzymes TPH1/TPH2/DDC, and the degradation enzymes MAOA/MAOB) with three immune signatures (T-cell-inflamed GEP, IFN-gamma, and cytolytic score) across 20 TCGA solid-tumor types (1,200 correlations (20 genes × 3 immune signatures × 20 cancers; pooled to 60 gene-by-signature tests in the main text); Benjamini–Hochberg corrected). SLC6A4 ranked first of 20 by pooled correlation (median rho −0.16; significant-negative in 37 of 60 gene-by-signature tests; significant-positive in only 2). The degradation enzyme MAOB correlated positively and most receptors showed weak or positive associations, indicating that the inverse association with immune signatures tracks specifically with the serotonin-handling transporter rather than the pathway at large. Blue = negative correlation; red = positive. Correlations are Spearman rank correlations; significance is assessed after Benjamini–Hochberg false discovery rate correction.
(TIF)

S6 Fig Overall survival by ICI class: Forest plot. Forest plot of overall-survival hazard ratios (SSRI versus benzodiazepine) within each ICI-class subcohort (anti-PD-1, anti-PD-L1, anti-CTLA-4, combination), with random-effects pooled estimate and heterogeneity statistics (Cochran Q test *p* = 0.75; I^2^ = 0%). Class-specific hazard ratios are from Cox proportional hazards models; the class-specific *p* values are from log-rank tests. See S16 Table. Anti-CTLA-4 and combination subcohorts are underpowered. Because the ICI-class subcohorts are not mutually exclusive, the pooled estimate and I^2^ statistic are descriptive rather than a formal test of consistency.
(TIF)

S1 Table Per-protocol sensitivity analysis: Clinical outcomes. (DOCX)

S2 Table US-only sensitivity analysis: Clinical outcomes. (DOCX)

S3 Table Dual SSRI+BZD versus BZD-only sensitivity analysis. (DOCX)

S4 Table Individual organ-system immune-related adverse events. (DOCX)

S5 Table Complete clinical outcomes: All 20 prespecified endpoints, with event counts. (DOCX)

S6 Table *E*-value analysis for unmeasured confounding (all outcomes). (DOCX)

S7 Table Propensity score matching covariates. (DOCX)

S8 Table Immune checkpoint inhibitor drug codes. (DOCX)

S9 Table SSRI and benzodiazepine drug codes. (DOCX)

S10 Table Immune-related adverse event ICD-10 code definitions. (DOCX)

S11 Table Outcome definitions and ICD-10 codes. (DOCX)

S12 Table TCGA per-cancer survival analysis: SLC6A4 low versus high. (DOCX)

S13 Table Gene set definitions used in the TCGA analyses. (DOCX)

S14 Table Organ-level cross-validation: Mapping clinical irAE to TCGA cancer types. (DOCX)

S15 Table SSRI versus neither SSRI nor benzodiazepine. (DOCX)

S16 Table Overall survival by ICI class. (DOCX)

S17 Table Imaging-surveillance frequency and supplementary negative controls. (DOCX)

S18 Table Overall survival without time-window restriction. (DOCX)

S19 Table Overall survival excluding post-baseline crossover. (DOCX)

S20 Table Complete baseline characteristics and covariate balance (all covariates). (DOCX)

S21 Table Hazard ratios before and after propensity score matching (all comparisons and outcomes). (DOCX)

## Abbreviations

BZD: benzodiazepine
CIs: confidence intervals
CNS: central nervous system
ECOG: Eastern Cooperative Oncology Group
EHR: electronic health record
FDR: false discovery rate
GABA: gamma-aminobutyric acid
GEP: Gene Expression Profile
HRs: hazard ratios
ICIs: Immune checkpoint inhibitors
irAE: immune-related adverse event
MAPK: mitogen-activated protein kinase
NK: natural killer
PAAD: pancreatic adenocarcinoma
PD-1: programmed cell death protein 1
PD-L1: programmed death ligand 1
RECIST: Response Evaluation Criteria in Solid Tumors
RMST: restricted mean survival time
SERT: serotonin transporter
SMDs: standardized mean differences
ssGSEA: Single-sample gene set enrichment analysis
SSRIs: selective serotonin reuptake inhibitors
TCGA: The Cancer Genome Atlas
TTNT: Time to next treatment.

## References

[1] Ribas A, Wolchok JD. Cancer immunotherapy using checkpoint blockade. Science. 2018;359(6382):1350–5. doi: 10.1126/science.aar4060 29567705 PMC7391259

[2] Mitchell AJ, Ferguson DW, Gill J, Paul J, Symonds P. Depression and anxiety in long-term cancer survivors compared with spouses and healthy controls: A systematic review and meta-analysis. Lancet Oncol. 2013;14(8):721–32. doi: 10.1016/S1470-2045(13)70244-4 23759376

[3] Satin JR, Linden W, Phillips MJ. Depression as a predictor of disease progression and mortality in cancer patients: A meta-analysis. Cancer. 2009;115(22):5349–61. doi: 10.1002/cncr.24561 19753617

[4] Miller AH, Raison CL. The role of inflammation in depression: From evolutionary imperative to modern treatment target. Nat Rev Immunol. 2016;16(1):22–34. doi: 10.1038/nri.2015.5 26711676 PMC5542678

[5] Herr N, Bode C, Duerschmied D. The effects of serotonin in immune cells. Front Cardiovasc Med. 2017;4:48. doi: 10.3389/fcvm.2017.00048 28775986 PMC5517399

[6] Li B, Elsten-Brown J, Li M, Zhu E, Li Z, Chen Y, et al. Serotonin transporter inhibits antitumor immunity through regulating the intratumoral serotonin axis. Cell. 2025;188(14):3823-3842.e21. doi: 10.1016/j.cell.2025.04.032 40403728 PMC12255530

[7] Schneider MA, Heeb L, Beffinger MM, Pantelyushin S, Linecker M, Roth L, et al. Attenuation of peripheral serotonin inhibits tumor growth and enhances immune checkpoint blockade therapy in murine tumor models. Sci Transl Med. 2021;13(611):eabc8188. doi: 10.1126/scitranslmed.abc8188 34524861

[8] Alvarez-Valadez K, Kroemer G, Djavaheri-Mergny M. Selective serotonin reuptake inhibitors enhance cancer immunosurveillance by pleiotropic mechanisms. Oncoimmunology. 2025;14(1):2564734. doi: 10.1080/2162402X.2025.2564734 41029175 PMC12489990

[9] Dong F, Zhang S, Song K, Jiang L, Hu LP, Li Q. Citalopram exhibits immune-dependent anti-tumor effects by modulating C5aR1 TAMs. eLife. 2026;14:RP103016. doi: 10.7554/eLife.103016 PMC12885477 41661166

[10] Magagnoli J, Narendran S, Pereira F, Cummings TH, Hardin JW, Sutton SS, et al. Association between fluoxetine use and overall survival among patients with cancer treated with PD-1/L1 immunotherapy. Pharmaceuticals (Basel). 2023;16(5):640. doi: 10.3390/ph16050640 37242422 PMC10220705

[11] Derry-Vick H, Shah NJ, Ahn J, DeAgresta B, Della Pia A, Zaemes JP, et al. Associations between anxiety or depression diagnosis and immune checkpoint inhibitor outcomes. Cancer Med. 2025;14(21):e71326. doi: 10.1002/cam4.71326 41176702 PMC12579804

[12] Zeng Y, Hu C-H, Li Y-Z, Zhou J-S, Wang S-X, Liu M-D, et al. Association between pretreatment emotional distress and immune checkpoint inhibitor response in non-small-cell lung cancer. Nat Med. 2024;30(6):1680–8. doi: 10.1038/s41591-024-02929-4 38740994 PMC11186781

[13] Hernán MA, Robins JM. Using big data to emulate a target trial when a randomized trial is not available. Am J Epidemiol. 2016;183(8):758–64. doi: 10.1093/aje/kwv254 26994063 PMC4832051

[14] Lund JL, Richardson DB, Stürmer T. The active comparator, new user study design in pharmacoepidemiology: Historical foundations and contemporary application. Curr Epidemiol Rep. 2015;2(4):221–8. doi: 10.1007/s40471-015-0053-5 26954351 PMC4778958

[15] Kyriacou DN, Lewis RJ. Confounding by indication in clinical research. JAMA. 2016;316(17):1818–9. doi: 10.1001/jama.2016.16435 27802529

[16] von Elm E, Altman DG, Egger M, Pocock SJ, Gøtzsche PC, Vandenbroucke JP, et al. The Strengthening the Reporting of Observational Studies in Epidemiology (STROBE) statement: Guidelines for reporting observational studies. Lancet. 2007;370(9596):1453–7. doi: 10.1016/S0140-6736(07)61602-X 18064739

[17] Cashin AG, Hansford HJ, Hernán MA, Swanson SA, Lee H, Jones MD, et al. Transparent reporting of observational studies emulating a target trial-the TARGET statement. JAMA. 2025;334(12):1084–93. doi: 10.1001/jama.2025.13350 40899949 PMC13084563

[18] Austin PC. An introduction to propensity score methods for reducing the effects of confounding in observational studies. Multivariate Behav Res. 2011;46(3):399–424. doi: 10.1080/00273171.2011.568786 21818162 PMC3144483

[19] Suissa S. Immortal time bias in pharmaco-epidemiology. Am J Epidemiol. 2008;167(4):492–9. doi: 10.1093/aje/kwm324 18056625

[20] VanderWeele TJ, Ding P. Sensitivity analysis in observational research: Introducing the E-value. Ann Intern Med. 2017;167(4):268–74. doi: 10.7326/M16-2607 28693043

[21] Barbie DA, Tamayo P, Boehm JS, Kim SY, Moody SE, Dunn IF, et al. Systematic RNA interference reveals that oncogenic KRAS-driven cancers require TBK1. Nature. 2009;462(7269):108–12. doi: 10.1038/nature08460 19847166 PMC2783335

[22] Ayers M, Lunceford J, Nebozhyn M, Murphy E, Loboda A, Kaufman DR, et al. IFN-γ-related mRNA profile predicts clinical response to PD-1 blockade. J Clin Invest. 2017;127(8):2930–40. doi: 10.1172/JCI91190 28650338 PMC5531419

[23] Rooney MS, Shukla SA, Wu CJ, Getz G, Hacohen N. Molecular and genetic properties of tumors associated with local immune cytolytic activity. Cell. 2015;160(1–2):48–61. doi: 10.1016/j.cell.2014.12.033 25594174 PMC4856474

[24] Mariathasan S, Turley SJ, Nickles D, Castiglioni A, Yuen K, Wang Y, et al. TGFβ attenuates tumour response to PD-L1 blockade by contributing to exclusion of T cells. Nature. 2018;554(7693):544–8. doi: 10.1038/nature25501 29443960 PMC6028240

[25] Kotwal A, Kottschade L, Ryder M. PD-L1 inhibitor-induced thyroiditis is associated with better overall survival in cancer patients. Thyroid. 2020;30(2):177–84. doi: 10.1089/thy.2019.0250 31813343 PMC7047075

[26] Bauer M, Goetz T, Glenn T, Whybrow PC. The thyroid-brain interaction in thyroid disorders and mood disorders. J Neuroendocrinol. 2008;20(10):1101–14. doi: 10.1111/j.1365-2826.2008.01774.x 18673409

[27] Zavala F. Benzodiazepines, anxiety and immunity. Pharmacol Ther. 1997;75(3):199–216. doi: 10.1016/s0163-7258(97)00055-7 9504140

[28] Arbour KC, Mezquita L, Long N, Rizvi H, Auclin E, Ni A, et al. Impact of baseline steroids on efficacy of programmed cell death-1 and programmed death-ligand 1 blockade in patients with non-small-cell lung cancer. J Clin Oncol. 2018;36(28):2872–8. doi: 10.1200/JCO.2018.79.0006 30125216

[29] Pinato DJ, Howlett S, Ottaviani D, Urus H, Patel A, Mineo T, et al. Association of prior antibiotic treatment with survival and response to immune checkpoint inhibitor therapy in patients with cancer. JAMA Oncol. 2019;5(12):1774–8. doi: 10.1001/jamaoncol.2019.2785 31513236 PMC6743060

[30] Lipsitch M, Tchetgen Tchetgen E, Cohen T. Negative controls: A tool for detecting confounding and bias in observational studies. Epidemiology. 2010;21(3):383–8. doi: 10.1097/EDE.0b013e3181d61eeb 20335814 PMC3053408

[31] Wang F, Yang S, Palmer N, Fox K, Kohane IS, Liao KP, et al. Real-world data analyses unveiled the immune-related adverse effects of immune checkpoint inhibitors across cancer types. NPJ Precis Oncol. 2021;5(1):82. doi: 10.1038/s41698-021-00223-x 34508179 PMC8433190

[32] Heyward JS, Segal JB, Mehta HB, Murray JC. Validation of immune-related adverse event (irAE) case definitions in a real-world lung cancer population. Pharmacoepidemiol Drug Saf. 2025;34(2):e70100. doi: 10.1002/pds.70100 39961795 PMC11970256

[33] Cheung Y-MM, Hamnvik O-PR, Shariff A, Gallagher EJ. Dearth of ICD codes for complications of immune checkpoint inhibitors impedes clinical care and research. J Endocr Soc. 2023;7(4):bvad019. doi: 10.1210/jendso/bvad019 36819460 PMC9933895

