# Supplementary material for: Selective serotonin reuptake inhibitors and immune checkpoint blockade in solid tumors: A target trial emulation with pan-cancer transcriptomic analyses
Source: PLoS Med. 2026 Sep 29;23(9):e1005262. doi: 10.1371/journal.pmed.1005262 (PMC13645231; doi:10.1371/journal.pmed.1005262)
Supplement: Supplementary file 1 [file pmed.1005262.s001.docx]

## **S1 Text. Detailed Study Methodology**

## Study Design and Data Source

This retrospective cohort study utilized the TriNetX Global Collaborative Network, a federated health research network encompassing electronic health records from over 187 million patients across 172 healthcare organizations. The study employed an active-comparator design comparing SSRI users versus benzodiazepine (BZD) users among patients with a solid tumor and pre-existing depression or anxiety who received immune checkpoint inhibitor (ICI) therapy. The study period spanned January 2015 to December 2025. This design directly tests the clinical translation of Li et al. (*Cell* 2025) preclinical findings demonstrating SSRI–ICI synergy through serotonin transporter (SERT) inhibition.

## Inclusion Criteria

Patients were included if they met all of the following: (1) Age ≥18 years at index date; (2) Diagnosis of solid malignant neoplasm (ICD-10-CM: C00–C80) documented prior to or on index date; (3) First administration of any of the 13 FDA-approved ICI agents listed in S8 Table between January 1, 2015 and December 31, 2025, defining the index date; (4) Diagnosis of major depressive disorder (F32.x, F33.x), generalized anxiety disorder (F41.1), or anxiety disorder NOS (F41.9) within 365 days before index date; (5) Active prescription of SSRI or BZD within 90 days before to 14 days after index date; (6) Minimum 365 days of EHR data before index date; (7) Minimum 1 day of follow-up after index date.

## Exclusion Criteria

Patients were excluded if they had: (1) Hematologic malignancy (C81–C96) at any time before index; (2) Pre-existing autoimmune disease requiring immunosuppressive therapy; (3) HIV (B20) at any time; (4) History of solid organ or hematopoietic stem cell transplant (Z94); (5) Schizophrenia spectrum (F20–F29) or bipolar disorder (F30–F31) at any time; (6) Systemic corticosteroid ≥10 mg prednisone equivalent/day for ≥14 days within 30 days before index; (7) Delirium (F05) within 30 days before index; (8) Palliative care (Z51.5) within 30 days before index; (9) Concurrent prescription of both SSRI and BZD overlapping index date (primary analysis); (10) Any non-SSRI antidepressant (SNRI, TCA, MAOI) prescription overlapping index date.

## Rationale for Active Comparator Design

Comparing SSRI users to BZD users (rather than untreated or general population) reduces confounding by indication, as both groups have psychiatric comorbidity and are receiving psychotropic treatment. The mechanistic contrast is serotonergic (SSRI: SERT inhibition, intratumoral serotonin accumulation, CD8+ T-cell enhancement) versus GABAergic (BZD: GABA-A agonist, no known immune-enhancing effect, possible mild immunosuppression via TSPO on lymphocytes).

## Follow-up and Censoring

Follow-up began on day 30 after the index date (first ICI administration), a prespecified 30-day landmark, and continued through day 730 (primary analysis window, day 30–730). Overall survival was assessed with a 2-year primary window. Patients alive at last follow-up were censored.

## Propensity Score Matching Methodology

Propensity scores were estimated using logistic regression with baseline covariates measured during the 365-day period before index date (see S7 Table for complete list). One-to-one nearest-neighbor matching was performed using a greedy algorithm with a caliper of 0.1 standard deviations of the logit propensity score. Covariate balance was assessed using standardized mean differences (SMD), with SMD <0.10 indicating adequate balance. Critical variables (cancer site, metastatic status, ICI agent, psychiatric diagnosis type) targeted SMD <0.05. The primary analysis yielded 1,567 matched pairs from 1,661 SSRI users and 5,500 BZD users before matching.

## Primary and Secondary Outcomes

The primary outcome was overall survival (OS): time from index date to death from any cause. Key secondary outcomes included composite immune-related adverse events (irAE) using narrow (10 organ systems) and broad (13 organ systems) definitions. All 20 secondary outcomes were organized by 4 prespecified mechanistic pathways to discriminate between competing explanations: Path A (SSRI immune enhancement/tumor control), Path B (better mental health/treatment adherence), Path C (BZD-specific harm), and Path D (negative control outcomes). See S11 Table for complete outcome definitions.

## Mechanistic Pathway Framework

The observed mortality association could arise from multiple mechanisms. **Path A (SSRI Immune Enhancement):** If SSRIs enhance antitumor immunity via SERT inhibition, we expect more irAE, fewer new metastases, and later treatment change. **Path B (Better Disease Trajectory):** If the benefit reflects better depression/anxiety control and health care engagement, we expect fewer ED visits, less hospitalization, and later palliative referral. **Path C (BZD-Specific Harm):** If BZD-specific adverse effects contribute, we expect more falls/fractures, delirium, infections, and VTE in the BZD group. **Path D (Negative Controls):** Outcomes with no biological link to SSRI, BZD, or immunity (kidney stone, gallstone, cataract) should show HR ≈ 1.0; significant deviation indicates residual confounding.

## Sensitivity Analyses

Three sensitivity analyses were performed. (1) Per-protocol analysis: restricted to patients with continuous medication exposure throughout observation (N = 695 matched pairs), addressing non-adherence bias. (2) US-only analysis: restricted to US healthcare organizations only (N = 1,450 matched pairs), ensuring regulatory and practice pattern homogeneity. (3) Dual SSRI+BZD vs BZD-only: patients concurrently using both medications compared with BZD-only users (N = 2,457 matched pairs), testing whether concurrent BZD attenuates the SSRI mortality association.

## E-Value Calculation

E-values were calculated following VanderWeele and Ding (2017) to assess robustness to unmeasured confounding. For hazard ratios >1, the E-value formula is: E-value = HR + √(HR × (HR − 1)). For hazard ratios <1 (protective effect), HR is first inverted (1/HR) before applying the same formula. The E-value represents the minimum strength of association (on the RR scale) that an unmeasured confounder would need with both the exposure (SSRI vs BZD assignment) and the outcome to fully explain away the observed association. The 95% CI E-value uses the confidence interval bound closest to the null.

## Negative Control Analysis

Three negative control outcomes (kidney stone [N20], gallbladder stone [K80], and cataract [H25–H26]) were selected as conditions without biological plausibility for differential association between SSRI and BZD use. Significant associations would suggest residual unmeasured confounding or systematic bias, requiring cautious interpretation of all findings.

## Statistical Analysis

The primary analysis used Cox proportional hazards regression with time-to-event as the primary endpoint. Kaplan–Meier survival curves with log-rank tests were generated. Proportional hazards assumptions were tested using Schoenfeld residuals. Restricted mean survival time (RMST) is reported for the primary outcome (see S2 Fig). Palliative care (p=0.032) and VTE (p=0.042) showed evidence of violation; RMST is not reported for these two outcomes and their hazard ratios should be read as period-averaged effects. Risk ratios with 95% CI were calculated for binary outcomes. No formal adjustment for multiple comparisons was applied; the primary outcome was prespecified, and the secondary outcomes are reported as hypothesis-generating with p-values interpreted descriptively. All results are reported with 95% confidence intervals and exact p-values.

## **S1 Text (continued). TCGA Pan-Cancer Transcriptomic Analyses**

To investigate the biological basis of the observed clinical association, we examined the association between SLC6A4 expression and immune microenvironment features across 20 solid tumor types from The Cancer Genome Atlas (TCGA). RNA-seq count data and clinical annotations were obtained from the GDC Data Portal via TCGAbiolinks-curated RDS objects. For skin cutaneous melanoma (SKCM), metastatic samples (n = 368) were used to align with the immunotherapy-relevant population; for all other cancer types, primary tumor samples were used. In total, 8,272 tumor samples contributed by 8,172 patients were analyzed; 100 patients contributed more than one tumor sample, and no patient appeared in more than one tumor type.

Single-sample gene set enrichment analysis (ssGSEA). Gene set enrichment scores were computed using the GSVA package (v1.50) with the ssGSEA method. Eleven gene sets were defined for two analytical purposes (S13 Table): (1) immune function assessment (T-cell Inflamed GEP, Cytotoxic Score, Serotonin Axis, and a 6-gene interferon-gamma signature) and (2) immune phenotype classification and cell-type deconvolution (Excluded Stroma, CD8+ T, CD4+ T, Treg, NK, M2 Macrophage, and CAF signatures).

SLC6A4-immune correlation analysis (Figure 5A). Spearman rank correlations between SLC6A4 ssGSEA score and each immune function score were computed per cancer type. p-values were adjusted for multiple comparisons using the Benjamini-Hochberg procedure across all 20 cancer types within each score.

Immune phenotype classification (Figure 5B). Tumors were classified into three immune phenotypes based on per-cancer median ssGSEA scores: Inflamed (T-cell Inflamed GEP above median and Excluded Stroma at or below median), Excluded (Excluded Stroma above median, regardless of T-cell score), and Desert (both T-cell Inflamed GEP and Excluded Stroma at or below median). The proportion of SLC6A4-high samples (above per-cancer median SLC6A4 expression) within each phenotype was compared using the Kruskal-Wallis test, with Benjamini-Hochberg FDR correction.

Immune cell composition analysis (Figure 5C). Spearman correlations between SLC6A4 expression and six cell-type ssGSEA scores (CD8+ T, CD4+ T, NK, M2 Macrophage, Treg, and CAF) were computed per cancer type. Only values with FDR < 0.05 are displayed.

Organ-level cross-validation (Figure 5D). To assess whether the tissue-level SLC6A4-immune association corresponds to the organ-specific pattern of clinical irAE enhancement, we mapped clinical organ-specific irAE hazard ratios to TCGA cancer-type SLC6A4-immune correlation coefficients for five organ systems with direct anatomical correspondence (S14 Table). Neuropathy was excluded as it primarily reflects chemotherapy-induced peripheral neuropathy. For lung, LUAD was selected over LUSC as it better represents peripheral parenchymal tissue affected by pneumonitis.

Survival analysis (S3 Fig). Cox proportional hazards regression was performed per cancer type using median-split SLC6A4 expression (High vs Low). A random-effects meta-analysis (REML estimator, Hartung-Knapp confidence interval) pooled hazard ratios across 18 evaluable cancer types (minimum n = 30 or events >= 10).

Software. All analyses were performed in R (v4.3+) using GSVA, survival, survminer, meta, and tidyverse packages.
