# Supplementary material for: Selective serotonin reuptake inhibitors and immune checkpoint blockade in solid tumors: A target trial emulation with pan-cancer transcriptomic analyses
Source: PLoS Med. 2026 Sep 29;23(9):e1005262. doi: 10.1371/journal.pmed.1005262 (PMC13645231; doi:10.1371/journal.pmed.1005262)
Supplement: Supplementary file 2 [file pmed.1005262.s002.docx]

**S1 Protocol. Pre-specified analysis plan.**

**PRE-SPECIFIED ANALYSIS PLAN**

**Serotonin Transporter Inhibition and Immune Checkpoint Blockade Outcomes:**

**Target Trial Emulation With Pan-Cancer Transcriptomic Validation**

**Principal Investigator: Cho-Hao Lee, MD**

First Author: Po-Huang Chen, MD

Division of Hematology and Oncology, Department of Internal Medicine

Tri-Service General Hospital, National Defense Medical University, Taipei, Taiwan

**IRB Approval: TSGHIRB No. E202516013**

Date of IRB Exemption: March 10, 2025

Version: 1.0 (February 2025); Amendment 1 (June 2026)

# 1. Background and Rationale

A landmark preclinical study by Li et al (Cell, 2025) identified the serotonin transporter (SERT, encoded by SLC6A4) as an immune checkpoint on CD8+ T cells. SERT-mediated serotonin reuptake suppressed T-cell receptor signaling via the 5-HTR–MAPK–ERK pathway, and selective serotonin reuptake inhibitors (SSRIs) reversed this suppression and synergized with anti-PD-1 therapy in murine models. These findings were independently supported by studies demonstrating enhanced immunosurveillance through serotonergic mechanisms, including peripheral serotonin attenuation, pleiotropic SSRI-mediated immune enhancement, and tumor-associated macrophage modulation.

However, clinical evidence for this SSRI-ICI interaction remains limited. Prior retrospective studies were constrained by small samples, single-agent designs, absence of active comparators, and lack of transcriptomic validation. This study aims to provide large-scale clinical evidence for the SSRI-ICI synergy hypothesis using a target trial emulation framework, complemented by pan-cancer transcriptomic validation using The Cancer Genome Atlas (TCGA).

# 2. Study Objectives

## 2.1 Primary Objective

To compare 2-year overall survival between SSRI users and benzodiazepine (BZD) users among patients with a solid tumor and depression or anxiety who were receiving an immune checkpoint inhibitor (ICI), using a target trial emulation design with an active-comparator, new-user framework.

## 2.2 Secondary Objectives

1. To evaluate 20 secondary outcomes organized across 4 prespecified mechanistic pathways (immune enhancement, disease trajectory, BZD-specific harm, negative controls).
2. To assess the association between SLC6A4 expression and immune microenvironment features across 20 TCGA solid tumor types.
3. To perform organ-level cross-validation mapping clinical irAE patterns to transcriptomic SLC6A4-immune correlations.

# 3. Target Trial Specification

The study follows the target trial emulation framework as described by Hernán and Robins (Am J Epidemiol, 2016). The seven components of the target trial and their emulation are specified below:

| **Component** | **Target Trial** | **Emulation** |
| --- | --- | --- |
| **Eligibility criteria** | Adults (≥18 y) with solid tumor (ICD-10-CM C00–C80), first ICI, documented MDD (F32–F33) or anxiety disorder (F41.1, F41.9) within 365 days before ICI, and SSRI or BZD prescription within 90 days before to 14 days after ICI initiation. | Same criteria applied to TriNetX Global Collaborative Network (>187 million patients, 172 HCOs). 10 exclusion criteria applied sequentially (see Section 4.3). Minimum 365 days EHR data before index and minimum 1 day follow-up required. |
| **Treatment strategies** | Strategy A: Initiate SSRI (without concurrent BZD) within 90 days before to 14 days after first ICI.  Strategy B: Initiate BZD (without concurrent antidepressants) within same window. | Exposure classified by prescription records for 6 SSRIs (fluoxetine, sertraline, paroxetine, citalopram, escitalopram, or fluvoxamine) vs 7 BZDs (alprazolam, lorazepam, clonazepam, diazepam, temazepam, oxazepam, or chlordiazepoxide). See S9 Table for drug codes. |
| **Treatment assignment** | Random assignment to SSRI or BZD at time of ICI initiation. | 1:1 propensity score matching (greedy nearest-neighbor, caliper 0.1 SD of logit PS) with 49 covariates: demographics, tumor characteristics, psychiatric diagnosis type, 12 medical comorbidities, 11 baseline medications, 11 laboratory values, and 3 baseline treatment and clinical variables (chemotherapy, radiotherapy, and body-mass index) (see S7 Table). |
| **Follow-up period** | Begins at the prespecified 30-day landmark (day 30 after first ICI). Follow-up continues until death, last EHR contact, or day 730, whichever comes first. | Same structure. Patients censored at last known encounter if alive. Primary OS: day 30–730 (prespecified 30-day landmark). Secondary outcome window: 30–730 days post-index for new-diagnosis endpoints. |
| **Outcomes** | Primary: Overall survival (time from first ICI to death from any cause; 2-year window). 20 secondary outcomes across 4 prespecified mechanistic pathways (see Section 6). | Same outcomes. OS ascertained from death records. Secondary outcomes via ICD-10-CM codes, restricted to new diagnoses not present in 365-day baseline. Complete definitions in S11 Table. |
| **Causal contrast** | Intention-to-treat effect: initiating SSRI vs BZD at time of ICI. | ITT analogue via Cox PH regression. Per-protocol analysis (continuous medication exposure) as sensitivity analysis. |
| **Statistical analysis** | ITT Cox regression. KM curves with log-rank test. Prespecified subgroup analyses. E-values for unmeasured confounding. 3 negative control outcomes. | Cox PH. Schoenfeld residuals for PH assumption. RMST for the primary outcome. E-values per VanderWeele & Ding (2017). No formal multiple-comparison adjustment (prespecified pathway framework; secondary outcomes hypothesis-generating). |

# 4. Study Population and Data Source

## 4.1 Data Source

TriNetX Global Collaborative Network: a federated electronic health record (EHR) platform integrating de-identified data from over 187 million patients across 172 healthcare organizations (academic medical centers, community hospitals, and specialty practices) spanning all 50 US states and 18 countries. The study period spans January 2015 to December 2025.

## 4.2 Inclusion Criteria

1. Age ≥18 years at index date
2. Diagnosis of solid malignant neoplasm (ICD-10-CM: C00–C80) documented prior to or on index date
3. First administration of an FDA-approved ICI agent (see S8 Table for complete list of 13 agents) between January 1, 2015 and December 31, 2025, defining the index date
4. Diagnosis of major depressive disorder (F32.x, F33.x), generalized anxiety disorder (F41.1), or anxiety disorder NOS (F41.9) within 365 days before index date
5. Active prescription of SSRI or BZD within 90 days before to 14 days after index date
6. Minimum 365 days of EHR data before index date
7. Minimum 1 day of follow-up after index date

## 4.3 Exclusion Criteria

1. Hematologic malignancy (C81–C96) at any time before index
2. Pre-existing autoimmune disease requiring immunosuppressive therapy
3. HIV (B20) at any time
4. History of solid organ or hematopoietic stem cell transplant (Z94)
5. Schizophrenia spectrum (F20–F29) or bipolar disorder (F30–F31) at any time
6. Systemic corticosteroid ≥10 mg prednisone equivalent/day for ≥14 days within 30 days before index
7. Delirium (F05) within 30 days before index
8. Palliative care (Z51.5) within 30 days before index
9. Concurrent prescription of both SSRI and BZD overlapping index date (primary analysis)
10. Any non-SSRI antidepressant (SNRI, TCA, MAOI) prescription overlapping index date

# 5. Exposure Definition

## 5.1 Treatment Group (SSRI)

SSRI prescription (fluoxetine, sertraline, paroxetine, citalopram, escitalopram, or fluvoxamine) within 90 days before to 14 days after first ICI administration, without concurrent BZD use. See S9 Table for complete drug codes.

## 5.2 Active Comparator (BZD)

BZD prescription (alprazolam, lorazepam, clonazepam, diazepam, temazepam, oxazepam, or chlordiazepoxide) within the same window, without concurrent antidepressant use. See S9 Table for complete drug codes.

## 5.3 Rationale for Active Comparator Design

Comparing SSRI to BZD (rather than untreated patients) reduces confounding by indication, as both groups have psychiatric comorbidity and are receiving psychotropic treatment. The mechanistic contrast isolates serotonergic (SSRI: SERT inhibition, intratumoral serotonin accumulation, CD8+ T-cell enhancement) vs GABAergic (BZD: GABA-A agonist, no known serotonergic immune-enhancing mechanism) pharmacology.

# 6. Outcome Definitions

## 6.1 Primary Outcome

Overall survival (OS): time from index date (first ICI administration) to death from any cause. For overall survival, follow-up was assessed from a prespecified 30-day landmark (day 30 after the index date) until death, last EHR contact, or day 730, whichever comes first.

## 6.2 Secondary Outcomes (20 Prespecified Endpoints)

All 20 secondary outcomes are organized by 4 prespecified mechanistic pathways to discriminate between competing explanations. Unless otherwise noted, outcomes are restricted to new diagnoses occurring 30–730 days after ICI initiation, not present in the 365-day baseline period. Complete ICD-10-CM code definitions are provided in S11 Table.

**Path A: SSRI Immune Enhancement / Tumor Control (6 endpoints):**

- 1. Composite irAE, narrow definition (10 organ systems)
- 2. Thyroid irAE
- 3. New systemic corticosteroid prescription (proxy for irAE treatment; excludes baseline users)
- 4. New distant metastasis (restricted denominator: patients without baseline metastatic sites)
- 5. Time to next treatment / new chemotherapy initiation (TTNT; proxy for first-line ICI duration)
- 6. ICI treatment instances (number of ICI administrations, 30–730 days)

**Path B: Better Mental Health / Treatment Adherence (6 endpoints):**

- 7. Emergency department visits
- 8. Hospitalization (unplanned inpatient admission)
- 9. Palliative or hospice care referral (Z51.5)
- 10. New psychiatric diagnosis (F32, F33, F41, F43; post-index)
- 11. Opioid dose escalation (new or stronger opioid prescription)
- 12. Suicidal ideation or self-harm (R45.851, T14.91, X71–X83)

**Path C: BZD-Specific Harm (5 endpoints):**

- 13. Falls (W00–W19, R29.6)
- 14. Fractures, traumatic or osteoporotic fragility (S12/S22/S32/S42/S52/S62/S72/S82/S92, M80)
- 15. Delirium (F05, R41.0, R41.82)
- 16. Sepsis (A41, R65.2, A49)
- 17. Venous thromboembolism (I26 [PE], I82 [DVT])

**Path D: Negative Control Outcomes (3 endpoints):**

- 18. Nephrolithiasis (N20; excludes prior stones)
- 19. Cholelithiasis (K80; excludes prior gallbladder stones)
- 20. Cataract (H25, H26; excludes prior cataract)

Note: Path D outcomes serve as bias indicators. Significant associations would suggest residual unmeasured confounding or systematic bias, requiring cautious interpretation of all findings.

# 7. Propensity Score Matching

One-to-one greedy nearest-neighbor matching without replacement, caliper of 0.1 SD of the logit propensity score. The propensity score model includes 49 covariates organized as follows (see S7 Table for complete list):

- Demographics: age, sex, race/ethnicity
- Tumor characteristics: primary cancer site, metastatic status, number of metastatic sites
- ICI agent and class (PD-1, PD-L1, CTLA-4, combination)
- Psychiatric diagnosis type (MDD vs anxiety disorder)
- 12 medical comorbidities (individual conditions)
- 11 baseline medications
- 11 laboratory values (including neutrophils and albumin)
- 3 baseline treatment and clinical variables (chemotherapy, radiotherapy, and body-mass index)

All covariates measured during the 365-day baseline period before index date. Balance threshold: standardized mean difference (SMD) <0.10 for all covariates, <0.05 for critical variables (cancer site, metastatic status, ICI agent, neutrophils, albumin, psychiatric diagnosis type).

# 8. Statistical Analysis Plan

## 8.1 Primary Analysis

Cox proportional hazards regression with time-to-event as the primary endpoint. Kaplan–Meier survival curves with log-rank tests. Hazard ratios with 95% confidence intervals. Risk ratios with 95% CI calculated for binary outcomes. All results reported with exact p-values.

## 8.2 Proportional Hazards Assessment

Schoenfeld residuals tested for all outcomes. Restricted mean survival time (RMST) analysis reported for the primary outcome.

## 8.3 Sensitivity Analyses

1. Per-protocol analysis: restricted to patients with continuous medication exposure throughout the observation period, addressing non-adherence bias.
2. US-only analysis: restricted to US healthcare organizations, ensuring regulatory and practice pattern homogeneity.
3. Dual SSRI+BZD analysis: concurrent SSRI+BZD users vs BZD-only users, testing whether concurrent BZD attenuates the SSRI mortality association (mechanistic probe).

Additionally, the primary overall-survival analysis used a prespecified 30-day landmark (day 30–730). The primary analysis window (day 30–730 for overall survival and for secondary new-diagnosis endpoints) addresses early immortal time through the 30-day landmark.

## 8.4 Robustness Assessments

- E-values calculated per VanderWeele and Ding (2017) for unmeasured confounding
- Three negative control outcomes (Path D) to detect residual systematic bias
- Prespecified subgroup analyses by age, sex, race, cancer type, metastatic status, psychiatric diagnosis, ICI class, BMI, and individual SSRI agent

## 8.5 Multiple Comparisons

No formal adjustment for multiple comparisons was applied to clinical outcomes, given the prespecified pathway framework in which overall survival is the single primary outcome and the secondary outcomes are interpreted as hypothesis-generating with descriptive p-values. Benjamini–Hochberg false discovery rate (FDR) correction applied to all TCGA analyses.

## 8.6 Transcriptomic Validation (TCGA)

RNA-seq data from 8,272 samples across 20 solid tumor types from The Cancer Genome Atlas (TCGA). Analyses include:

- SLC6A4-immune correlation analysis: Spearman correlations between SLC6A4 ssGSEA score and immune function scores (T-cell Inflamed GEP, Cytotoxic Score) per cancer type, with BH-FDR correction
- Immune phenotype classification: tumors classified as Inflamed, Excluded, or Desert based on per-cancer median ssGSEA scores; proportion of SLC6A4-high samples compared across phenotypes
- Immune cell composition: Spearman correlations between SLC6A4 and 6 cell-type ssGSEA scores (CD8+ T, CD4+ T, NK, M2 macrophage, Treg, CAF) per cancer type
- Survival meta-analysis: Cox PH per cancer type with median-split SLC6A4; random-effects meta-analysis (REML, Hartung-Knapp CI) across 18 evaluable cancer types
- Organ-level cross-validation: mapping tissue-specific SLC6A4-immune correlations to clinical organ-specific irAE hazard ratios for 5 organ systems with direct anatomical correspondence

# 9. Sample Size

This study uses the full available cohort from the TriNetX Global Collaborative Network meeting the eligibility criteria specified in Section 4. No a priori sample size calculation was performed, as the study leverages all eligible patients in a federated EHR network exceeding 187 million individuals. Sequential application of inclusion and exclusion criteria determines the final analytic cohort. One-to-one propensity score matching further defines the primary analysis population. Three prespecified sensitivity analyses (per-protocol, US-only, dual SSRI+BZD) generate separate matched cohorts with expected smaller sample sizes due to additional restrictions.

The expected yield is governed by the prevalence of patients with a solid tumor receiving ICI with concurrent psychiatric diagnoses and psychotropic prescriptions meeting the specified exposure windows. Actual sample sizes are reported in the manuscript Results and Figure 1.

# 10. Ethics and Regulatory

This study was approved by the Institutional Review Board of Tri-Service General Hospital, National Defense Medical Center (TSGHIRB No. E202516013; certificate of review exemption issued March 10, 2025). Informed consent was waived because the study uses de-identified, retrospective data from the TriNetX federated network with no direct patient contact.

This study is not a prospective clinical trial and was not registered in a clinical trial registry. The study follows the target trial emulation framework (Hernán & Robins, 2016) applied to retrospective observational data, and adheres to the STROBE reporting guideline and the TARGET checklist for target trial emulation (Cashin et al., JAMA, 2025). All analyses specified in this plan were prespecified before data extraction; the post-hoc analyses added during peer review are identified in Section 11.

# 11. Amendments to Analysis Plan

The original analysis plan (Sections 1 to 10), comprising the primary outcome, all 20 secondary outcomes, the 4-pathway mechanistic framework, the 3 prespecified sensitivity analyses (per-protocol, US-only, and dual SSRI plus benzodiazepine), and the TCGA transcriptomic validation, was specified before data extraction and was implemented as written, without amendment or deviation to those analyses. During peer review (June 2026), the analyses listed below were added in response to reviewer and editor requests. They are post-hoc with respect to this prespecified plan, are identified as such in the manuscript, and are interpreted as hypothesis-generating and supportive rather than confirmatory:

1. Overall survival without time-window restriction (Reviewer 1): hazard ratio 0.596 (95% CI [0.532, 0.667]).

2. Exclusion of patients who initiated the opposite drug class during follow-up (Reviewer 1): hazard ratio 0.668 (95% CI [0.583, 0.767]).

3. A third comparator arm of patients with depression or anxiety receiving neither an SSRI nor a benzodiazepine, matched 1:1 on the same covariates (Reviewer 2): hazard ratio 0.753 (95% CI [0.605, 0.939]).

4. Overall survival estimated within each ICI class, with between-class heterogeneity assessed by the I-squared statistic (Academic Editor and Reviewer 2). Because the ICI-class subcohorts are not mutually exclusive, the pooled estimate and heterogeneity statistics are descriptive rather than a formal test of consistency.

5. A refined time-to-next-treatment endpoint, defined as initiation of a new systemic anticancer agent not part of the baseline regimen, with continued immune checkpoint inhibitor not counted as an event and death censored (Reviewer 2): hazard ratio 0.876 (95% CI [0.811, 0.946]). The narrower prespecified chemotherapy-only definition (null, hazard ratio 1.021) is described for transparency in the response to reviewers and is not reported in the manuscript or supplement.

6. Comparison of the per-patient frequency of surveillance imaging between groups, to assess detection bias for the metastasis endpoint (Reviewer 2): hazard ratio 0.957 (95% CI [0.875, 1.047]).

7. Extension of the transcriptomic analysis to the full 20-gene serotonin pathway, ranking each gene by its correlation with immune signatures across the TCGA cancers (Editor, Results comment); SLC6A4 ranked first of 20.

These post-hoc analyses were performed on contemporaneous re-extractions of the TriNetX network and therefore involve matched-cohort sizes that differ slightly from the primary analysis; effect directions were consistent throughout.

# 12. Deviations From Analysis Plan

No deviations from the prespecified analyses occurred; all prespecified analyses were completed as specified. The additional analyses listed in Section 11 are post-hoc additions made during peer review in response to reviewer and editor requests, not deviations from the prespecified analyses.

# 13. Data Availability

The TriNetX platform operates as a federated network in which patient-level data remain within participating healthcare organizations and cannot be exported, shared, or transferred outside the platform per institutional data use agreements. Aggregate-level results and summary statistics generated through the TriNetX query interface are reported in full in the manuscript and its Supporting Information files. TCGA RNA-seq data and clinical annotations are publicly available from the Genomic Data Commons (https://portal.gdc.cancer.gov). All R analysis scripts for the TCGA transcriptomic validation are publicly available on GitHub (https://github.com/chenpohuang/ssri-ici-transcriptomics).

# 14. References

1. Li B, et al. Serotonin transporter inhibits antitumor immunity through regulating the intratumoral serotonin axis. Cell. 2025;188(14):3823-3842.
2. Hernán MA, Robins JM. Using big data to emulate a target trial when a randomized trial is not available. Am J Epidemiol. 2016;183(8):758-764.
3. VanderWeele TJ, Ding P. Sensitivity analysis in observational research: introducing the E-value. Ann Intern Med. 2017;167(4):268-274.
4. Cashin AG, et al. Transparent Reporting of Observational Studies Emulating a Target Trial: The TARGET Statement. JAMA. 2025.
5. Lund JL, et al. The active comparator, new user study design in pharmacoepidemiology. Curr Epidemiol Rep. 2015;2(4):221-228. doi:10.1007/s40471-015-0053-5.
6. von Elm E, et al. The Strengthening the Reporting of Observational Studies in Epidemiology (STROBE) Statement: guidelines for reporting observational studies. Lancet. 2007;370(9596):1453-1457. doi:10.1016/S0140-6736(07)61602-X.
