# Supplementary material for: Selective serotonin reuptake inhibitors and immune checkpoint blockade in solid tumors: A target trial emulation with pan-cancer transcriptomic analyses
Source: PLoS Med. 2026 Sep 29;23(9):e1005262. doi: 10.1371/journal.pmed.1005262 (PMC13645231; doi:10.1371/journal.pmed.1005262)
Supplement: Supplementary file 4 [file pmed.1005262.s004.docx]

**S2 Checklist. The TARGET Checklist**

| **Item no.** | **Checklist item** | **Location reported** |
| --- | --- | --- |
| **Title and abstract** | | |
| **1a** | Identify that the study attempts to emulate a target trial using observational data. State the study objectives and briefly summarize the specified target trial. | Title; Abstract (Background; Methods and Findings) |
| **1b** | Report the data sources used for emulation. | Abstract, Methods and Findings (para 1) |
| **1c** | Summarize key assumptions, statistical methods, findings and conclusions. | Abstract, Methods and Findings and Conclusions |
| **Introduction** | | |
| **2** | Describe the scientific background of the study and the gap in knowledge. | Introduction, paras 1-4 |
| **3** | Summarize the causal question. | Introduction, para 6; Methods, Study Design and Data Source, para 1 |
| **4** | Describe the rationale for emulating a target trial with the available data. Cite randomized trials informing the design of the target trial if applicable. | Introduction, para 5; Methods, Study Design and Data Source, para 1 |
| **Methods** | | |
| **5** | Cite the data sources contributing to the analyses and for each one describe the original purpose, type, geographic locations, setting, and time period. If relevant, describe how the data were linked or pooled. | Methods, Study Design and Data Source, para 1 |
| **6, 7a** | Eligibility criteria: describe the eligibility criteria and how they were operationalized with the data. | Methods, Study Population, para 1; S1 Protocol |
| **6, 7b** | Treatment strategies: describe the treatment strategies compared and how they were operationalized with the data. | Methods, Study Design and Data Source, para 1; Methods, Study Population, para 1 |
| **6, 7c** | Assignment procedures: report that eligible individuals would be randomly assigned, and describe how assignment was emulated with the data. | Methods, Study Design and Data Source, para 2; Methods, Propensity Score Matching, para 1 |
| **6, 7d** | Follow-up: clarify when follow-up would start and end, and how the end of follow-up was operationalized with the data. | Methods, Outcomes, para 1 |
| **6, 7e** | Outcomes: describe the outcomes and how they were operationalized with the data. | Methods, Outcomes, paras 1-2 |
| **6, 7f** | Causal contrasts: describe the causal contrasts of interest (including effect measures) and how they were operationalized with the data. | Methods, Statistical Analysis, para 2 |
| **6, 7g** | Identifying assumptions: describe the assumptions made to identify each causal estimand and the related variables, and how those variables were operationalized. | Methods, Propensity Score Matching, para 1; Methods, Statistical Analysis, para 1; Methods, Study Design and Data Source, para 2 |
| **6, 7h** | Data analysis plan: describe the data analysis procedures, statistical modelling assumptions, handling of missing data, and sensitivity analyses, and how they were operationalized. | Methods, Statistical Analysis, paras 1-2; S1 Protocol |
| **Results** | | |
| **8** | Report numbers of individuals assessed for eligibility, eligible, and assigned to each treatment strategy. A flow diagram is strongly recommended. | Results, Study Cohort and Primary Outcome, para 1; Figure 1 |
| **9** | Describe the distribution of baseline characteristics by treatment strategy. | Results, Study Cohort and Primary Outcome, para 1; Table 1 |
| **10** | Summarize length of follow-up and reasons for end of follow-up for each treatment strategy. | Methods, Outcomes, para 1; Results, Study Cohort and Primary Outcome, para 1 |
| **11** | Describe the frequency of missing data in all variables, by treatment strategy when applicable. | Not reported. The TriNetX platform returns aggregate counts and does not provide per-variable missingness; no imputation was performed. |
| **12** | Describe the frequency or distribution of each outcome, by treatment strategy. | Results, Mechanistic Pathway Outcomes and Organ-Selective irAE, paras 1-5; Table 2 |
| **13** | Report the effect estimates for each causal contrast with measures of precision, including both absolute and relative measures. | Results, Study Cohort and Primary Outcome, para 2; Table 2; Figure 4 |
| **14** | Report results of all analyses assessing the sensitivity of the estimates to operationalizations, assumptions, and analysis. | Results, Subgroup and Sensitivity Analyses, paras 1-4; S1 Fig; S15-S19 Tables |
| **Discussion** | | |
| **15** | Provide an interpretation of the key findings. | Discussion, paras 1-4 |
| **16** | Discuss the limitations of the study, considering differences between the target trial and its emulation and the plausibility of assumptions, including baseline confounding due to lack of randomization. | Discussion, para 5 |
| **Other information** | | |
| **17** | Provide the institutional review board or ethics committee that approved the study and approval numbers, if relevant. | Methods, Study Design and Data Source, para 1; Ethics Approval statement |
| **18** | State whether, when, and where the study protocol was registered. | Methods, Study Design and Data Source, para 2 (pre-specified analysis plan, S1 Protocol); the study was not prospectively registered |
| **19** | Provide information on whether data, analytic code, and/or other materials are accessible, and where and how they can be accessed. | Data Availability Statement |
| **20** | Provide the sources of funding and the role of the funders in the design, conduct, and reporting of the study. | Funding/Support statement |
| **21** | State any conflicts of interest and financial disclosures for all authors. | Conflict of Interest Disclosures statement |

*Locations are given as manuscript section names and paragraph positions (not page numbers). Checklist items 6 (target trial specification) and 7 (target trial emulation) are reported jointly: each protocol component is specified in the pre-specified analysis plan (S1 Protocol) and emulated in the Methods location cited. TARGET, Targeted reporting of emulated trials.*

Citation: Cashin AG, Hansford HJ, Hernán MA, Swanson SA, Lee H, Jones MD, et al. Transparent Reporting of Observational Studies Emulating a Target Trial: The TARGET Statement. JAMA. 2025; DOI: 10.1001/jama.2025.13350
© 2025 Cashin et al. This is an Open Access article distributed under the terms of the Creative Commons Attribution-NoDerivatives License (CC BY-ND 4.0), which permits redistribution, commercial and non-commercial, provided the work is passed along unchanged and in whole, with credit to the original author(s).
