# Supplementary material for: Selective serotonin reuptake inhibitors and immune checkpoint blockade in solid tumors: A target trial emulation with pan-cancer transcriptomic analyses
Source: PLoS Med. 2026 Sep 29;23(9):e1005262. doi: 10.1371/journal.pmed.1005262 (PMC13645231; doi:10.1371/journal.pmed.1005262)
Supplement: Supplementary file 5 [file pmed.1005262.s011.docx]

## **S1 Table. Per-Protocol Sensitivity Analysis: Clinical Outcomes**

*SSRI without BZD vs BZD without SSRI, Per-Protocol Population (continuous medication exposure). N = 695 matched pairs per group.*

| **Outcome** | **SSRI n/N (%)** | **BZD n/N (%)** | **Risk difference** | **Odds Ratio (95% CI)** | **Hazard Ratio (95% CI)** | **P** |
| --- | --- | --- | --- | --- | --- | --- |
| Mortality | 158/695 (22.7%) | 257/695 (37.0%) | −0.142 | 0.501 (0.396, 0.634) | 0.575 (0.472, 0.702) | **<0.001** |
| irAE (composite) | 373/695 (53.7%) | 337/695 (48.5%) | 0.052 | 1.231 (0.997, 1.519) | 1.128 (0.973, 1.307) | 0.109 |
| New systemic corticosteroid | 439/695 (63.2%) | 473/695 (68.1%) | −0.049 | 0.805 (0.645, 1.005) | 0.880 (0.773, 1.002) | 0.054 |
| New metastatic sites ¹ | 55/230 (23.9%) | 64/226 (28.3%) | −0.044 | 0.796 (0.523, 1.210) | 0.822 (0.573, 1.178) | 0.284 |
| TTNT (new Anti-cancer Treatment) | 559/695 (80.4%) | 565/695 (81.3%) | −0.009 | 0.946 (0.724, 1.236) | 0.988 (0.879, 1.111) | 0.836 |
| ICI instances | 498/695 (71.7%) | 507/695 (72.9%) | −0.013 | 0.937 (0.741, 1.186) | 0.986 (0.872, 1.116) | 0.830 |
| ED visits | 231/695 (33.2%) | 259/695 (37.3%) | −0.040 | 0.838 (0.672, 1.045) | 0.842 (0.705, 1.006) | 0.058 |
| Hospitalization | 305/695 (43.9%) | 344/695 (49.5%) | −0.056 | 0.798 (0.646, 0.986) | 0.811 (0.695, 0.946) | **0.008** |
| Palliative care / Hospice | 122/695 (17.6%) | 180/695 (25.9%) | −0.083 | 0.609 (0.470, 0.789) | 0.640 (0.509, 0.806) | **<0.001** |
| New psychiatric diagnosis | 475/695 (68.3%) | 445/695 (64.0%) | 0.043 | 1.213 (0.971, 1.515) | 1.031 (0.906, 1.174) | 0.636 |

*Bold p-values: p<0.05. ¹ Restricted denominator: patients without metastatic sites at baseline. Per-protocol definition: patients with continuous SSRI or BZD exposure throughout the observation period. Mortality HR 0.575 in the per-protocol population, against 0.627 under the intention-to-treat analogue; per-protocol estimates are susceptible to informative censoring and are reported as a sensitivity analysis. Estimates are adjusted by propensity score matching on the 49 baseline covariates listed in S7 Table; unadjusted baseline comparisons for the primary matched cohort are shown in Table 1. The unmatched, before-matching counterpart of every estimate in this table is given in S21 Table.*
