# Supplementary material for: Selective serotonin reuptake inhibitors and immune checkpoint blockade in solid tumors: A target trial emulation with pan-cancer transcriptomic analyses
Source: PLoS Med. 2026 Sep 29;23(9):e1005262. doi: 10.1371/journal.pmed.1005262 (PMC13645231; doi:10.1371/journal.pmed.1005262)
Supplement: Supplementary file 6 [file pmed.1005262.s012.docx]

## **S2 Table. US-Only Sensitivity Analysis: Clinical Outcomes**

*SSRI without BZD vs BZD without SSRI, US healthcare organizations only. N = 1,450 matched pairs per group.*

| **Outcome** | **SSRI n/N (%)** | **BZD n/N (%)** | **Risk difference** | **Odds Ratio (95% CI)** | **Hazard Ratio (95% CI)** | **P** |
| --- | --- | --- | --- | --- | --- | --- |
| Mortality | 334/1,450 (23.0%) | 525/1,450 (36.2%) | −0.132 | 0.527 (0.448, 0.620) | 0.581 (0.507, 0.667) | **<0.001** |
| irAE (composite) | 720/1,450 (49.7%) | 658/1,450 (45.4%) | 0.043 | 1.187 (1.026, 1.374) | 1.084 (0.976, 1.205) | 0.132 |
| New systemic corticosteroid | 847/1,450 (58.4%) | 910/1,450 (62.8%) | −0.043 | 0.834 (0.718, 0.968) | 0.827 (0.753, 0.908) | **<0.001** |
| New metastatic sites ¹ | 92/540 (17.0%) | 95/500 (19.0%) | −0.020 | 0.875 (0.638, 1.202) | 0.860 (0.645, 1.145) | 0.301 |
| TTNT (new Anti-cancer Treatment) | 1,123/1,450 (77.4%) | 1,080/1,450 (74.5%) | 0.030 | 1.177 (0.992, 1.395) | 1.015 (0.934, 1.103) | 0.686 |
| ICI instances | 1,013/1,450 (69.9%) | 953/1,450 (65.7%) | 0.041 | 1.209 (1.034, 1.413) | 1.053 (0.964, 1.151) | 0.206 |
| ED visits | 389/1,450 (26.8%) | 436/1,450 (30.1%) | −0.032 | 0.853 (0.725, 1.002) | 0.818 (0.713, 0.938) | **0.004** |
| Hospitalization | 491/1,450 (33.9%) | 610/1,450 (42.1%) | −0.082 | 0.705 (0.606, 0.820) | 0.719 (0.638, 0.810) | **<0.001** |
| Palliative care / Hospice | 195/1,450 (13.4%) | 327/1,450 (22.6%) | −0.091 | 0.534 (0.439, 0.648) | 0.548 (0.459, 0.654) | **<0.001** |
| New psychiatric diagnosis | 866/1,450 (59.7%) | 810/1,450 (55.9%) | 0.039 | 1.172 (1.011, 1.358) | 1.023 (0.930, 1.126) | 0.630 |

*Bold p-values: p<0.05. ¹ Restricted denominator. US-only analysis ensures regulatory and practice pattern homogeneity. Mortality HR 0.581 in the US-only cohort, against 0.627 in the full cohort. Estimates are adjusted by propensity score matching on the 49 baseline covariates listed in S7 Table; unadjusted baseline comparisons for the primary matched cohort are shown in Table 1. The unmatched, before-matching counterpart of every estimate in this table is given in S21 Table.*
