# Supplementary material for: Selective serotonin reuptake inhibitors and immune checkpoint blockade in solid tumors: A target trial emulation with pan-cancer transcriptomic analyses
Source: PLoS Med. 2026 Sep 29;23(9):e1005262. doi: 10.1371/journal.pmed.1005262 (PMC13645231; doi:10.1371/journal.pmed.1005262)
Supplement: Supplementary file 7 [file pmed.1005262.s013.docx]

## **S3 Table. Dual SSRI+BZD vs BZD-Only Sensitivity Analysis**

*Concurrent SSRI+BZD users vs BZD-only users. N = 2,457 matched pairs per group. This analysis tests whether concurrent BZD use attenuates the SSRI mortality association.*

| **Outcome** | **SSRI+BZD n/N (%)** | **BZD-only n/N (%)** | **Risk difference** | **Odds Ratio (95% CI)** | **Hazard Ratio (95% CI)** | **P** |
| --- | --- | --- | --- | --- | --- | --- |
| Mortality | 931/2,457 (37.9%) | 887/2,457 (36.1%) | 0.018 | 1.080 (0.962, 1.213) | 0.977 (0.891, 1.071) | 0.624 |
| irAE (composite) | 1,227/2,457 (49.9%) | 1,063/2,457 (43.3%) | 0.067 | 1.308 (1.169, 1.464) | 1.154 (1.063, 1.253) | **0.001** |
| New systemic corticosteroid | 1,691/2,457 (68.8%) | 1,545/2,457 (62.9%) | 0.059 | 1.303 (1.158, 1.467) | 1.107 (1.033, 1.186) | **0.004** |
| New metastatic sites ¹ | 180/696 (25.9%) | 154/665 (23.2%) | 0.027 | 1.158 (0.904, 1.483) | 1.076 (0.868, 1.334) | 0.504 |
| TTNT (new Anti-cancer Treatment) | 1,936/2,457 (78.8%) | 1,847/2,457 (75.2%) | 0.036 | 1.227 (1.074, 1.402) | 1.034 (0.970, 1.102) | 0.232 |
| ICI instances | 1,680/2,457 (68.4%) | 1,622/2,457 (66.0%) | 0.024 | 1.113 (0.988, 1.254) | 1.015 (0.948, 1.087) | 0.575 |
| ED visits | 857/2,457 (34.9%) | 718/2,457 (29.2%) | 0.057 | 1.297 (1.150, 1.463) | 1.161 (1.051, 1.281) | **0.003** |
| Hospitalization | 1,238/2,457 (50.4%) | 1,048/2,457 (42.7%) | 0.077 | 1.365 (1.220, 1.528) | 1.168 (1.076, 1.268) | **<0.001** |
| Palliative care / Hospice | 753/2,457 (30.6%) | 605/2,457 (24.6%) | 0.060 | 1.353 (1.193, 1.534) | 1.202 (1.080, 1.338) | **0.001** |
| New psychiatric diagnosis | 1,730/2,457 (70.4%) | 1,341/2,457 (54.6%) | 0.158 | 1.980 (1.761, 2.227) | 1.487 (1.384, 1.597) | **<0.001** |

*Bold p-values: p<0.05. ¹ Restricted denominator. Key finding: Mortality HR 0.977 (p=0.624, NS), the lower mortality observed in the primary analysis (HR 0.627) was not observed with concurrent BZD. However, irAE HR 1.154 (p=0.001) indicates the irAE association persists even with concurrent BZD, suggesting that BZD's GABAergic immunosuppression may offset the mortality association without blocking upstream T-cell activation. Estimates are adjusted by propensity score matching on the 49 baseline covariates listed in S7 Table; unadjusted baseline comparisons for the primary matched cohort are shown in Table 1. The unmatched, before-matching counterpart of every estimate in this table is given in S21 Table.*
