# Supplementary material for: Selective serotonin reuptake inhibitors and immune checkpoint blockade in solid tumors: A target trial emulation with pan-cancer transcriptomic analyses
Source: PLoS Med. 2026 Sep 29;23(9):e1005262. doi: 10.1371/journal.pmed.1005262 (PMC13645231; doi:10.1371/journal.pmed.1005262)
Supplement: Supplementary file 8 [file pmed.1005262.s014.docx]

## **S4 Table. Individual Organ-System Immune-Related Adverse Events**

*N = 1,567 matched pairs. Outcome window: 30–730 days post-ICI initiation. New diagnoses only (not present in 365-day baseline).*

| **Organ System** | **SSRI %** | **BZD %** | **HR (95% CI)** | **KM P** | **Risk P** | **Interpretation** |
| --- | --- | --- | --- | --- | --- | --- |
| Thyroid (E06, E03) | 31.6% | 26.7% | 1.199 (1.053, 1.366) | 0.006 | 0.002 | SSRI ↑ irAE. Primary driver of composite signal. Consistent with serotonergic HPT axis enhancement. |
| Colitis (K52) | 11.8% | 10.0% | 1.130 (0.913, 1.397) | 0.260 | 0.109 | NS. No difference. |
| Hepatitis (K75) | 2.6% | 3.5% | 0.711 (0.474, 1.065) | 0.096 | 0.147 | NS. Low event rate. No safety concern. |
| Pneumonitis (J84) | 4.6% | 4.3% | 1.023 (0.734, 1.427) | 0.892 | 0.664 | NS. Clinically reassuring. |
| Dermatitis (L27, L51) | 4.0% | 3.3% | 1.186 (0.820, 1.716) | 0.364 | 0.252 | NS. |
| Neuropathy (G61, G62) | 11.8% | 15.6% | 0.699 (0.577, 0.846) | <0.001 | 0.002 | SSRI ↓ (lower rate). Likely CIPN, not immune modulation. |
| Adrenal (E27.1, E27.4) | 4.0% | 3.9% | 0.968 (0.680, 1.379) | 0.859 | 0.927 | NS. No difference. |
| New T1DM (E10) | 1.1% | 0.9% | 1.230 (0.612, 2.473) | 0.560 | 0.477 | NS. Very low event rate; underpowered. |

*Organ selectivity: the irAE association was concentrated in the thyroid axis (HPT = hypothalamic-pituitary-thyroid). No significant increase in high-severity irAEs (hepatitis, pneumonitis, colitis). CIPN = chemotherapy-induced peripheral neuropathy. The unmatched, before-matching counterpart of every estimate in this table is given in S21 Table. Estimates are adjusted by propensity score matching on the 49 baseline covariates listed in S7 Table.*
