# Supplementary material for: Selective serotonin reuptake inhibitors and immune checkpoint blockade in solid tumors: A target trial emulation with pan-cancer transcriptomic analyses
Source: PLoS Med. 2026 Sep 29;23(9):e1005262. doi: 10.1371/journal.pmed.1005262 (PMC13645231; doi:10.1371/journal.pmed.1005262)
Supplement: Supplementary file 9 [file pmed.1005262.s015.docx]

## **S5 Table. Complete clinical outcomes after propensity score matching: all 20 prespecified secondary outcomes plus the primary outcome, with event counts.**

*Results for the primary outcome and all 20 prespecified secondary outcomes in the 1:1 propensity-score–matched cohort (1,567 pairs per group): event counts n/N (%), risk ratios, hazard ratios, p values, and mechanistic interpretation, organized by the 4 prespecified mechanistic pathways. E-values are provided in S6 Table.*

| **Outcome** | **SSRI**  **n/N (%)** | **BZD**  **n/N (%)** | **Risk ratio**  **(95% CI)** | **Hazard ratio**  **(95% CI)** | **p** | **Signal** | **Mechanistic interpretation** |
| --- | --- | --- | --- | --- | --- | --- | --- |
| **Primary outcome** | | | | | | | |
| All-cause mortality (2-yr) | 368/1,567 (23.5) | 539/1,567 (34.4) | 0.683 (0.610, 0.764) | 0.627 (0.549, 0.716) | **<0.001** | SSRI ↓↓↓ | 37% lower hazard of death. PH met (p=0.825). |
| **Pathway A: SSRI immune enhancement / tumor control** | | | | | | | |
| Thyroid dysfunction | 495/1,567 (31.6) | 419/1,567 (26.7) | 1.181 (1.059, 1.318) | 1.199 (1.053, 1.366) | **0.006** | SSRI ↑ | Consistent with organ-selective immune enhancement via the HPT axis. |
| Immune-related adverse event (composite) | 771/1,567 (49.2) | 682/1,567 (43.5) | 1.130 (1.048, 1.219) | 1.162 (1.039, 1.299) | **0.008** | SSRI ↑ | Composite irAE significantly increased. Driven by thyroid. |
| New distant metastasis † | 102/595 (17.1) | 142/569 (25.0) | 0.687 (0.547, 0.862) | 0.629 (0.487, 0.811) | **<0.001** | SSRI ↓ | Fewer coded new metastases; consistent with, but does not establish, an antitumor effect (see Discussion). |
| New systemic corticosteroid | 920/1,567 (58.7) | 999/1,567 (63.8) | 0.921 (0.871, 0.974) | 0.819 (0.749, 0.896) | **<0.001** | SSRI ↓ | Likely reflects BZD disease burden. |
| Time to next treatment (TTNT) ‡ | 1,273/1,567 (81.2) | 1,308/1,567 (83.5) | 0.973 (0.942, 1.005) | 0.876 (0.811, 0.946) | **0.001** | SSRI ↓ | Longer time to next-line therapy (progression proxy); ICI continuation not counted, death censored. |
| ICI instances | 1,086/1,567 (69.3) | 1,033/1,567 (65.9) | 1.051 (1.002, 1.104) | 1.035 (0.951, 1.127) | 0.398 | NS | Similar treatment intensity. |
| **Pathway B: disease trajectory / treatment adherence** | | | | | | | |
| Emergency department visits | 432/1,567 (27.6) | 503/1,567 (32.1) | 0.859 (0.771, 0.957) | 0.783 (0.689, 0.891) | **<0.001** | SSRI ↓ | Better disease trajectory. |
| Unplanned hospitalization | 543/1,567 (34.7) | 668/1,567 (42.6) | 0.813 (0.744, 0.889) | 0.730 (0.651, 0.817) | **<0.001** | SSRI ↓↓ | 27% lower hazard. |
| Palliative or hospice referral | 211/1,567 (13.5) | 344/1,567 (22.0) | 0.613 (0.525, 0.717) | 0.563 (0.474, 0.668) | **<0.001** | SSRI ↓↓↓ | 44% lower hazard. PH violation (p=0.032); hazard ratio is a period-averaged effect. |
| New psychiatric diagnosis | 919/1,567 (58.6) | 863/1,567 (55.1) | 1.065 (1.002, 1.132) | 1.024 (0.933, 1.124) | 0.610 | NS | Similar psychiatric stability. |
| Opioid dose escalation | 941/1,567 (60.1) | 1,039/1,567 (66.3) | 0.906 (0.858, 0.956) | 0.779 (0.713, 0.851) | **<0.001** | SSRI ↓ | Ambiguous: tumor control vs SSRI analgesia. |
| Suicidal ideation or self-harm | 17/1,567 (1.1) | 25/1,567 (1.6) | 0.680 (0.369, 1.254) | 0.647 (0.349, 1.198) | 0.163 | NS | Underpowered (17 vs 25 events). |
| **Pathway C: BZD-specific harm** | | | | | | | |
| Delirium or acute confusional state | 115/1,567 (7.3) | 155/1,567 (9.9) | 0.742 (0.589, 0.935) | 0.696 (0.547, 0.886) | **0.003** | SSRI ↓↓ | Direct BZD CNS toxicity evidence. |
| Sepsis or severe infection | 146/1,567 (9.3) | 196/1,567 (12.5) | 0.745 (0.608, 0.912) | 0.699 (0.564, 0.866) | **0.001** | SSRI ↓↓ | Consistent with BZD immunosuppression (TSPO) and/or SSRI enhancement. |
| Venous thromboembolism | 191/1,567 (12.2) | 213/1,567 (13.6) | 0.897 (0.747, 1.076) | 0.859 (0.706, 1.044) | 0.126 | NS | PH violation (p=0.042); hazard ratio is a period-averaged effect. |
| Falls | 137/1,567 (8.7) | 128/1,567 (8.2) | 1.070 (0.850, 1.348) | 1.014 (0.797, 1.290) | 0.913 | NS | BZD sedation–fall harm NOT seen. |
| Fractures | 175/1,567 (11.2) | 162/1,567 (10.3) | 1.080 (0.883, 1.322) | 1.028 (0.830, 1.272) | 0.803 | NS | BZD harm is CNS/immune, not falls. |
| **Pathway D: negative control outcomes** | | | | | | | |
| Nephrolithiasis | 86/1,567 (5.5) | 79/1,567 (5.0) | 1.089 (0.809, 1.466) | 1.042 (0.767, 1.414) | 0.793 | HR≈1 ✓ | Negative control PASS. |
| Cholelithiasis | 83/1,567 (5.3) | 76/1,567 (4.9) | 1.092 (0.806, 1.479) | 1.043 (0.764, 1.424) | 0.792 | HR≈1 ✓ | Negative control PASS. |
| Cataract | 91/1,567 (5.8) | 83/1,567 (5.3) | 1.096 (0.821, 1.464) | 1.047 (0.778, 1.410) | 0.761 | HR≈1 ✓ | Negative control PASS. |

*† New distant metastasis: restricted to patients without baseline metastatic disease. ‡ TTNT: time to a different antineoplastic agent, with continuation or re-dosing of the index ICI not counted as an event and death censored. Bold p-values: p<0.05. PH = proportional hazards. Hazard ratios are estimated within the propensity score-matched cohorts and are therefore adjusted for the 49 baseline covariates used in matching; the complete list is given in S7 Table. The unmatched, before-matching counterpart of every estimate in this table is given in S21 Table.*
