# Supplementary material for: Selective serotonin reuptake inhibitors and immune checkpoint blockade in solid tumors: A target trial emulation with pan-cancer transcriptomic analyses
Source: PLoS Med. 2026 Sep 29;23(9):e1005262. doi: 10.1371/journal.pmed.1005262 (PMC13645231; doi:10.1371/journal.pmed.1005262)
Supplement: Supplementary file 10 [file pmed.1005262.s016.docx]

## **S6 Table. E-value analysis for unmeasured confounding: all outcomes.**

*E-values calculated per VanderWeele and Ding (2017). The E-value represents the minimum strength of association (RR scale) that an unmeasured confounder would need with both the exposure and the outcome to fully explain away the observed association. E-values are shown for the primary outcome and all 20 prespecified secondary outcomes; event counts and risk ratios are provided in S5 Table.*

| **Outcome** | **Hazard ratio**  **(95% CI)** | **p** | **E-value**  **(point)** | **E-value**  **(95% CI)** | **Robustness** |
| --- | --- | --- | --- | --- | --- |
| **Primary outcome** | | | | | |
| All-cause mortality (2-yr) | 0.627 (0.549, 0.716) | <0.001 | 2.57 | 2.14 | Moderate |
| **Pathway A: SSRI immune enhancement / tumor control** | | | | | |
| Thyroid dysfunction | 1.199 (1.053, 1.366) | 0.006 | 1.69 | 1.29 | Modest (HR > 1) |
| Immune-related adverse event (composite) | 1.162 (1.039, 1.299) | 0.008 | 1.60 | 1.24 | Modest (HR > 1) |
| New distant metastasis | 0.629 (0.487, 0.811) | <0.001 | 2.56 | 1.77 | Moderate |
| New systemic corticosteroid | 0.819 (0.749, 0.896) | <0.001 | 1.74 | 1.48 | Modest |
| Time to next treatment (TTNT) | 0.876 (0.811, 0.946) | 0.001 | 1.54 | 1.30 | Modest (HR < 1) |
| ICI instances | 1.035 (0.951, 1.127) | 0.398 | 1.23 | 1.00 | NS |
| **Pathway B: disease trajectory / treatment adherence** | | | | | |
| Emergency department visits | 0.783 (0.689, 0.891) | <0.001 | 1.87 | 1.49 | Modest |
| Unplanned hospitalization | 0.730 (0.651, 0.817) | <0.001 | 2.08 | 1.75 | Moderate |
| Palliative or hospice referral | 0.563 (0.474, 0.668) | <0.001 | 2.95 | 2.36 | Moderate |
| New psychiatric diagnosis | 1.024 (0.933, 1.124) | 0.610 | 1.18 | 1.00 | NS |
| Opioid dose escalation | 0.779 (0.713, 0.851) | <0.001 | 1.89 | 1.63 | Modest |
| Suicidal ideation or self-harm | 0.647 (0.349, 1.198) | 0.163 | 2.46 | 1.00 | NS |
| **Pathway C: BZD-specific harm** | | | | | |
| Delirium or acute confusional state | 0.696 (0.547, 0.886) | 0.003 | 2.23 | 1.51 | Moderate |
| Sepsis or severe infection | 0.699 (0.564, 0.866) | 0.001 | 2.22 | 1.58 | Moderate |
| Venous thromboembolism | 0.859 (0.706, 1.044) | 0.126 | 1.60 | 1.00 | NS |
| Falls | 1.014 (0.797, 1.290) | 0.913 | 1.13 | 1.00 | NS |
| Fractures | 1.028 (0.830, 1.272) | 0.803 | 1.20 | 1.00 | NS |
| **Pathway D: negative control outcomes** | | | | | |
| Nephrolithiasis | 1.042 (0.767, 1.414) | 0.793 | 1.25 | 1.00 | HR ≈ 1.0 ✓ |
| Cholelithiasis | 1.043 (0.764, 1.424) | 0.792 | 1.25 | 1.00 | HR ≈ 1.0 ✓ |
| Cataract | 1.047 (0.778, 1.410) | 0.761 | 1.27 | 1.00 | HR ≈ 1.0 ✓ |

*E-value interpretation: ≥2.0 = moderate robustness; ≥3.0 = strong robustness. E-values were calculated directly from hazard ratios using the formula: E-value = 1/HR + √(1/HR × (1/HR − 1)) for HR <1, and E-value = HR + √(HR × (HR − 1)) for HR >1. Known unmeasured confounders (psychiatric severity [PHQ-9/GAD-7], ECOG performance status, PD-L1/TMB, social support) are unlikely to achieve the association strengths required to explain the mortality finding (E-value = 2.57). Negative control outcomes all show HR ≈ 1.0, further supporting adequate PSM quality. The unmatched, before-matching counterpart of every hazard ratio in this table is given in S21 Table.*
