# Supplementary material for: Selective serotonin reuptake inhibitors and immune checkpoint blockade in solid tumors: A target trial emulation with pan-cancer transcriptomic analyses
Source: PLoS Med. 2026 Sep 29;23(9):e1005262. doi: 10.1371/journal.pmed.1005262 (PMC13645231; doi:10.1371/journal.pmed.1005262)
Supplement: Supplementary file 11 [file pmed.1005262.s017.docx]

## **S7 Table. Propensity Score Matching Covariates**

*Complete list of baseline covariates included in the propensity score model, assessed during the 365-day baseline period before index date. The full covariate balance (before and after matching) is shown in S20 Table.*

| **Category** | **Variables** | **Rationale** |
| --- | --- | --- |
| Demographics (4) | Age (continuous), Sex, Race (White, Black, Asian, Other/Unknown), Ethnicity | Standard confounders |
| Cancer Characteristics (6) | Primary tumor site (C00–C14, C15–C26, C30–C39, C43–C44, C50, C64–C68), Metastatic status (visceral C78, other distant C79), ICI agent class (PD-1, PD-L1, CTLA-4 inhibitors) | Cancer type is the strongest predictor of ICI response |
| Psychiatric Comorbidity (2) | Depressive disorder (F32–F33), Anxiety disorder (F40–F41) | Severity may differ between SSRI and BZD users |
| Medical Comorbidities (12) | Heart failure (I50), Ischemic heart disease (I20–I25), Cerebrovascular disease (I60–I69), Dementia (F03), COPD (J44), Liver disease (K76), Type 2 DM (E11), CKD (N18), Overweight/obesity (E66), Nicotine dependence (F17.2), Alcohol-related disorders (F10), Inflammatory polyarthropathies (M05–M14) | Overall comorbidity burden |
| Baseline Medications (11) | Opioids, NSAIDs, ACE inhibitors, Beta-blockers, Proton-pump inhibitors, Statins, Metformin, Aspirin, Local corticosteroids, Gabapentinoids, Anti-hypertensive medications | Concurrent medications may influence ICI outcomes |
| Laboratory Values (11) | Albumin, Hemoglobin, Platelet count, Leukocytes, Neutrophils, LDH, Creatinine, ALT, AST, Total bilirubin, TSH | Baseline nutritional and inflammatory status |
| Baseline Treatment and Clinical Variables (3) | Systemic chemotherapy, Radiation therapy, Body-mass index | Prior treatment exposure and baseline clinical status |

*PSM specifications: 1:1 greedy nearest-neighbor matching without replacement; caliper 0.1 SD of the logit propensity score; balance threshold SMD < 0.10 for all covariates. Post-matching, all standardized mean differences were < 0.10 (complete covariate balance in S20 Table).*
