# Supplementary material for: Selective serotonin reuptake inhibitors and immune checkpoint blockade in solid tumors: A target trial emulation with pan-cancer transcriptomic analyses
Source: PLoS Med. 2026 Sep 29;23(9):e1005262. doi: 10.1371/journal.pmed.1005262 (PMC13645231; doi:10.1371/journal.pmed.1005262)
Supplement: Supplementary file 12 [file pmed.1005262.s018.docx]

## **S8 Table. Immune Checkpoint Inhibitor Drug Codes**

*All FDA-approved ICI agents included in the study. ICI initiation (first administration) defines the index date.*

| **Generic Name** | **Class** | **Brand Name** | **Route** | **Notes** |
| --- | --- | --- | --- | --- |
| Pembrolizumab | Anti-PD-1 | Keytruda | IV | Most prescribed ICI globally |
| Nivolumab | Anti-PD-1 | Opdivo | IV |  |
| Cemiplimab | Anti-PD-1 | Libtayo | IV |  |
| Dostarlimab | Anti-PD-1 | Jemperli | IV |  |
| Retifanlimab | Anti-PD-1 | Zynyz | IV |  |
| Toripalimab | Anti-PD-1 | Loqtorzi | IV |  |
| Tislelizumab | Anti-PD-1 | Tevimbra | IV |  |
| Atezolizumab | Anti-PD-L1 | Tecentriq | IV |  |
| Durvalumab | Anti-PD-L1 | Imfinzi | IV |  |
| Avelumab | Anti-PD-L1 | Bavencio | IV |  |
| Ipilimumab | Anti-CTLA-4 | Yervoy | IV | First FDA-approved ICI (2011) |
| Tremelimumab | Anti-CTLA-4 | Imjudo | IV |  |
| Nivolumab + Relatlimab | Anti-PD-1 + Anti-LAG-3 | Opdualag | IV | Fixed combination |
