# Supplementary material for: Selective serotonin reuptake inhibitors and immune checkpoint blockade in solid tumors: A target trial emulation with pan-cancer transcriptomic analyses
Source: PLoS Med. 2026 Sep 29;23(9):e1005262. doi: 10.1371/journal.pmed.1005262 (PMC13645231; doi:10.1371/journal.pmed.1005262)
Supplement: Supplementary file 13 [file pmed.1005262.s019.docx]

## **S9 Table. SSRI and Benzodiazepine Drug Codes**

*Complete medication lists for exposure (SSRI) and active comparator (BZD) groups.*

| **Generic Name** | **Brand** | **Mechanistic Notes** | **Typical Dose** | **Group** |
| --- | --- | --- | --- | --- |
| Sertraline | Zoloft | Most prescribed SSRI in US | 50–200 mg/d | SSRI (Exposed) |
| Escitalopram | Lexapro | Highest SERT selectivity | 10–20 mg/d | SSRI (Exposed) |
| Fluoxetine | Prozac | Tested in Cell 2025 + Magagnoli 2023 | 20–80 mg/d | SSRI (Exposed) |
| Paroxetine | Paxil | Anticholinergic properties | 20–60 mg/d | SSRI (Exposed) |
| Citalopram | Celexa | Tested in Cell 2025; GLUT1 inhibitor | 20–40 mg/d | SSRI (Exposed) |
| Fluvoxamine | Luvox | Sigma-1 receptor agonist; less common | 50–300 mg/d | SSRI (Exposed) |
| Lorazepam | Ativan | Short-acting; most common in oncology | 0.5–2 mg PRN | BZD (Comparator) |
| Alprazolam | Xanax | Short-acting; high potency | 0.25–1 mg PRN | BZD (Comparator) |
| Clonazepam | Klonopin | Long-acting; used for GAD | 0.5–2 mg/d | BZD (Comparator) |
| Diazepam | Valium | Long-acting; muscle relaxant | 5–10 mg PRN | BZD (Comparator) |
| Temazepam | Restoril | Intermediate-acting; insomnia | 15–30 mg QHS | BZD (Comparator) |
| Oxazepam | Serax | Short-acting; no active metabolites | 10–30 mg TID | BZD (Comparator) |
| Chlordiazepoxide | Librium | Long-acting; less common | 5–25 mg TID | BZD (Comparator) |

*Mechanistic contrast: SSRIs inhibit SERT, increasing intratumoral serotonin, activating 5-HTR–MAPK–TCR signaling in CD8+ T cells (Li et al., Cell 2025). BZDs are GABA-A receptor agonists with no known serotonergic immune-enhancing mechanism; possible mild immunosuppression via TSPO on lymphocytes.*
