# Supplementary material for: Selective serotonin reuptake inhibitors and immune checkpoint blockade in solid tumors: A target trial emulation with pan-cancer transcriptomic analyses
Source: PLoS Med. 2026 Sep 29;23(9):e1005262. doi: 10.1371/journal.pmed.1005262 (PMC13645231; doi:10.1371/journal.pmed.1005262)
Supplement: Supplementary file 14 [file pmed.1005262.s020.docx]

## **S10 Table. Immune-Related Adverse Event ICD-10 Code Definitions**

*Comprehensive irAE code set synthesized from Wang et al. npj Precision Oncology 2021, Heyward et al. 2025, and JADER database. All codes restricted to new diagnoses 30–730 days after ICI initiation, not present in the 365-day baseline.*

| **Organ System** | **Condition** | **ICD-10-CM** | **Incidence** | **Clinical Notes** | **Definition** |
| --- | --- | --- | --- | --- | --- |
| Endocrine | Hypothyroidism | E03, E03.8, E03.9 | 5–15% | Most common endocrine irAE; often permanent | Narrow + Broad |
| Endocrine | Thyrotoxicosis | E05, E05.0, E05.8, E05.9 | 1–5% | Often transient | Broad only |
| Endocrine | Thyroiditis | E06, E06.3, E06.5, E06.9 | 1–5% | Subacute/painless pattern | Narrow + Broad |
| Endocrine | Adrenal insufficiency | E27.1, E27.4 | 0.5–2% | Can be life-threatening | Narrow + Broad |
| Endocrine | Hypophysitis | E23.0, E23.6, E23.7 | 1–6% | More common with anti-CTLA-4 | Broad only |
| Endocrine | New-onset T1DM | E10, E10.1, E10.65 | <1% | Fulminant onset; can present with DKA | Narrow + Broad |
| GI | Colitis | K52.1, K52.8, K52.9 | 1–12% | Higher with anti-CTLA-4 | Narrow + Broad |
| GI | Pancreatitis | K85, K85.9 | 1–3% | Often asymptomatic lipase only | Broad only |
| Hepatic | Autoimmune hepatitis | K75.4 | 1–10% | Classic irAE pattern | Narrow + Broad |
| Hepatic | Toxic liver disease | K71, K71.1, K71.6 | 1–5% | Drug-induced hepatotoxicity | Broad only |
| Pulmonary | Pneumonitis / ILD | J84.1, J84.8, J84.9 | 2–5% | Most common fatal irAE (35%) | Narrow + Broad |
| Dermatologic | Drug-induced dermatitis | L27.0, L27.1 | 15–40% | Most common irAE overall | Narrow + Broad |
| Dermatologic | SJS / TEN | L51.1, L51.2 | <0.5% | Rare but life-threatening | Narrow + Broad |
| Cardiac | Myocarditis | I40, I40.0, I40.9 | 0.04–1.1% | Highest mortality (~40%) | Narrow + Broad |
| Neurologic | Peripheral neuropathy | G61, G61.0, G62 | 1–5% | Includes CIPN (see S4 Table) | Narrow + Broad |
| Neurologic | Myasthenia gravis | G70.0, G70.00 | <1% | Can be rapidly progressive | Broad only |
| Renal | Nephritis | N10, N11, N12 | 1–2% | Most common renal irAE | Narrow + Broad |
| MSK | Arthralgia / Arthritis | M13, M06, M05 | 5–15% | Most common non-severe irAE | Broad only |
| Hematologic | ITP | D69.3 | <1% | Can be severe | Broad only |
| Ocular | Uveitis | H20, H20.0 | 1–2% | Most common ocular irAE | Broad only |

*Narrow definition (primary): 10 organ systems, ~15 code families. Broad definition (sensitivity): 13 organ systems, ~80+ code families. CIPN = chemotherapy-induced peripheral neuropathy; ITP = immune thrombocytopenia; SJS/TEN = Stevens-Johnson syndrome/toxic epidermal necrolysis.*
