# Supplementary material for: Selective serotonin reuptake inhibitors and immune checkpoint blockade in solid tumors: A target trial emulation with pan-cancer transcriptomic analyses
Source: PLoS Med. 2026 Sep 29;23(9):e1005262. doi: 10.1371/journal.pmed.1005262 (PMC13645231; doi:10.1371/journal.pmed.1005262)
Supplement: Supplementary file 15 [file pmed.1005262.s021.docx]

## **S11 Table. Outcome Definitions and ICD-10 Codes**

*Complete definitions for the primary outcome and all 20 prespecified secondary outcomes, plus the broad immune-related adverse event definition used in sensitivity analysis.*

| **Outcome** | **Definition** | **ICD-10 / Query** | **Role** | **Analysis Notes** |
| --- | --- | --- | --- | --- |
| Mortality (Primary) | Death from any cause | Death record | Primary | 2-yr primary window |
| irAE, Narrow | Composite (10 organ systems) | See S10 Table: Narrow | Key Secondary | New Dx 30–730d post-ICI; not present in the 365-day baseline |
| irAE, Broad | Composite (13 organ systems) | See S10 Table: Broad | Sensitivity | Higher sensitivity definition |
| Thyroid dysfunction | New thyroid immune-related adverse event | E03, E06 | Path A | New Dx 30–730d post-ICI; organ-specific component of the narrow composite |
| New corticosteroid | New systemic corticosteroid Rx | Prednisone, methylpred, dexa | Path A | 30–730d; excl baseline users |
| New metastatic sites | New distant metastasis | C77, C78, C79 | Path A | Restricted denominator |
| TTNT | Time to next-line antineoplastic | New systemic antineoplastic (chemo, PKI, non-ICI mAb/ADC); see footnote | Path A | Progression proxy; death censored; 30–730 d |
| ICI instances | No. of ICI administrations | Any IO agent | Path A | 30–730d; count instances |
| ED visits | Emergency department visit | Visit type: Emergency | Path B | 30–730d |
| Hospitalization | Unplanned inpatient admission | Visit type: Inpatient | Path B | 30–730d |
| Palliative care | Palliative / hospice referral | Z51.5 | Path B | Time to first occurrence |
| New psychiatric Dx | New psychiatric diagnosis | F32, F33, F41, F43 | Path B | 30–730d; new Dx post-index |
| Opioid escalation | New or stronger opioid | New opioid Rx post-index | Path B | None at baseline OR new class |
| Suicidal ideation | Suicidal ideation / self-harm | R45.851, T14.91, X71–X83 | Path B | 30–730d |
| Falls | Falls | W00–W19, R29.6 | Path C | 30–730d |
| Fractures | Fractures (traumatic or osteoporotic fragility) | S12/S22/S32/S42/S52/S62/S72/S82/S92, M80 | Path C | Excludes pathological fractures in neoplastic disease (M84.5) |
| Delirium | Delirium / acute confusion | F05, R41.0, R41.82 | Path C | 30–730d |
| Sepsis | Sepsis / severe infection | A41, R65.2, A49 | Path C | 30–730d; dual Path A/C signal |
| VTE | Venous thromboembolism | I26 (PE), I82 (DVT) | Path C | 30–730d |
| Kidney stone | Nephrolithiasis | N20 | Path D (Neg Ctrl) | Excludes prior stones; 30–730d |
| Gallstone | Cholelithiasis | K80 | Path D (Neg Ctrl) | Excludes prior GB stones; 30–730d |
| Cataract | Cataract | H25, H26 | Path D (Neg Ctrl) | Excludes prior cataract; 30–730d |

*Path A = SSRI immune enhancement / tumor control; Path B = better mental health / treatment adherence; Path C = BZD-specific harm; Path D = negative control outcomes (confounding test).*

*TTNT (time to next treatment): event = initiation of a new systemic anticancer agent not part of the baseline regimen: chemotherapy (ATC L01XA, L01CD, L01DB, L01D, plus pemetrexed and gemcitabine), a protein kinase inhibitor (L01E), or a non-ICI therapeutic monoclonal antibody/antibody-drug conjugate (L01FA, L01FB, L01FC, L01FD, L01FE, L01FG, L01FY); continued or re-initiated immune checkpoint inhibitors (L01FF, L01FX) were not counted as events; death was censored; window 30–730 days. HR <1 indicates longer time to next treatment with SSRI.*
