# Supplementary material for: Selective serotonin reuptake inhibitors and immune checkpoint blockade in solid tumors: A target trial emulation with pan-cancer transcriptomic analyses
Source: PLoS Med. 2026 Sep 29;23(9):e1005262. doi: 10.1371/journal.pmed.1005262 (PMC13645231; doi:10.1371/journal.pmed.1005262)
Supplement: Supplementary file 16 [file pmed.1005262.s022.docx]

## S12 Table. TCGA Per-Cancer Survival Analysis: SLC6A4 Low versus High

| **Cancer** | **HR** | **95% CI** | **P** | **n** | **Events** |
| --- | --- | --- | --- | --- | --- |
| PRAD | 0.649 | 0.183, 2.302 | 0.500 | 502 | 10 |
| KIRC | 0.793 | 0.588, 1.070 | 0.129 | 537 | 173 |
| UCEC | 0.801 | 0.531, 1.209 | 0.291 | 549 | 92 |
| ESCA | 0.822 | 0.522, 1.293 | 0.395 | 183 | 76 |
| COAD | 0.851 | 0.570, 1.271 | 0.431 | 457 | 97 |
| LIHC | 0.905 | 0.641, 1.278 | 0.571 | 366 | 130 |
| LUSC | 0.928 | 0.710, 1.212 | 0.584 | 504 | 219 |
| SKCM | 0.998 | 0.753, 1.323 | 0.992 | 360 | 194 |
| BLCA | 1.008 | 0.753, 1.350 | 0.955 | 405 | 181 |
| SARC | 1.069 | 0.719, 1.589 | 0.740 | 256 | 98 |
| KIRP | 1.080 | 0.596, 1.959 | 0.799 | 287 | 44 |
| STAD | 1.099 | 0.806, 1.499 | 0.549 | 387 | 160 |
| BRCA | 1.132 | 0.823, 1.556 | 0.446 | 1,087 | 152 |
| READ | 1.136 | 0.528, 2.445 | 0.745 | 157 | 27 |
| LUAD | 1.219 | 0.915, 1.625 | 0.176 | 527 | 188 |
| HNSC | 1.272 | 0.975, 1.661 | 0.076 | 519 | 221 |
| CESC | 1.407 | 0.882, 2.245 | 0.151 | 291 | 72 |
| **PAAD** | 1.698 | 1.123, 2.566 | **0.011** | 178 | 93 |

Cox proportional hazards regression with SLC6A4 median split per cancer type; hazard ratios are expressed for SLC6A4-low versus high (HR >1 indicates worse survival with low SLC6A4). Random-effects meta-analysis (REML, Hartung-Knapp CI): pooled HR = 1.04 (95% CI [0.94, 1.15]), p=0.42, I² = 13.0%, 18 cancer types. Bold indicates p<0.05. PAAD was the only cancer type reaching statistical significance.
