# Supplementary material for: Selective serotonin reuptake inhibitors and immune checkpoint blockade in solid tumors: A target trial emulation with pan-cancer transcriptomic analyses
Source: PLoS Med. 2026 Sep 29;23(9):e1005262. doi: 10.1371/journal.pmed.1005262 (PMC13645231; doi:10.1371/journal.pmed.1005262)
Supplement: Supplementary file 17 [file pmed.1005262.s023.docx]

## **S13 Table. Gene Set Definitions Used in the TCGA Analyses**

*Source: analysis code (available on GitHub; see the Data Availability statement).*

**A. Immune Function Scores (Phase 2 analysis, Figure 5A and S5 Fig)**

| **Gene Set** | **N** | **Genes** | **Source** |
| --- | --- | --- | --- |
| TCELL_INFLAMED_GEP | 18 | *CD8A, CD8B, PDCD1, CTLA4, LAG3, TIGIT, CD27, CXCL9, CXCL10, CCL5, IDO1, PSMB10, NKG7, HLA-E, CMKLR1, PDCD1LG2, CD276, STAT1* | Ayers et al., J Clin Invest 2017 |
| CYTOTOXIC_SCORE | 10 | *GZMB, GZMA, GZMK, GZMH, PRF1, IFNG, TNF, FASLG, NKG7, CX3CR1* | Rooney et al., Cell 2015 (adapted) |
| SEROTONIN_AXIS | 11 | *SLC6A4, TPH1, TPH2, DDC, MAOA, MAOB, HTR1A, HTR2A, HTR3A, HTR7, SLC18A2* | Custom |
| IFNG6 | 6 | *IFNG, STAT1, IDO1, CXCL9, CXCL10, HLA-DRA* | Custom |

**B. Immune Phenotype & Cell-Type Scores (Exclusion analysis, Figure 5B-C)**

| **Gene Set** | **N** | **Genes** | **Source** |
| --- | --- | --- | --- |
| Excluded Stroma | 7 | *TGFB1, TGFB2, WNT5A, FAP, FN1, COL1A1, ACTA2* | Mariathasan et al., Nature 2018 (adapted) |
| CD8+ T cell | 4 | *CD8A, CD8B, GZMB, PRF1* | Consensus markers |
| CD4+ T cell | 3 | *CD4, IL2RA, FOXP3* | Consensus markers |
| Treg | 4 | *FOXP3, IL2RA, CTLA4, IKZF2* | Plitas & Rudensky 2020 (adapted) |
| NK cell | 4 | *NKG7, GNLY, KLRB1, KLRD1* | Consensus markers |
| M2 Macrophage | 4 | *CD163, MRC1, IL10, TGFB1* | Consensus markers |
| CAF | 4 | *FAP, FN1, ACTA2, S100A4* | Costa et al., Cancer Cell 2018 (adapted) |

**Phenotype Classification Logic:**

Inflamed: TCELL_INFLAMED_GEP > per-cancer median AND Excluded Stroma ≤ median

Excluded: Excluded Stroma > per-cancer median (regardless of T-cell score)

Desert: TCELL_INFLAMED_GEP ≤ median AND Excluded Stroma ≤ median
