# Supplementary material for: Selective serotonin reuptake inhibitors and immune checkpoint blockade in solid tumors: A target trial emulation with pan-cancer transcriptomic analyses
Source: PLoS Med. 2026 Sep 29;23(9):e1005262. doi: 10.1371/journal.pmed.1005262 (PMC13645231; doi:10.1371/journal.pmed.1005262)
Supplement: Supplementary file 18 [file pmed.1005262.s024.docx]

## **S14 Table. Organ-Level Cross-Validation: Mapping Clinical irAE to TCGA Cancer Types**

| **Organ** | **ICD-10** | **irAE HR (95% CI)** | **P** | **TCGA** | **rho** | **FDR** | **Rationale** |
| --- | --- | --- | --- | --- | --- | --- | --- |
| **Thyroid** | E03, E06 | 1.199 (1.053, 1.366) | **0.006** | THCA | -0.335 | <0.001 | Direct: thyroid gland |
| Colon | K52 | 1.130 (0.913, 1.397) | 0.260 | COAD | -0.326 | <0.001 | Direct: colonic mucosa |
| Skin | L27, L51 | 1.186 (0.820, 1.716) | 0.364 | SKCM | -0.100 | 0.073 | Direct: cutaneous |
| Lung | J84 | 1.023 (0.734, 1.427) | 0.892 | LUAD | -0.022 | 0.726 | LUAD preferred for pneumonitis |
| Liver | K75 | 0.711 (0.474, 1.065) | 0.096 | LIHC | -0.000 | 0.997 | Direct: hepatocyte |

*Excluded organ systems: Neuropathy (G61, G62) - primarily CIPN, not immune-mediated; Adrenal (E27) - no corresponding cancer type among the 20 analyzed; New-onset T1DM (E10) - very rare (17 vs 14 events). All rho values from the analysis code (TcellGEP score); all HR values from clinical arm S4 Table.*
