# Supplementary material for: Selective serotonin reuptake inhibitors and immune checkpoint blockade in solid tumors: A target trial emulation with pan-cancer transcriptomic analyses
Source: PLoS Med. 2026 Sep 29;23(9):e1005262. doi: 10.1371/journal.pmed.1005262 (PMC13645231; doi:10.1371/journal.pmed.1005262)
Supplement: Supplementary file 19 [file pmed.1005262.s025.docx]

**S15 Table. SSRI users versus patients receiving neither an SSRI nor a benzodiazepine.**

Third comparator arm. 1:1 propensity-score matching on the same 49 covariates (1,551 pairs; all standardized mean differences <0.10).

| **Outcome** | **HR (95% CI)** | **p value** |
| --- | --- | --- |
| All-cause mortality | 0.753 (0.605, 0.939) | 0.011 |
| Immune-related adverse event (composite) | 1.116 (1.008, 1.235) | 0.034 |
| New systemic corticosteroid | 1.152 (1.049, 1.264) | 0.003 |
| New distant metastasis | 1.016 (0.776, 1.331) | 0.909 |
| Time to next treatment | 0.936 (0.865, 1.013) | 0.123 |
| Emergency department visit | 1.047 (0.921, 1.190) | 0.479 |
| Unplanned hospitalization | 1.048 (0.933, 1.178) | 0.426 |
| Palliative or hospice referral | 1.056 (0.886, 1.259) | 0.544 |
| New psychiatric diagnosis | 1.599 (1.446, 1.768) | <0.001 |

*HR <1 indicates lower risk with SSRI versus the neither-agent comparator. Hazard ratios from Cox models. SSRI use remained associated with lower mortality than no psychotropic treatment. The unmatched, before-matching counterpart of every estimate in this table is given in S21 Table. Estimates are adjusted by propensity score matching on the 49 baseline covariates listed in S7 Table.*
