# Supplementary material for: Selective serotonin reuptake inhibitors and immune checkpoint blockade in solid tumors: A target trial emulation with pan-cancer transcriptomic analyses
Source: PLoS Med. 2026 Sep 29;23(9):e1005262. doi: 10.1371/journal.pmed.1005262 (PMC13645231; doi:10.1371/journal.pmed.1005262)
Supplement: Supplementary file 20 [file pmed.1005262.s026.docx]

**S16 Table. Overall survival by immune checkpoint inhibitor class (SSRI vs benzodiazepine).**

| **ICI class** | **OS HR (95% CI)** | **Log-rank P** | **PH (Schoenfeld) P** |
| --- | --- | --- | --- |
| Anti-PD-1 | 0.683 (0.591, 0.790) | <0.001 | 0.85 |
| Anti-PD-L1 | 0.657 (0.549, 0.786) | <0.001 | 0.21 |
| Anti-CTLA-4 | 0.692 (0.451, 1.060) | 0.089 | 0.41 |
| Combination | 0.890 (0.536, 1.477) | 0.652 | 0.68 |
| Pooled (descriptive) | 0.682 (0.613, 0.759) | - | - |

*Heterogeneity: Cochran Q = 1.23, df = 3, p=0.75; I² = 0%. Each row: SSRI vs benzodiazepine within the indicated ICI-class subcohort after 1:1 propensity-score matching; Cox model, 30-day landmark. Because the ICI-class subcohorts are not mutually exclusive, the pooled estimate and I² are descriptive, not a formal test of between-class consistency. The unmatched, before-matching counterpart of every estimate in this table is given in S21 Table. Estimates are adjusted by propensity score matching on the 49 baseline covariates listed in S7 Table.*
