# Supplementary material for: Selective serotonin reuptake inhibitors and immune checkpoint blockade in solid tumors: A target trial emulation with pan-cancer transcriptomic analyses
Source: PLoS Med. 2026 Sep 29;23(9):e1005262. doi: 10.1371/journal.pmed.1005262 (PMC13645231; doi:10.1371/journal.pmed.1005262)
Supplement: Supplementary file 21 [file pmed.1005262.s027.docx]

**S17 Table. Imaging-surveillance frequency and supplementary negative-control outcomes (SSRI vs benzodiazepine).**

| **Outcome** | **SSRI n (%)** | **BZD n (%)** | **HR (95% CI)** | **p value** |
| --- | --- | --- | --- | --- |
| Imaging surveillance (>=1 study) | 944 (64.6) | 953 (65.2) | 0.957 (0.875, 1.047) | 0.338 |
| Refractive error (negative control) | 49 (3.4) | 33 (2.3) | 1.455 (0.936, 2.262) | 0.094 |
| Sebaceous cyst (negative control) | 14 (1.0) | 13 (0.9) | 1.048 (0.493, 2.230) | 0.903 |

*Imaging = chest, abdominopelvic, and other CT, MRI, PET-CT, and bone scintigraphy during follow-up (30-730 d). Comparable imaging frequency argues against differential detection of metastases. Percentages are computed within the matched groups of this analysis, which was run on a contemporaneous re-extraction of the network whose matched cohort size differs from the 1,567 pairs of the primary analysis (S1 Protocol, Section 11). Refractive error and sebaceous cyst are post hoc additional negative control outcomes, reported separately from the 3 prespecified path D negative controls (nephrolithiasis, cholelithiasis, cataract; S5 Table). The sebaceous cyst estimate is close to the null (HR 1.048; 95% CI [0.493, 2.230]; p=0.903); the refractive error estimate is not (HR 1.455; 95% CI [0.936, 2.262]; p=0.094). The unmatched, before-matching counterpart of every estimate in this table is given in S21 Table. Estimates are adjusted by propensity score matching on the 49 baseline covariates listed in S7 Table.*
