# Supplementary material for: Selective serotonin reuptake inhibitors and immune checkpoint blockade in solid tumors: A target trial emulation with pan-cancer transcriptomic analyses
Source: PLoS Med. 2026 Sep 29;23(9):e1005262. doi: 10.1371/journal.pmed.1005262 (PMC13645231; doi:10.1371/journal.pmed.1005262)
Supplement: Supplementary file 23 [file pmed.1005262.s029.docx]

**S19 Table. Overall survival excluding post-baseline drug-class crossover (SSRI vs benzodiazepine).**

| **Outcome** | **SSRI events/N** | **BZD events/N** | **HR (95% CI)** | **p value** |
| --- | --- | --- | --- | --- |
| All-cause mortality (no crossover) | 349/1,402 | 497/1,402 | 0.668 (0.583, 0.767) | <0.001 |

*Patients who initiated the opposite drug class during follow-up were excluded. Confirms mutual-exclusivity was defined at baseline and that post-baseline crossover does not drive the result. The unmatched, before-matching counterpart of every estimate in this table is given in S21 Table. Estimates are adjusted by propensity score matching on the 49 baseline covariates listed in S7 Table.*
