# Supplementary material for: Selective serotonin reuptake inhibitors and immune checkpoint blockade in solid tumors: A target trial emulation with pan-cancer transcriptomic analyses
Source: PLoS Med. 2026 Sep 29;23(9):e1005262. doi: 10.1371/journal.pmed.1005262 (PMC13645231; doi:10.1371/journal.pmed.1005262)
Supplement: Supplementary file 24 [file pmed.1005262.s030.docx]

**S20 Table. Complete baseline characteristics and covariate balance before and after propensity score matching (all covariates).**

*Baseline characteristics for every covariate entered into the propensity score model. Data are n (%) unless otherwise indicated (mean ± s.d. for continuous variables). Cohort 1, SSRI without concurrent BZD; Cohort 2, BZD without concurrent SSRI. p values: chi-squared (categorical) or independent-samples t-test (continuous); SMD < 0.10 indicates adequate balance and is the preferred metric. Operational ICD-10-CM definitions are in S7 Table.*

|  | **Before matching** | | | | **After matching** | | | |
| --- | --- | --- | --- | --- | --- | --- | --- | --- |
| **Characteristic** | **SSRI** (n = 1,661) | **BZD** (n = 5,500) | ***p value*** | **SMD** | **SSRI** (n = 1,567) | **BZD** (n = 1,567) | ***p value*** | **SMD** |
| ***Demographics*** |  |  |  |  |  |  |  |  |
| Age at index date, yr, mean ± s.d. | 66.9 ± 12.4 | 63.2 ± 12.3 | <0.001 | 0.301 | 66.4 ± 12.4 | 66.5 ± 11.2 | 0.933 | 0.003 |
| *Sex* |  |  |  |  |  |  |  |  |
| Female | 926 (55.7) | 3,137 (57.0) | 0.354 | 0.026 | 880 (56.2) | 893 (57.0) | 0.639 | 0.017 |
| Male | 735 (44.3) | 2,363 (43.0) | 0.354 | 0.026 | 687 (43.8) | 674 (43.0) | 0.639 | 0.017 |
| *Race or ethnic group* |  |  |  |  |  |  |  |  |
| White | 1,396 (84.0) | 4,355 (79.2) | <0.001 | 0.126 | 1,314 (83.9) | 1,316 (84.0) | 0.923 | 0.003 |
| Black or African American | 115 (6.9) | 464 (8.4) | 0.047 | 0.057 | 111 (7.1) | 107 (6.8) | 0.779 | 0.010 |
| Asian | 28 (1.7) | 287 (5.2) | <0.001 | 0.194 | 28 (1.8) | 26 (1.7) | 0.784 | 0.010 |
| Unknown or other | 76 (4.6) | 248 (4.5) | 0.909 | 0.003 | 70 (4.5) | 71 (4.5) | 0.931 | 0.003 |
| *Ethnicity* |  |  |  |  |  |  |  |  |
| Hispanic or Latino | 66 (4.0) | 154 (2.8) | 0.011 | 0.066 | 67 (4.3) | 67 (4.3) | 1.00 | <0.001 |
| Not Hispanic or Latino | 1,309 (78.8) | 4,521 (82.2) | 0.001 | 0.087 | 1,280 (81.7) | 1,258 (80.3) | 0.368 | 0.037 |
| Unknown ethnicity | 286 (17.2) | 820 (14.9) | 0.022 | 0.061 | 219 (14.0) | 241 (15.4) | 0.319 | 0.041 |
| ***Primary tumor site*** |  |  |  |  |  |  |  |  |
| Head and neck | 102 (6.1) | 436 (7.9) | 0.015 | 0.070 | 102 (6.5) | 105 (6.7) | 0.829 | 0.008 |
| Gastrointestinal tract | 349 (21.0) | 1,081 (19.7) | 0.225 | 0.034 | 327 (20.9) | 329 (21.0) | 0.930 | 0.003 |
| Thoracic | 543 (32.7) | 2,283 (41.5) | <0.001 | 0.183 | 534 (34.1) | 545 (34.8) | 0.679 | 0.015 |
| Melanoma or skin | 351 (21.1) | 939 (17.1) | <0.001 | 0.103 | 333 (21.3) | 330 (21.1) | 0.896 | 0.005 |
| Breast | 133 (8.0) | 569 (10.3) | 0.005 | 0.081 | 131 (8.4) | 141 (9.0) | 0.526 | 0.023 |
| Genitourinary | 370 (22.3) | 790 (14.4) | <0.001 | 0.206 | 324 (20.7) | 315 (20.1) | 0.690 | 0.014 |
| ***Metastatic disease at baseline*** |  |  |  |  |  |  |  |  |
| Visceral metastasis | 434 (26.1) | 1,774 (32.3) | <0.001 | 0.135 | 419 (26.7) | 417 (26.6) | 0.936 | 0.003 |
| Other distant metastasis | 483 (29.1) | 2,083 (37.9) | <0.001 | 0.187 | 473 (30.2) | 446 (28.5) | 0.289 | 0.038 |
| ***Psychiatric diagnosis*** |  |  |  |  |  |  |  |  |
| Depressive disorder | 1,193 (71.8) | 2,212 (40.2) | <0.001 | 0.672 | 1,099 (70.1) | 1,091 (69.6) | 0.755 | 0.011 |
| Anxiety disorder | 1,018 (61.3) | 4,641 (84.4) | <0.001 | 0.538 | 1,012 (64.6) | 1,007 (64.3) | 0.852 | 0.007 |
| ***Comorbidities*** |  |  |  |  |  |  |  |  |
| Ischemic heart disease | 469 (28.2) | 1,308 (23.8) | <0.001 | 0.102 | 437 (27.9) | 431 (27.5) | 0.811 | 0.009 |
| Heart failure | 216 (13.0) | 596 (10.8) | 0.015 | 0.067 | 201 (12.8) | 194 (12.4) | 0.706 | 0.013 |
| Cerebrovascular disease | 258 (15.5) | 700 (12.7) | 0.003 | 0.081 | 239 (15.3) | 255 (16.3) | 0.433 | 0.028 |
| Dementia | 45 (2.7) | 70 (1.3) | <0.001 | 0.103 | 40 (2.6) | 39 (2.5) | 0.909 | 0.004 |
| COPD | 401 (24.1) | 1,299 (23.6) | 0.660 | 0.012 | 384 (24.5) | 389 (24.8) | 0.836 | 0.007 |
| Chronic liver disease | 331 (19.9) | 1,162 (21.1) | 0.292 | 0.030 | 317 (20.2) | 315 (20.1) | 0.929 | 0.003 |
| Type 2 diabetes mellitus | 505 (30.4) | 1,226 (22.3) | <0.001 | 0.185 | 453 (28.9) | 459 (29.3) | 0.813 | 0.008 |
| Chronic kidney disease | 347 (20.9) | 823 (15.0) | <0.001 | 0.155 | 313 (20.0) | 297 (19.0) | 0.470 | 0.026 |
| Overweight or obesity | 422 (25.4) | 1,059 (19.3) | <0.001 | 0.148 | 389 (24.8) | 370 (23.6) | 0.428 | 0.028 |
| Tobacco use disorder | 413 (24.9) | 1,545 (28.1) | 0.010 | 0.073 | 403 (25.7) | 414 (26.4) | 0.654 | 0.016 |
| Alcohol use disorder | 134 (8.1) | 375 (6.8) | 0.082 | 0.048 | 125 (8.0) | 131 (8.4) | 0.696 | 0.014 |
| Inflammatory polyarthropathies | 123 (7.4) | 402 (7.3) | 0.888 | 0.004 | 121 (7.7) | 124 (7.9) | 0.816 | 0.010 |
| ***Baseline treatment and clinical data*** |  |  |  |  |  |  |  |  |
| Systemic chemotherapy | 1,655 (99.6) | 5,474 (99.5) | 0.550 | 0.017 | 1,561 (99.6) | 1,561 (99.6) | 1.000 | <0.001 |
| Radiation therapy | 420 (25.3) | 1,807 (32.9) | <0.001 | 0.167 | 413 (26.4) | 413 (26.4) | 1.000 | <0.001 |
| Body-mass index, kg/m², mean ± s.d. | 28.1 ± 7.1 | 27.0 ± 6.9 | <0.001 | 0.150 | 28.0 ± 7.2 | 27.7 ± 7.1 | 0.340 | 0.037 |
| ***Medications*** |  |  |  |  |  |  |  |  |
| Opioids | 1,432 (86.2) | 4,868 (88.5) | 0.008 | 0.070 | 1,357 (86.6) | 1,362 (86.9) | 0.725 | 0.009 |
| NSAIDs | 311 (18.7) | 1,001 (18.2) | 0.682 | 0.011 | 340 (21.7) | 351 (22.4) | 0.689 | 0.017 |
| ACE inhibitors | 365 (22.0) | 902 (16.4) | <0.001 | 0.141 | 356 (22.7) | 356 (22.7) | 0.960 | 0.002 |
| Beta-blocker | 231 (13.9) | 963 (17.5) | <0.001 | 0.100 | 270 (17.2) | 279 (17.8) | 0.702 | 0.016 |
| Proton pump inhibitors | 849 (51.1) | 2,728 (49.6) | 0.253 | 0.031 | 834 (53.2) | 838 (53.5) | 0.901 | 0.005 |
| Statin | 832 (50.1) | 1,936 (35.2) | <0.001 | 0.305 | 784 (50.0) | 799 (51.0) | 0.648 | 0.019 |
| Metformin | 186 (11.2) | 440 (8.0) | <0.001 | 0.108 | 171 (10.9) | 165 (10.5) | 0.788 | 0.011 |
| Aspirin | 475 (28.6) | 1,309 (23.8) | <0.001 | 0.110 | 494 (31.5) | 481 (30.7) | 0.654 | 0.019 |
| Local corticosteroids | 1,344 (80.9) | 4,697 (85.4) | <0.001 | 0.121 | 1,382 (88.2) | 1,368 (87.3) | 0.526 | 0.026 |
| Gabapentinoids | 500 (30.1) | 1,689 (30.7) | 0.633 | 0.013 | 550 (35.1) | 534 (34.1) | 0.631 | 0.020 |
| Anti-hypertensive medication | 1,133 (68.2) | 3,856 (70.1) | 0.140 | 0.041 | 1,098 (70.1) | 1,100 (70.2) | 0.874 | 0.003 |
| PD-1 inhibitor | 1,259 (75.8) | 3,955 (71.9) | 0.004 | 0.089 | 1,180 (75.3) | 1,175 (75.0) | 0.900 | 0.005 |
| PD-L1 inhibitor | 282 (17.0) | 1,029 (18.7) | 0.116 | 0.044 | 268 (17.1) | 254 (16.2) | 0.360 | 0.024 |
| CTLA-4 inhibitor | 125 (7.5) | 501 (9.1) | 0.049 | 0.055 | 110 (7.0) | 116 (7.4) | 0.688 | 0.017 |
| ***Laboratory values*** |  |  |  |  |  |  |  |  |
| Leukocytes, 10³/µL | 13.5 ± 10.6 | 11.4 ± 11.6 | <0.001 | 0.192 | 11.9 ± 11.3 | 12.1 ± 11.0 | 0.717 | 0.016 |
| Platelets, 10³/µL | 265.4 ± 117.2 | 272.4 ± 122.5 | 0.039 | 0.058 | 263.4 ± 116.5 | 274.5 ± 120.8 | 0.027 | 0.094 |
| Creatinine, mg/dL | 1.7 ± 1.6 | 2.0 ± 1.6 | 0.101 | 0.188 | 1.5 ± 1.2 | 1.6 ± 1.8 | 0.232 | 0.065 |
| Neutrophils, 10³/µL | 2.5 ± 2.4 | 2.7 ± 1.4 | <0.001 | 0.102 | 2.6 ± 1.2 | 2.4 ± 1.3 | 0.118 | 0.060 |
| ALT, U/L | 24.4 ± 26.1 | 28.8 ± 21.5 | 0.033 | 0.184 | 23.9 ± 25.2 | 26.0 ± 45.8 | 0.171 | 0.058 |
| AST, U/L | 29.2 ± 34.9 | 35.4 ± 26.2 | 0.110 | 0.201 | 28.7 ± 28.5 | 30.7 ± 51.2 | 0.266 | 0.047 |
| Total bilirubin, mg/dL | 0.6 ± 0.6 | 0.7 ± 2.7 | 0.018 | 0.051 | 0.6 ± 0.6 | 0.7 ± 1.9 | 0.081 | 0.075 |
| LDH, U/L | 252.5 ± 131.7 | 310.6 ± 137.7 | 0.001 | 0.431 | 247.1 ± 139.1 | 252.2 ± 131.3 | 0.562 | 0.038 |
| Thyrotropin (TSH), mIU/L | 3.9 ± 1.9 | 4.3 ± 1.5 | 0.648 | 0.234 | 4.2 ± 1.1 | 4.3 ± 1.4 | 0.292 | 0.079 |
| Hemoglobin, g/dL | 11.7 ± 2.0 | 11.7 ± 2.1 | 0.483 | 0.020 | 11.6 ± 2.0 | 11.4 ± 2.1 | 0.023 | 0.097 |
| Albumin, g/dL | 3.7 ± 0.6 | 3.7 ± 0.6 | 0.027 | 0.064 | 3.7 ± 0.6 | 3.6 ± 0.6 | 0.101 | 0.067 |

BZD, benzodiazepine; COPD, chronic obstructive pulmonary disease; s.d., standard deviation; SMD, standardized mean difference; SSRI, selective serotonin reuptake inhibitor; ACE, angiotensin-converting enzyme; ALT, alanine aminotransferase; AST, aspartate aminotransferase; CTLA-4, cytotoxic T-lymphocyte-associated protein 4; LDH, lactate dehydrogenase; NSAID, non-steroidal anti-inflammatory drug; PD-1, programmed cell death protein 1; PD-L1, programmed death-ligand 1; TSH, thyrotropin.
