# Supplementary material for: Selective serotonin reuptake inhibitors and immune checkpoint blockade in solid tumors: A target trial emulation with pan-cancer transcriptomic analyses
Source: PLoS Med. 2026 Sep 29;23(9):e1005262. doi: 10.1371/journal.pmed.1005262 (PMC13645231; doi:10.1371/journal.pmed.1005262)
Supplement: Supplementary file 25 [file pmed.1005262.s031.docx]

**S21 Table. Hazard ratios before and after propensity score matching, all cohort comparisons and prespecified outcomes.**

***Before-matching estimates are from unmatched analyses of the full eligible cohorts; after-matching estimates are as reported in the manuscript. Hazard ratios are from Cox proportional hazards models; p values are from the log-rank test. Outcome window: day 30 to 730 after the index date unless otherwise stated.***

| **Outcome** | **Before matching HR (95% CI)** | **p value** | **After matching HR (95% CI)** | **p value** |
| --- | --- | --- | --- | --- |
| **Panel A. Primary comparison: SSRI without benzodiazepine versus benzodiazepine without SSRI (Table 2, S5 Table)** | | | | |
| All-cause mortality | 0.611 (0.553, 0.674) | <0.001 | 0.627 (0.549, 0.716) | <0.001 |
| Immune-related adverse event (composite) | 1.138 (1.059, 1.223) | <0.001 | 1.162 (1.039, 1.299) | 0.008 |
| Thyroid dysfunction | 1.312 (1.196, 1.439) ² | <0.001 | 1.199 (1.053, 1.366) | 0.006 |
| New systemic corticosteroid | 0.793 (0.743, 0.847) | <0.001 | 0.819 (0.749, 0.896) | <0.001 |
| New distant metastasis ¹ | 0.802 (0.662, 0.971) | 0.024 | 0.629 (0.487, 0.811) | <0.001 |
| ICI instances | 1.050 (0.988, 1.116) | 0.088 | 1.035 (0.951, 1.127) | 0.398 |
| Emergency department visit | 0.822 (0.751, 0.900) | <0.001 | 0.783 (0.689, 0.891) | <0.001 |
| Unplanned hospitalization | 0.704 (0.648, 0.764) | <0.001 | 0.730 (0.651, 0.817) | <0.001 |
| Palliative or hospice referral | 0.593 (0.524, 0.673) | <0.001 | 0.563 (0.474, 0.668) | <0.001 |
| New psychiatric diagnosis | 1.089 (1.020, 1.163) | 0.010 | 1.024 (0.933, 1.124) | 0.610 |
| Opioid escalation | 0.748 (0.702, 0.798) | <0.001 | 0.779 (0.713, 0.851) | <0.001 |
| Suicidal ideation | 0.906 (0.580, 1.414) | 0.664 | 0.647 (0.349, 1.198) | 0.163 |
| Falls | 1.256 (1.071, 1.473) | 0.005 | 1.014 (0.797, 1.290) | 0.913 |
| Fractures | 1.083 (0.931, 1.260) | 0.302 | 1.028 (0.830, 1.272) | 0.803 |
| Delirium or acute confusional state | 0.679 (0.570, 0.808) | <0.001 | 0.696 (0.547, 0.886) | 0.003 |
| Sepsis or severe infection | 0.691 (0.592, 0.806) | <0.001 | 0.699 (0.564, 0.866) | 0.001 |
| Venous thromboembolism | 0.808 (0.701, 0.931) | 0.003 | 0.859 (0.706, 1.044) | 0.126 |
| Nephrolithiasis | 1.177 (0.945, 1.467) | 0.145 | 1.042 (0.767, 1.414) | 0.793 |
| Cholelithiasis | 1.024 (0.820, 1.279) | 0.833 | 1.043 (0.764, 1.424) | 0.792 |
| Cataract | 1.001 (0.784, 1.279) | 0.991 | 1.047 (0.778, 1.410) | 0.761 |
| **Panel B. Per-protocol population (S1 Table)** | | | | |
| All-cause mortality | 0.549 (0.486, 0.620) | <0.001 | 0.575 (0.472, 0.702) | <0.001 |
| Immune-related adverse event (composite) | 1.115 (1.023, 1.214) | 0.013 | 1.128 (0.973, 1.307) | 0.109 |
| New systemic corticosteroid | 0.783 (0.724, 0.847) | <0.001 | 0.880 (0.773, 1.002) | 0.054 |
| New distant metastasis ¹ | 0.750 (0.596, 0.943) | 0.013 | 0.822 (0.573, 1.178) | 0.284 |
| TTNT (new anti-cancer treatment) | 0.943 (0.879, 1.011) | 0.091 | 0.988 (0.879, 1.111) | 0.836 |
| ICI instances | 1.033 (0.959, 1.112) | 0.347 | 0.986 (0.872, 1.116) | 0.830 |
| Emergency department visit | 0.853 (0.770, 0.946) | 0.003 | 0.842 (0.705, 1.006) | 0.058 |
| Unplanned hospitalization | 0.689 (0.627, 0.757) | <0.001 | 0.811 (0.695, 0.946) | 0.008 |
| Palliative or hospice referral | 0.567 (0.491, 0.655) | <0.001 | 0.640 (0.509, 0.806) | <0.001 |
| New psychiatric diagnosis | 1.017 (0.942, 1.098) | 0.650 | 1.031 (0.906, 1.174) | 0.636 |
| **Panel C. US Collaborative Network only (S2 Table)** | | | | |
| All-cause mortality | 0.597 (0.539, 0.661) | <0.001 | 0.581 (0.507, 0.667) | <0.001 |
| Immune-related adverse event (composite) | 1.107 (1.029, 1.191) | 0.006 | 1.084 (0.976, 1.205) | 0.132 |
| New systemic corticosteroid | 0.794 (0.743, 0.849) | <0.001 | 0.827 (0.753, 0.908) | <0.001 |
| New distant metastasis ¹ | 0.795 (0.652, 0.970) | 0.023 | 0.860 (0.645, 1.145) | 0.301 |
| TTNT (new anti-cancer treatment) | 0.999 (0.941, 1.060) | 0.995 | 1.015 (0.934, 1.103) | 0.686 |
| ICI instances | 1.076 (1.011, 1.145) | 0.013 | 1.053 (0.964, 1.151) | 0.206 |
| Emergency department visit | 0.850 (0.774, 0.933) | 0.001 | 0.818 (0.713, 0.938) | 0.004 |
| Unplanned hospitalization | 0.727 (0.667, 0.791) | <0.001 | 0.719 (0.638, 0.810) | <0.001 |
| Palliative or hospice referral | 0.562 (0.495, 0.638) | <0.001 | 0.548 (0.459, 0.654) | <0.001 |
| New psychiatric diagnosis | 1.061 (0.993, 1.135) | 0.078 | 1.023 (0.930, 1.126) | 0.630 |
| **Panel D. Dual SSRI plus benzodiazepine versus benzodiazepine without SSRI (S3 Table)** | | | | |
| All-cause mortality | 1.103 (1.035, 1.176) | 0.003 | 0.977 (0.891, 1.071) | 0.624 |
| Immune-related adverse event (composite) | 1.228 (1.161, 1.299) | <0.001 | 1.154 (1.063, 1.253) | 0.001 |
| New systemic corticosteroid | 1.123 (1.070, 1.179) | <0.001 | 1.107 (1.033, 1.186) | 0.004 |
| New distant metastasis ¹ | 1.045 (0.899, 1.214) | 0.566 | 1.076 (0.868, 1.334) | 0.504 |
| TTNT (new anti-cancer treatment) | 1.044 (0.998, 1.092) | 0.035 | 1.034 (0.970, 1.102) | 0.232 |
| ICI instances | 1.001 (0.954, 1.051) | 0.803 | 1.015 (0.948, 1.087) | 0.575 |
| Emergency department visit | 1.145 (1.072, 1.222) | <0.001 | 1.161 (1.051, 1.281) | 0.003 |
| Unplanned hospitalization | 1.206 (1.141, 1.276) | <0.001 | 1.168 (1.076, 1.268) | <0.001 |
| Palliative or hospice referral | 1.341 (1.243, 1.447) | <0.001 | 1.202 (1.080, 1.338) | 0.001 |
| New psychiatric diagnosis | 1.589 (1.512, 1.669) | <0.001 | 1.487 (1.384, 1.597) | <0.001 |
| **Panel E. SSRI versus neither SSRI nor benzodiazepine (S15 Table)** | | | | |
| All-cause mortality | 0.516 (0.459, 0.581) | <0.001 | 0.753 (0.605, 0.939) | 0.011 |
| Immune-related adverse event (composite) | 1.113 (1.022, 1.212) | 0.013 | 1.116 (1.008, 1.235) | 0.034 |
| New systemic corticosteroid | 1.136 (1.068, 1.208) | <0.001 | 1.152 (1.049, 1.264) | 0.003 |
| New distant metastasis ¹ | 0.857 (0.749, 0.980) | 0.024 | 1.016 (0.776, 1.331) | 0.909 |
| Time to next treatment | 0.830 (0.781, 0.881) | <0.001 | 0.936 (0.865, 1.013) | 0.123 |
| Emergency department visit | 0.868 (0.808, 0.933) | <0.001 | 1.047 (0.921, 1.190) | 0.479 |
| Unplanned hospitalization | 1.452 (1.352, 1.560) | <0.001 | 1.048 (0.933, 1.178) | 0.426 |
| Palliative or hospice referral | 2.164 (1.933, 2.422) | <0.001 | 1.056 (0.886, 1.259) | 0.544 |
| New psychiatric diagnosis | 1.964 (1.845, 2.090) | <0.001 | 1.599 (1.446, 1.768) | <0.001 |
| **Panel F. Additional sensitivity analyses of the primary comparison (Table 2, S5, S17, S18 and S19 Tables)** | | | | |
| Time to next treatment (next-line agent) (Table 2, S5 Table) | 0.875 (0.827, 0.925) | <0.001 | 0.876 (0.811, 0.946) | 0.001 |
| Imaging surveillance (≥1 study) (S17 Table) | 0.909 (0.857, 0.964) | 0.002 | 0.957 (0.875, 1.047) | 0.338 |
| Refractive error (negative control) (S17 Table) | 1.087 (0.836, 1.412) | 0.534 | 1.455 (0.936, 2.262) | 0.094 |
| Sebaceous cyst (negative control) (S17 Table) | 1.312 (0.944, 1.823) | 0.105 | 1.048 (0.493, 2.230) | 0.903 |
| All-cause mortality, no time-window restriction (S18 Table) | 0.618 (0.567, 0.672) | <0.001 | 0.596 (0.532, 0.667) | <0.001 |
| All-cause mortality, excluding crossover (S19 Table) | 0.622 (0.561, 0.689) | <0.001 | 0.668 (0.583, 0.767) | <0.001 |
| **Panel G. Individual organ-system immune-related adverse events (S4 Table)** | | | | |
| Thyroid dysfunction | 1.312 (1.196, 1.439) | <0.001 | 1.199 (1.053, 1.366) | 0.006 |
| Colitis | 1.126 (0.969, 1.308) | 0.121 | 1.130 (0.913, 1.397) | 0.260 |
| Hepatitis | 1.015 (0.756, 1.362) | 0.923 | 0.711 (0.474, 1.065) | 0.096 |
| Pneumonitis | 1.013 (0.805, 1.277) | 0.910 | 1.023 (0.734, 1.427) | 0.892 |
| Dermatitis | 0.946 (0.740, 1.210) | 0.659 | 1.186 (0.820, 1.716) | 0.364 |
| Neuropathy | 0.814 (0.709, 0.934) | 0.003 | 0.699 (0.577, 0.846) | <0.001 |
| Adrenal | 1.009 (0.786, 1.295) | 0.944 | 0.968 (0.680, 1.379) | 0.859 |
| New T1DM | 1.079 (0.684, 1.702) | 0.744 | 1.230 (0.612, 2.473) | 0.560 |
| **Panel H. Overall survival by immune checkpoint inhibitor class (S16 Table)** | | | | |
| Overall survival, anti-PD-1 | 0.604 (0.540, 0.676) | <0.001 | 0.683 (0.591, 0.790) | <0.001 |
| Overall survival, anti-PD-L1 | 0.592 (0.518, 0.677) | <0.001 | 0.657 (0.549, 0.786) | <0.001 |
| Overall survival, anti-CTLA-4 | 0.593 (0.432, 0.814) | 0.001 | 0.692 (0.451, 1.060) | 0.089 |
| Overall survival, combination | 0.584 (0.425, 0.802) | 0.001 | 0.890 (0.536, 1.477) | 0.652 |

***¹ Restricted denominator: patients with the outcome before the start of the time window were excluded, so the before-matching denominators for these rows are smaller than the full cohorts.***

***² The before-matching thyroid immune-related adverse event estimate is taken from the organ-level analysis in Panel G.***

***Effect directions agree across the two columns for 69 of the 77 estimates; in each of the 8 exceptions the matched confidence interval includes 1. p values returned by the platform as 0.000 are shown as p<0.001.***

***BZD, benzodiazepine; CI, confidence interval; HR, hazard ratio; ICI, immune checkpoint inhibitor; SSRI, selective serotonin reuptake inhibitor; T1DM, type 1 diabetes mellitus.***
