# Supplementary material for: Selective serotonin reuptake inhibitors and immune checkpoint blockade in solid tumors: A target trial emulation with pan-cancer transcriptomic analyses
Source: PLoS Med. 2026 Sep 29;23(9):e1005262. doi: 10.1371/journal.pmed.1005262 (PMC13645231; doi:10.1371/journal.pmed.1005262)
Supplement: Supplementary file 26 [file pmed.1005262.s032.docx]

**Response to the Editors**

**Manuscript PMEDICINE-D-26-00932R2**

*Serotonin transporter inhibition and immune checkpoint blockade outcomes: a target trial emulation with pan-cancer transcriptomic validation*

We thank the Senior Editor, the Academic Editor and the three reviewers for the time they have given this manuscript. Below we quote each remaining editorial and production request, followed by our response and the specific changes made. All changes are marked in the accompanying marked-up manuscript. The scientific content and every numerical result are unchanged from the previous revision.

**GENERAL EDITORIAL REQUESTS**

**Comment 1**

*Please confirm that your title complies with PLOS Medicine's style. Your title must be nondeclarative and not a question. It should begin with main concept if possible. "Effect of" should be used only if causality can be inferred, i.e., for an RCT. Please place the study design ("A randomized controlled trial," "A retrospective study," "A modelling study," etc.) in the subtitle (ie, after a colon).*

**Response:** We confirm that the title complies. It is nondeclarative and is not a question, it opens with the main concept (serotonin transporter inhibition and immune checkpoint blockade outcomes), it does not use "effect of" or any other causal construction, and the study design appears in the subtitle after the colon. The title is 140 characters, within the 200-character limit.

**Changes made:**

• No change was required. The title is unchanged: "Serotonin transporter inhibition and immune checkpoint blockade outcomes: a target trial emulation with pan-cancer transcriptomic validation".

**Comment 2**

*Please confirm that your abstract complies with our requirements, including format (three sections: Background, Methods and Findings, and Conclusions) and providing all the information relevant to this study type.*

**Response:** The abstract previously carried four sections (Background, Methods and Findings, Results, Conclusions). We have merged the Results text into Methods and Findings so that the abstract now has exactly the three required sections. The abstract is 429 words, within the 500-word maximum.

**Changes made:**

• Abstract: the separate "Results" heading has been removed and its content merged into "Methods and Findings".

• Abstract, Methods and Findings: eligibility criteria, the primary outcome, the number of prespecified secondary outcomes, the matching procedure, and the transcriptomic dataset are all stated before the findings.

**Comment 3**

*Please ensure that the Introduction ends with a clear description of the study question or hypothesis.*

**Response:** We have added an explicit statement of the study question and hypothesis as the closing sentence of the Introduction.

**Changes made:**

• Introduction, final paragraph: added "We asked whether the choice of psychotropic agent at ICI initiation is associated with subsequent outcomes, and hypothesized that, among patients with depression or anxiety who initiate an ICI, initiation of an SSRI rather than a benzodiazepine is associated with lower 2-year mortality, and that any such association is accompanied by an organ-selective increase in immune-related adverse events in the tissues where SLC6A4 expression tracks most closely with immune suppression."

**Comment 4**

*Please ensure that all abbreviations are defined at first use throughout the text.*

**Response:** We audited every abbreviation in the manuscript against its first occurrence and expanded those that had been left undefined.

**Changes made:**

• Introduction: "mitogen-activated protein kinase (MAPK)" now expanded.

• Methods: "Eastern Cooperative Oncology Group (ECOG) performance status" and "programmed death ligand 1 (PD-L1)" now expanded; "standardized mean differences (SMDs)" now defined where balance is first reported; "International Classification of Diseases, Tenth Revision, Clinical Modification (ICD-10-CM)" defined in the eligibility criteria.

• Results: the TCGA project codes are expanded at first use (bladder urothelial carcinoma [BLCA], thyroid carcinoma [THCA], colon adenocarcinoma [COAD], pancreatic adenocarcinoma [PAAD], liver hepatocellular carcinoma [LIHC]); "natural killer (NK) cell" expanded.

• Discussion: "central nervous system (CNS)" expanded.

• Abstract: "anti-programmed cell death protein 1 (anti-PD-1)", "hazard ratios (HRs)" and "95% confidence intervals (CIs)" defined at first use.

**Comment 5**

*Please confirm that all numbers presented in the abstract are present and identical to numbers presented in the main manuscript text.*

**Response:** We re-extracted every number in the abstract from the manuscript body and the supplement and confirmed that each one matches. The abstract reports the primary outcome, the composite and thyroid immune-related adverse event estimates, the metastasis estimate, the negative controls, the individual-agent hazard ratio range, the matched sample size, and the TCGA sample and tumor-type counts, all identical to the corresponding values in the Results, Table 2, and S4 to S5 Tables.

**Changes made:**

• No numerical change was required. Confidence intervals that were previously omitted from the abstract are now reported in full (see the statistical reporting item below).

**GENERAL**

**Comment 6**

*Please include a statement regarding use or non-use of AI tools and technologies at the end of the Methods. Please specify the name(s) and version(s) of any tool(s) used and a description of their use, including how the AI-assisted or generated content was evaluated by authors, and a clear statement of which aspects of the study, article contents, data, or supporting files were affected or generated by AI tool usage. Please also include a statement specifying who retains responsibility for the final manuscript.*

**Response:** We have added an artificial intelligence statement as the final paragraph of the Methods, naming the tool, describing its use, stating how the authors evaluated the output, and stating who retains responsibility for the manuscript.

**Changes made:**

• Methods, final paragraph ("Use of artificial intelligence tools"): Claude (Anthropic) was used, through the Claude Code interface between 2025 and August 2026, for language editing of the manuscript text and for assistance in writing and debugging the R code used in the transcriptomic analyses, with successive releases of the Claude Opus and Claude Sonnet models over that period, most recently Claude Opus 5; no AI tool was used to generate, extract, or interpret the clinical or transcriptomic data; all AI-assisted text was checked against the underlying results and revised by the authors, and all AI-assisted code was executed by the authors and its output verified against the source data; the authors retain full responsibility for the content of the manuscript.

**Comment 7**

*Please review your text for claims of novelty or primacy (e.g. 'for the first time') and remove this language. In addition, please check that any use of statistical terms (such as trend or significant) are supported by the data, and if not please remove them.*

**Response:** We removed the remaining novelty and primacy language and the one unsupported use of "trend".

**Changes made:**

• Introduction: "a novel immune checkpoint" is now "an immune checkpoint"; the sentence claiming that the absence of large-scale evidence "represents a critical gap" has been rewritten neutrally.

• Abstract, Background: "novel" removed for the same reason.

• Results, subgroup paragraph: "the only subgroup without a protective trend" is now "the only subgroup in which the estimate did not favor SSRI use"; the same wording was applied to the S1 Fig caption.

• Figure 5 legend: "Trend line is exploratory" is now "The fitted line is exploratory".

• Every remaining use of "significant" refers to an estimate whose p value and confidence interval are reported in Table 2, S5 Table or the corresponding supplementary table.

**Comment 8**

*Statistical reporting: Please revise throughout the manuscript, including tables and figures. Please report statistical information as follows to improve clarity for the reader "22% (95% CI [13,28]; p</=)". Please separate upper and lower bounds with commas instead of hyphens as the latter can be confused with reporting of negative values. Please repeat statistical definitions (HR, CI etc.) for each set of parentheses.*

**Response:** We adopted the requested format throughout. In the running text every effect estimate is now reported as "(HR 0.627; 95% CI [0.549, 0.716]; p<0.001)", with the statistic named inside each set of parentheses. In the tables, where the column header already supplies the container (for example "Hazard ratio (95% CI)"), we changed the range separator from a hyphen to a comma and left the header convention intact. We also added confidence intervals to every estimate in the Results that previously reported only a hazard ratio and a p value.

**Changes made:**

• Manuscript: all 25 hyphenated confidence intervals converted; hazard ratios now written "HR 0.627" rather than "HR, 0.627".

• Results: confidence intervals added for hepatitis, pneumonitis, colitis, emergency department visits, opioid escalation, new psychiatric diagnoses, falls, fractures, the three negative controls, the dual-exposure immune-related adverse event estimate, each ICI class, and the PAAD survival estimate (values taken from S3 to S5 and S16 Tables).

• Tables 1 and 2 and all supplement tables: range separators converted to commas (24 cells in Table 2, 87 in the supplement, 18 in the S12 Table confidence-interval column).

• Figures: Figure 4 (pathway forest) and S6 Fig (ICI-class forest) were regenerated so the in-plot confidence intervals read "0.627 (0.549, 0.716)".

**Comment 9**

*In the last sentence of the Abstract Methods and Findings section, please describe the main limitation(s) of the study's methodology.*

**Response:** We added a closing limitation sentence to Methods and Findings.

**Changes made:**

• Abstract, Methods and Findings, final sentence: "The main limitation of this study is residual confounding: performance status is not recorded in the network, and BZDs are often prescribed in different clinical circumstances from SSRIs, so the groups may differ in ways that matching cannot capture."

**Comment 10**

*In the author summary, please revise formatting and ensure you use bullet points.*

**Response:** The Author Summary has been rebuilt as three bulleted lists, with three single-sentence bullets under each of the three required headings, and technical language removed.

**Changes made:**

• Author summary: "Why was this study done?", "What did the researchers do and find?" and "What do these findings mean?" each now carry 3 bullet points.

**Comment 11**

*In the author summary, in the final bullet point of 'What Do These Findings Mean?', please include the main limitations of the study in non-technical language.*

**Response:** The final bullet now states the main limitation in plain language.

**Changes made:**

• Author summary, final bullet: "The records do not capture how well or unwell a person was when treatment started, and benzodiazepines are often given at times of acute distress or worsening illness, so part of the difference may reflect how ill people were rather than the medicine itself."

• The preceding bullet also states that, because people were not randomly assigned, the results show an association and cannot show that SSRIs caused the better outcomes.

**FUNDING STATEMENT**

**Comment 12**

*The funding statement should include: specific grant numbers, initials of authors who received each award, URLs to sponsors' websites. Also, please state whether any sponsors or funders (other than the named authors) played any role in study design, data collection and analysis, the decision to publish, or preparation of the manuscript. If they had no role in the research, include this sentence: "The funders had no role in study design, data collection and analysis, decision to publish, or preparation of the manuscript."*

**Response:** The funding statement now carries the grant numbers, the recipient initials, and the sponsor URLs. On the role of the funders, five of the eight authors are affiliated with the funding institutions, so the journal's standard no-role sentence does not apply unmodified; the manuscript instead names those affiliations and states that the funders had no other role in study design, data collection and analysis, the decision to publish, or preparation of the manuscript. No sponsor or funder outside the author list had any role in the study.

**Changes made:**

• Funding/Support: "This study was supported by Tri-Service General Hospital (grant TSGH-D-115176 to PHC; https://www.tsgh.ndmutsgh.edu.tw) and by a collaborative research grant between Cheng Hsin General Hospital (https://www.chgh.org.tw) and National Defense Medical University (https://www.ndmutsgh.edu.tw) (grant CHNDMU-115-02 to CHL)."

• Role of the Funder/Sponsor: "PHC, MSD, LTK, and CHL are affiliated with Tri-Service General Hospital and National Defense Medical University, and MHH is affiliated with Cheng Hsin General Hospital, which are among the institutions that funded this study. The funders had no other role in study design, data collection and analysis, decision to publish, or preparation of the manuscript."

• The Funding/Support text has been entered in the Financial Disclosure field of the submission form.

**Comment 13**

*It appears that one or more study authors is affiliated with one or more of the agencies that funded the study. Thus, the statement "The funders had no role in study design, data collection and analysis, decision to publish, or preparation of the manuscript" does not apply. Please revise the Financial Disclosure accordingly, as in "[Author name] is [author's role] at [funding agency]. The funders had no other role in study design....."*

**Response:** Correct: five of the eight authors are affiliated with the funding institutions. We revised the statement to the form the journal requests.

**Changes made:**

• Role of the Funder/Sponsor: "PHC, MSD, LTK, and CHL are affiliated with Tri-Service General Hospital and National Defense Medical University, and MHH is affiliated with Cheng Hsin General Hospital, which are among the institutions that funded this study. The funders had no other role in study design, data collection and analysis, decision to publish, or preparation of the manuscript."

**COMPETING INTERESTS STATEMENT**

**Comment 14**

*All authors must declare their relevant competing interests per the PLOS policy. For authors with ties to industry, please indicate whether any of the interests has a financial stake in the results of the current study. Please amend your competing interests statement. Funding information should go to the relevant section and include URLs.*

**Response:** All authors have reviewed the PLOS competing interests policy and none has a competing interest to declare; no author has ties to industry or a financial stake in the results. The statement now uses the journal's standard wording, and it contains no funding information, which is confined to the funding statement.

**Changes made:**

• Conflict of Interest Disclosures: "The authors have declared that no competing interests exist."

**DATA AVAILABILITY**

**Comment 15**

*PLOS defines the "minimal data set" to consist of the data set used to reach the conclusions drawn in the manuscript with related metadata and methods, and any additional data required to replicate the reported study findings in their entirety. Authors do not need to submit their entire data set, or the raw data collected during an investigation. Please submit the following data: The values behind the means, standard deviations and other measures reported; The values used to build graphs; The points extracted from images for analysis.*

**Response:** For the clinical arm, every aggregate value behind each figure and table, including the event counts, risks, hazard ratios, confidence intervals and p values for all 21 outcomes, is reported in the manuscript and its Supporting Information; patient-level records cannot be exported from the TriNetX federated network under the data use agreement. For the transcriptomic arm, we have deposited the derived data files that underlie Figure 5A, S3 Fig and S5 Fig alongside the analysis code, so each plotted point can be traced to a numeric value.

**Changes made:**

• Data Availability Statement: now states explicitly that all aggregate values behind every figure and table are reported within the article and its Supporting Information files.

• The derived data files underlying Figure 5A, S3 Fig and S5 Fig (per-cancer SLC6A4-immune correlations, per-cancer survival estimates, and the serotonin-pathway gene-by-signature correlations and rankings) are included in the code repository and its archived release. The values behind the remaining transcriptomic panels are reproducible from the same code and the public GDC inputs, and the organ-level cross-validation values are printed in S14 Table.

**Comment 16**

*For studies in which a novel model is central to the manuscript's findings, as is the case here, authors are responsible for providing the source code needed to replicate the study's findings in a repository (such as GitHub, SourceForge or Bitbucket) or a cloud computing service (such as Code Ocean). Please include a citable digital object identifier (DOI) or other persistent identifier of the code (such as through linkage with Zenodo). Protection of authors' intellectual property will not be cause for exception. Please explain in the manuscript's Data Availability Statement how readers can access the shared code. Please include the statement on code availability in the data availability statement.*

**Response:** All analysis code for the transcriptomic validation is public on GitHub and has been archived to obtain a citable DOI. The Data Availability Statement now explains where the code lives and how to obtain it.

**Changes made:**

• Data Availability Statement: "All analysis code for the transcriptomic validation, together with the derived data files that underlie Figure 5A, S3 Fig and S5 Fig, is publicly available in the GitHub repository https://github.com/chenpohuang/ssri-ici-transcriptomics and is permanently archived in Zenodo (DOI 10.5281/zenodo.21882308)."

**Comment 17**

*Thank you for agreeing to make your data available. At this time, please provide the link to the data repository and accession numbers required for access.*

**Response:** The transcriptomic data are public and we now list the exact accessions. The clinical data are third-party and we give the contact route.

**Changes made:**

• Data Availability Statement: names the National Cancer Institute Genomic Data Commons (https://portal.gdc.cancer.gov) and lists all 20 TCGA project accessions used (TCGA-BLCA, TCGA-BRCA, TCGA-CESC, TCGA-COAD, TCGA-ESCA, TCGA-HNSC, TCGA-KICH, TCGA-KIRC, TCGA-KIRP, TCGA-LIHC, TCGA-LUAD, TCGA-LUSC, TCGA-PAAD, TCGA-PRAD, TCGA-READ, TCGA-SARC, TCGA-SKCM, TCGA-STAD, TCGA-THCA, TCGA-UCEC).

• For the clinical data it gives the TriNetX URL, states that the authors held only a standard institutional license with no special access privileges, and states that any qualified investigator can obtain the same access.

**ETHICS AND CONSENT**

**Comment 18**

*Please revise for use of patient-centered language. Please note that patient-centered language is constructed with the use of post-modified nouns (e.g. 'patients with psoriasis' (or similar) instead of 'psoriasis patients') putting the person first in the sentence structure.*

**Response:** We converted every pre-modified construction in the manuscript body to a post-modified one. Reference-list entries were left verbatim because they are published article titles.

**Changes made:**

• Introduction: "cancer patients receiving immunotherapy" is now "patients with cancer who receive immunotherapy".

• Introduction, Methods, Discussion and Conclusions: "ICI-treated solid tumor patients" is now "patients with a solid tumor and depression or anxiety who were receiving ICIs".

• Discussion: "SSRI-treated patients" is now "patients treated with SSRIs".

• No condition-first constructions of this kind remain in the manuscript body.

**FIGURES**

**Comment 19**

*When a p value is given, please specify the statistical test used to determine it in the legend.*

**Response:** Every figure legend that reports a p value now names the test.

**Changes made:**

• Figure 3 legend: log-rank test stated for the survival comparison.

• Figure 4 legend and the figure’s own footnote: "Hazard ratios are from Cox proportional hazards models; p values are from the log-rank test." The figure was regenerated so that the wording inside the image matches the legend and the Table 2 footnote.

• Figure 5 legend: Spearman correlation and Kruskal-Wallis test were already named; the thyroid irAE p value is now attributed to the log-rank test.

• S1 Fig, S2 Fig, S3 Fig, S5 Fig and S6 Fig captions: Cox proportional hazards models, the test of Schoenfeld residuals, random-effects meta-analysis, Spearman correlation with Benjamini-Hochberg correction, and the Cochran Q test named as appropriate.

**Comment 20**

*Where data points are discrete, please ensure that they are depicted in the figures as discrete data and not as a continuous line.*

**Response:** We checked each panel. Figure 3b, the only panel presenting values at discrete follow-up times, is already drawn as grouped bars with error bars at 3, 6, 9, 12, 18 and 24 months rather than as a connected line. Figure 3a is a Kaplan-Meier curve, where the step function over continuous follow-up time is the correct representation. Figure 5D plots 5 discrete organ points, with the fitted line labeled exploratory in the legend.

**Changes made:**

• No change was required.

**Comment 21**

*Please provide the unadjusted comparisons as well as the adjusted comparisons in all relevant Tables.*

**Response:** Table 1 already reports the unadjusted, before-matching comparison of the two cohorts for the baseline characteristics it lists, and S20 Table does so for all 49 matching covariates, including the medications and laboratory values that Table 1 does not show. For the outcomes, we have now generated the unmatched counterpart of every matched analysis in the paper and report them together in a new S21 Table, which places the before-matching and after-matching hazard ratio side by side for all 77 outcome estimates across eight cohort comparisons. We report them in a single table rather than as extra columns in each affected table so that the individual tables remain readable. Every affected table now points to S21 Table.

**Changes made:**

• New S21 Table: before- and after-matching hazard ratios, 95% confidence intervals and p values for all 77 outcome estimates, across eight comparisons (primary; per-protocol; US-only; dual SSRI plus benzodiazepine; neither-agent comparator; additional sensitivity analyses; individual organ-system immune-related adverse events; and immune checkpoint inhibitor class).

• Table 2 footnote, in the manuscript and in the standalone table file: states that the hazard ratios are estimated within the matched cohorts and are therefore adjusted for the 49 matching covariates, points the reader to Table 1 for the unadjusted before-matching baseline comparison, and points to S21 Table for the before-matching counterpart of every outcome estimate.

• S1, S2, S3, S4, S5, S15, S16, S17, S18 and S19 Table footnotes: the same pointer to S21 Table added.

• Data Availability Statement: scoped so that the event-count claim applies to the matched analyses, since S21 Table reports hazard ratios, confidence intervals and p values.

**Comment 22**

*Please specify the variables controlled for in all relevant Tables.*

**Response:** The matching covariates are now itemized in the table footnotes rather than only in the Methods.

**Changes made:**

• Table 2 footnote, the standalone Table 2 file, and the S1 to S5 and S15 to S19 Table footnotes state the variables controlled for; the fullest form, in Table 2, reads "adjusted for the 49 baseline covariates used in matching (demographics, tumor characteristics, psychiatric diagnosis type, 12 comorbidities, 11 medications, 11 laboratory values, and 3 baseline treatment and clinical variables; the complete list is given in S7 Table)".

**Comment 23**

*Please consider if moving some of the figures shared in the supplementary information into the main text would aid the reader. I am referring to Sup F1 and F2 to be Figure 1 and 2 as this will give potential reader good overview of your study from the beginning. When you do that please revise if remaining reference are correct in whole manuscript.*

**Response:** We agree and have made the change. The patient selection flow diagram and the target trial emulation schematic are now Figure 1 and Figure 2 of the main text, so the reader meets the cohort and the design before the results. Every figure reference in the manuscript, supplement, protocol and figure file names was renumbered accordingly and re-checked.

**Changes made:**

• New main text: Figure 1 (patient selection flow diagram, formerly S1 Fig) and Figure 2 (target trial emulation study design, formerly S2 Fig), each with a full legend.

• Renumbered: former Figure 1 to Figure 3, former Figure 2 to Figure 4, former Figure 3 to Figure 5.

• Supplement renumbered: former S3 to S8 Figs are now S1 to S6 Figs, in the table of contents and in the body, and the two promoted figures were removed from the supplement.

• All in-text callouts, the Supporting Information list, the S1 Protocol cross-reference and the submission figure file names were updated; no callout to the former supplementary figure numbering remains in the manuscript, the supplement or the protocol.

**ACKNOWLEDGMENT**

**Comment 24**

*Please include an acknowledgment of study participants.*

**Response:** Added.

**Changes made:**

• Acknowledgment: "We thank the people whose de-identified health records and tumor specimens made this work possible, the health care organizations that contribute data to the TriNetX Global Collaborative Network, and the specimen donors and investigators of The Cancer Genome Atlas Research Network."

**OBSERVATIONAL, COHORT, CROSS-SECTIONAL, AND CASE CONTROL STUDIES**

**Comment 25**

*Your study is observational and therefore causality cannot be inferred. Please remove language that implies causality and refer to associations instead.*

**Response:** We removed the remaining causal implications. We have also renamed the prespecified pathway framework from "causal pathways" to "mechanistic pathways" throughout, and state explicitly that the pathway labels denote hypothesized mechanisms and are not claims of established causation.

**Changes made:**

• Introduction: "supporting causal interpretation when a randomized trial is unavailable" is now "making the intended comparison explicit when a randomized trial is unavailable", followed by "Because treatment was not randomly assigned, the resulting estimates remain associations rather than randomized comparisons."

• Methods, Outcomes: the 4 pathways are now described as labels for "the hypothesized mechanisms the study was designed to discriminate among", explicitly "not claims of established causation".

• Abstract, Conclusions and Discussion, Conclusion: "improved survival" is now "lower mortality".

• Throughout the manuscript, the supplement and the protocol: "causal pathway" is now "mechanistic pathway", including the Results section heading, the Table 2 title, and the title and legend of Figure 4.

**Comment 26**

*For all observational studies, in the manuscript text, please indicate: (1) the specific hypotheses you intended to test, (2) the analytical methods by which you planned to test them, (3) the analyses you actually performed, and (4) when reported analyses differ from those that were planned, transparent explanations for differences that affect the reliability of the study's results. If a reported analysis was performed based on an interesting but unanticipated pattern in the data, please be clear that the analysis was data-driven.*

**Response:** We added a dedicated paragraph to the Methods that walks through all four points. The prespecified plan was implemented without deviation; seven analyses were added during peer review and are named individually as post hoc.

**Changes made:**

• Methods, Statistical Analysis, new paragraph "Prespecified and post hoc analyses": states the primary and secondary hypotheses, the planned analytical methods, that all planned analyses were performed as planned without deviation, and then names the 7 post-hoc analyses (overall survival without a time-window restriction; overall survival excluding drug-class crossover; the neither-agent comparator; overall survival within ICI class; the refined time-to-next-treatment endpoint; imaging-surveillance frequency; and the pathway-wide transcriptomic analysis).

• The same paragraph states that no analysis reported here was chosen on the basis of an unanticipated pattern in the data, and cross-references S1 Protocol Section 11, where the post-hoc analyses are itemized.

**CHECKLISTS**

**Comment 27**

*Thank you for providing TARGET and STROBE Checklists in Supporting information files. Please extract them from this one file and upload as separate files together with revised manuscript.*

**Response:** Both checklists have been extracted from the Supporting Information document into their own files and are uploaded separately with this revision.

**Changes made:**

• New file S1_Checklist_STROBE.docx ("S1 Checklist. STROBE Statement", items 1 to 22 across the two-part official table, with the manuscript location for each item).

• New file S2_Checklist_TARGET.docx ("S2 Checklist. The TARGET Checklist", with the location reported for each item).

• Both blocks were removed from Supplementary_Materials, including their table of contents entries.

**OTHER**

**Comment 28**

*Study Design and Data Source section - Line 144 and 148 repetitions of TARGET guideline following. Revise from Line 143 to "This study is reported in accordance with the STROBE reporting guideline [16] (S2 Checklist) and TARGET guideline for emulating a target trial [17] (S1 Checklist). Remove line 148 and revise the Checklists tables references - probably need to swap order so it is chronological.*

**Response:** Corrected. The duplicated TARGET sentence has been deleted and the surviving sentence rewritten as requested. We also swapped the checklist numbering so that it runs in the order the checklists are cited: STROBE is now S1 Checklist and TARGET is now S2 Checklist, which matches the order of mention in the revised sentence.

**Changes made:**

• Methods, Study Design and Data Source: the sentence now reads "This study is reported in accordance with the STROBE reporting guideline [16] (S1 Checklist) and the TARGET guideline for emulating a target trial [17] (S2 Checklist)."

• The duplicated sentence that opened the following paragraph ("This study is reported in accordance with the TARGET guideline for emulating a target trial ...") has been deleted.

• Checklist numbering swapped throughout the manuscript, the Supporting Information list, and the two new standalone checklist files.

**Comment 29**

*I am wondering if inclusion and exclusion criteria, and outcomes are not better suited in main body of the manuscript rather than in Supplementary text. Please consider moving it.*

**Response:** We agree, and we have set them out in the Methods themselves rather than in a table. The Study Population section now states all 7 inclusion criteria and all 10 exclusion criteria in the running text, each with its diagnostic codes, and the Outcomes section now names the primary outcome and every one of the 20 secondary outcomes under its prespecified pathway, with their codes and the ascertainment window. The supplement retains the operational code lists, whose length would otherwise interrupt the Methods narrative.

**Changes made:**

• Methods, Study Population: states all 7 inclusion criteria and all 10 exclusion criteria explicitly in the running text, each with its diagnostic codes.

• Methods, Outcomes: now names all 20 secondary outcomes individually under path A, path B, path C and path D, with their diagnostic codes, alongside the primary outcome, the 30-day landmark and the 2-year window, and states the ascertainment window applied to each outcome.

• S11 Table: a row for thyroid dysfunction (E03, E06) has been added, so that every outcome named in the Methods has a definition in the Supporting Information.

• Methods, Study Population: the cross-reference to S1 Text at the end of the exposure-window sentence has been removed, since that sentence states the window itself.

**JOURNAL REQUIREMENTS (technical checks, 7 August 2026)**

**Comment 30**

*In your Data Availability Statement, please ensure you have provided all necessary access information in complete sentences. Access information should include the name of any data repositories used as well as digital object identifiers (DOIs), accession numbers or codes, or other persistent identifiers for all relevant data. If all data has been provided within your submission files, please use the statement "All relevant data is available within the manuscript and Supporting Information files." The Data Availability Statement will appear on the published version of your manuscript.*

**Response:** The Data Availability Statement has been rewritten in complete sentences and now names each repository together with its identifiers. The same text has been entered in the submission form.

**Changes made:**

• Data Availability Statement: names the TriNetX Global Collaborative Network with its URL and the access route, the National Cancer Institute Genomic Data Commons with all 20 TCGA project accessions, and the GitHub repository with its archived Zenodo DOI; it also states that all aggregate values behind every figure and table are within the article and its Supporting Information files.

• The heading was changed from "Data Sharing Statement" to "Data Availability Statement" to match the journal's terminology.

**REVIEWERS**

**Comment 31**

*Reviewer #1: Thank you for the detailed response to my comments. The authors have adequately addressed all of my previous comments. I have no further comments or revisions to suggest. Reviewer #2: I am satisfied with the revision. Reviewer #3: The authors have addressed all my concerns sufficiently.*

**Response:** We thank all three reviewers for their careful reading across the two rounds of review. No further changes were requested, and the scientific content, analyses and numerical results of the manuscript are unchanged in this revision; the revisions above are editorial and presentational.

**Changes made:**

• No change was required.
