# Supplementary material for: Selective serotonin reuptake inhibitors and immune checkpoint blockade in solid tumors: A target trial emulation with pan-cancer transcriptomic analyses
Source: PLoS Med. 2026 Sep 29;23(9):e1005262. doi: 10.1371/journal.pmed.1005262 (PMC13645231; doi:10.1371/journal.pmed.1005262)
Supplement: Supplementary file 27 [file pmed.1005262.s033.docx]

**Response to the Editors**

**Manuscript PMEDICINE-D-26-00932R3**

*Selective serotonin reuptake inhibitors and immune checkpoint blockade in solid tumors: A target trial emulation with pan-cancer transcriptomic analyses*

We are grateful to the Associate Editor for this close reading, which has sharpened several statements that matter to how the paper will be read. Below we quote each of the 19 remaining requests verbatim, followed by our response and the specific changes made. All changes are marked in the accompanying marked-up manuscript. No analysis was rerun and no result changed: every estimate, confidence interval, p value and event count is as in the previous revision. One count that had not been reported before is added in answer to Comment 15. Requests 2, 3, 5, 9, 10 and 11 all add material to the abstract, so to stay inside the 500-word limit we have written the new text as compactly as we could and tightened a small number of existing phrases; no result has been removed and the abstract is now 499 words.

**REQUESTS FROM EDITORS**

**Comment 1**

*Please revise the title to: Selective serotonin reuptake inhibitors and immune checkpoint blockade in solid tumors: A target trial emulation with pan-cancer transcriptomic analyses [or analysis, singular]*

**Response:** Thank you for supplying the wording. We have adopted the title exactly as you proposed and taken the plural form "analyses", since the transcriptomic work comprises several distinct analyses: the per-cancer correlation of SLC6A4 expression with immune function scores, the immune phenotype classification, the correlation with six immune cell-type scores, a random-effects survival meta-analysis across 18 cancer types, the extension to the 20-gene serotonin pathway, and the organ-level mapping. The new title names the exposure and the population directly, and at 152 characters it sits within the 200-character limit.

**Changes made:**

• Title page: "Serotonin transporter inhibition and immune checkpoint blockade outcomes: a target trial emulation with pan-cancer transcriptomic validation" now reads "Selective serotonin reuptake inhibitors and immune checkpoint blockade in solid tumors: A target trial emulation with pan-cancer transcriptomic analyses".

• Supplementary Materials, title page: the same title has been updated.

• The short title, "SSRI use and immune checkpoint blockade outcomes", is unchanged.

**Comment 2**

*line 33 of Abstract, instead of "We compared..." you need to add "Using a target trial emulation framework, we compared..." [or "In a target trial emulation, we compared..."*

**Response:** We are glad to adopt this. Naming the design where the reader first meets the comparison makes the framework explicit from the outset, and we have used your wording verbatim.

**Changes made:**

• Abstract, Background: "We compared outcomes between users of SSRIs and users of benzodiazepines (BZDs) among patients with a solid tumor and depression or anxiety who were receiving an ICI" now reads "Using a target trial emulation framework, we compared outcomes between SSRI users and benzodiazepine (BZD) users among patients with a solid tumor and depression or anxiety who were receiving an ICI."

**Comment 3**

*line 35: if this statement "and examined SLC6A4 expression and immune suppression...." is not from the same patients as the TTE, then they need to so state. e.g. "and examined SLC6A4 expression and immune suppression across cancers in The Cancer Genome Atlas."*

**Response:** They are indeed different patients, and we agree this is worth stating rather than leaving to inference. The Cancer Genome Atlas is an entirely separate population: no patient contributes to both the target trial emulation and the transcriptomic analyses, and the two datasets share no individuals. Following your suggestion we have named TCGA in the sentence and said explicitly that it is a different set of patients. The Discussion already carried this qualification; the abstract now carries it as well, and the limitation sentence added under Comment 11 reinforces it.

**Changes made:**

• Abstract, Background, final sentence: "and examined SLC6A4 expression and immune suppression across human cancers" now reads "Separately, in The Cancer Genome Atlas (TCGA), which covers a different set of patients, we examined how SLC6A4 expression related to tumor immune-cell infiltration across cancers."

• Author summary, third finding bullet: "in tumor tissue from more than 8,000 cancers" now reads "in tumor tissue from more than 8,000 cancers in a separate group of patients". The author summary now carries the same qualification as the abstract.

**Comment 4**

*also--it's not immune suppression if you are just looking at elevated PD-L1, for e.g.*

**Response:** This is a distinction we are glad to draw more precisely, and we have carried it through the whole paper. What the TCGA analyses measure are transcriptomic signatures of immune-cell infiltration and T-cell inflammation: the T-cell Inflamed Gene Expression Profile, the Cytotoxic Score, a three-way immune phenotype classification (Inflamed, Excluded, Desert), and Spearman correlations with six immune cell-type scores. No PD-L1 measurement enters the analysis at any point. "Immune suppression" therefore names a mechanism where the data support only an association, so at every occurrence it has been replaced by wording that names the measurement, and the phrase now appears nowhere in the manuscript. Applying the same reasoning, we then looked for the idea rather than the phrase and tightened three further statements where a construct stood in for a measurement: "immune exclusion" and "immune evasion" in the manuscript, and a color key in the S5 Fig legend that read a negative correlation as "immune-suppressive". Each now states what was measured rather than carrying a qualifier.

**Changes made:**

• Abstract, Background: "immune suppression" replaced, see Comment 3.

• Abstract, Methods and Findings: "concordance between SLC6A4-associated immune suppression and the observed pattern of organ-specific irAEs" now reads "this organ-level pattern matched that of organ-specific irAEs".

• Introduction, closing hypothesis: "the tissues where SLC6A4 expression tracks most closely with immune suppression" now reads "... with reduced immune-cell infiltration".

• Methods, TCGA subsection: "whether the serotonin transporter gene SLC6A4 associates with immune suppression across human cancers" now reads "... associates with the extent of immune-cell infiltration across human cancers".

• Results, Organ-Level Cross-Validation: "concordance between transcriptomic immune suppression and clinical immune activation" now reads "concordance between reduced tumor immune-cell infiltration in the transcriptomic data and clinical immune activation", and "one of the strongest SLC6A4-immune suppression signals" now reads "one of the strongest inverse SLC6A4-immune associations".

• Discussion: "whether SLC6A4-mediated immune suppression is tissue-specific" now reads "whether the inverse association between SLC6A4 expression and tumor immune-cell infiltration is tissue-specific", and "the thyroid, where SLC6A4-immune suppression was strongest" now reads "the thyroid, where the inverse SLC6A4-immune association was strongest".

• Discussion, closing paragraph: "Transcriptomic evidence for SLC6A4-mediated immune suppression across human cancers" now reads "Transcriptomic evidence that SLC6A4 expression is inversely associated with tumor immune-cell infiltration across human cancers".

• Results, serotonin-pathway paragraph: "indicating that immune exclusion tracks specifically with the serotonin-handling transporter" now reads "indicating that the inverse association with immune signatures tracks specifically with the serotonin-handling transporter". The sentence describes the gene-by-signature correlation matrix rather than the Excluded immune phenotype, so it now names the association.

• Discussion: "and associated with the Immune Desert phenotype, supporting a pan-cancer role for SERT in immune evasion" now reads "and associated with the Immune Desert phenotype, a pan-cancer pattern rather than a single-tumor observation". The mechanistic reading is left to the preclinical work we cite rather than being drawn from a cross-sectional correlation.

• Supplementary Materials, S5 Fig legend: "Blue = negative (immune-suppressive); red = positive" now reads "Blue = negative correlation; red = positive", and the same immune-exclusion wording was narrowed as in the Results. The color key now describes the sign of the correlation rather than interpreting it.

• Results section heading: see Comment 18.

**Comment 5**

*and did patients really receive the antidepressant for the first time on the first day of ICI infusion? an active prescription for an SSRI or a benzodiazepine overlapping the index date or starting within the preceding 90 days; (line 200)*

**Response:** They did not, and we agree the abstract should say so plainly. Eligibility required an active SSRI or benzodiazepine prescription overlapping the index date or starting within the preceding 90 days, and the pre-specified exposure window runs from 90 days before to 14 days after the index date (S1 Protocol, Section 5). Patients were therefore new users of immune checkpoint inhibitor therapy, since the index date is the first ICI administration, but not necessarily new users of the psychotropic agent: many will have been taking it before the first infusion. The abstract now states the exposure window, the Methods say so explicitly, and the author summary uses "taking" rather than "starting" so the lay-facing text carries the same meaning.

**Changes made:**

• Abstract, Methods and Findings: "comparing initiation of an SSRI with initiation of a BZD at first ICI administration" now reads "comparing SSRI with BZD treatment at first ICI administration".

• Abstract, Methods and Findings: new sentence, "Exposure required an active SSRI or BZD prescription overlapping the index date or starting within the preceding 90 days, not necessarily on that date."

• Methods, Study Population: new closing sentence, "Exposure was therefore ascertained from an active prescription in the window from 90 days before to 14 days after the index date: patients were new users of ICI therapy, and their psychotropic treatment could already have been in place before the first ICI infusion rather than starting on that day."

• Methods, Statistical Analysis: "the intention-to-treat-analogue effect of initiating an SSRI versus a benzodiazepine at ICI initiation" now reads "the intention-to-treat-analogue effect of SSRI versus benzodiazepine treatment at ICI initiation".

• Author summary, first finding bullet: "we compared 1,567 people starting an SSRI with 1,567 people starting a benzodiazepine" now reads "we compared 1,567 people taking an SSRI with 1,567 people taking a benzodiazepine", for the same reason.

• Supplementary Materials, S1 Text, inclusion criterion 5: "Active prescription of SSRI or BZD overlapping with or within 90 days before index date" now reads "Active prescription of SSRI or BZD within 90 days before to 14 days after index date", matching S1 Protocol and the Methods sentence above.

**Comment 6**

*Not sure if “transcriptomic validation” term is correct here. You are looking more at association.*

**Response:** We agree that association is the accurate description. The TCGA work estimates associations between SLC6A4 expression and immune features of the tumor microenvironment; being a different population and a cross-sectional design, it can support the clinical finding but not validate it. "Transcriptomic validation" has therefore been replaced by "transcriptomic analyses" wherever it appeared, including in the title. We have kept the term "organ-level cross-validation" for the one comparison in which the organ ranking derived from TCGA is set against the organ ranking of clinical irAEs, since it names a cross-comparison of two independent datasets rather than a claim about the clinical result, and we will change that term as well if you prefer. The pre-specified S1 Protocol keeps its original wording, as the pre-study document is the record of what was planned.

**Changes made:**

• Title: "pan-cancer transcriptomic validation" now "pan-cancer transcriptomic analyses", see request 1.

• Abstract, Methods and Findings: "Transcriptomic validation used The Cancer Genome Atlas" now reads "Transcriptomic analyses used TCGA".

• Methods section heading: see Comment 14.

• Data Availability Statement: "All analysis code for the transcriptomic validation" now reads "All analysis code for the transcriptomic analyses".

• Figure 5 legend: "TCGA pan-cancer transcriptomic validation and organ-level cross-validation" now reads "TCGA pan-cancer transcriptomic analyses and organ-level cross-validation".

• Supplementary Materials, contents list and body: the heading "S1 Text (continued). Transcriptomic Validation: TCGA Pan-Cancer Analysis" now reads "S1 Text (continued). TCGA Pan-Cancer Transcriptomic Analyses".

**Comment 7**

*line 47: 2-year mortality--all cause or cancer specific. You should state that.*

**Response:** All-cause. The primary outcome is overall survival, that is, death from any cause; cause of death is not recorded in the network, so a cancer-specific analysis is not available to us. The Methods already frame the pre-specified primary hypothesis as "2-year all-cause mortality", and the abstract, the Introduction and the Results now use the same term, so the outcome is identified the same way wherever it appears.

**Changes made:**

• Abstract, Methods and Findings: "SSRI use was associated with lower 2-year mortality" now reads "... lower 2-year all-cause mortality".

• Results, Study Cohort and Primary Outcome: "SSRI use was associated with significantly lower 2-year mortality compared with BZD use" now reads "SSRI use was associated with significantly lower 2-year all-cause mortality compared with BZD use". The primary outcome row of Table 2 is labeled "All-cause mortality", so the Results text now matches it.

• Introduction, closing hypothesis: see Comment 12.

**Comment 8**

*lines 51, 52--you need to say in which groups these outcomes were less frequent and not leave it open to interpretation.*

**Response:** A fair point, and the direction is now named. The metastasis sentence says which group the events were less frequent in. The irAE sentence in the same passage already read "more frequent with SSRI use", so it stands as it was.

**Changes made:**

• Abstract, Methods and Findings: "Newly coded distant metastases were less frequent (HR 0.629 ...)" now reads "Newly coded distant metastases were less frequent in the SSRI group (HR 0.629 ...)".

• Results, path A: "New distant metastases were significantly less frequent in patients without baseline metastases" now reads "New distant metastases were significantly less frequent with SSRI use among patients without baseline metastases". The Results sentence named the restricted population but not the comparator, so it carried the same ambiguity you raise here.

• Abstract, Methods and Findings: the negative control outcomes are now named, see Comment 9.

**Comment 9**

*"3 negative control outcomes" is not very informative*

**Response:** Agreed, and they are now named. The 3 pre-specified negative control outcomes are nephrolithiasis, cholelithiasis, and cataract, chosen as conditions with no biological reason to differ between SSRI and benzodiazepine users and therefore usable as bias indicators. Naming them lets a reader judge whether they are plausible controls rather than take the count on trust.

**Changes made:**

• Abstract, Methods and Findings: "3 negative control outcomes showed null associations (all p>0.76)" now reads "the 3 prespecified negative controls (nephrolithiasis, cholelithiasis, cataract) showed null associations (all p>0.76)".

**Comment 10**

*line 57:"performance status is not recorded in the network," what does this mean? clarify for general reader*

**Response:** Performance status is a clinician's rating of how well a patient can carry out ordinary daily activities, on a scale running from fully active to completely bedbound. It is among the strongest predictors of survival in cancer and is used routinely to decide who is fit enough for systemic treatment. In this network it exists in oncology notes as free text rather than as a structured code, so it cannot be extracted and could not be included among the matching covariates. Following your suggestion the abstract now explains the term rather than merely naming it, which should make the limitation legible to a general reader.

**Changes made:**

• Abstract, Methods and Findings: "performance status is not recorded in the network" now reads "performance status, a clinician's rating of everyday functional ability that strongly predicts cancer survival, is not recorded in the network".

**Comment 11**

*also you should add a comment on the limitations of the TCGA data.*

**Response:** A useful addition, and we have made it. The abstract now closes on the limitations of the transcriptomic data themselves, alongside the clinical limitation already there. The three we regard as material are that the TCGA data are cross-sectional, so they cannot establish the direction of any relationship; that they are bulk tumor RNA-seq, so SLC6A4 expression cannot be assigned to a particular cell type; and that they come from patients who were not treated with immune checkpoint inhibitors, so the analyses speak to biological plausibility rather than showing that SSRIs change the tumor microenvironment. The Discussion develops the same points at greater length.

**Changes made:**

• Abstract, Methods and Findings, new closing sentence: "The TCGA analyses are cross-sectional and use bulk tumor tissue from patients not treated with ICIs, so they cannot localize SLC6A4 to particular cells or show that SSRIs alter the tumor microenvironment."

**Comment 12**

*Line 159: all cause or cancer specific?*

**Response:** All-cause here as well. The Introduction now uses the same term as the abstract and the Results, so the primary outcome is described consistently at every mention.

**Changes made:**

• Introduction, closing hypothesis: "is associated with lower 2-year mortality" now reads "is associated with lower 2-year all-cause mortality".

**Comment 13**

*Line 161 not immune suppression*

**Response:** Agreed, and the same change has been made here. The sentence now refers to the tissues where SLC6A4 expression tracks with reduced immune-cell infiltration, which is what the correlation shows.

**Changes made:**

• Introduction, closing hypothesis: "in the tissues where SLC6A4 expression tracks most closely with immune suppression" now reads "... with reduced immune-cell infiltration".

**Comment 14**

*Line 304 not validation*

**Response:** Agreed. The heading now reads "TCGA Pan-Cancer Transcriptomic Analyses", for the reason set out under Comment 6.

**Changes made:**

• Methods, section heading: "TCGA Pan-Cancer Transcriptomic Validation" now reads "TCGA Pan-Cancer Transcriptomic Analyses".

**Comment 15**

*Line 306 are that 8272 samples from 8272 patients*

**Response:** Not quite, and the distinction is worth reporting. The 8,272 tumor samples come from 8,172 individual patients: 100 patients contributed more than one tumor sample, and no patient appears in more than one tumor type. We re-derived this by counting distinct TCGA patient barcodes in the sample-level gene set enrichment output that underlies Figure 5A, which holds 8,272 rows and 8,172 unique patient identifiers, the duplicated patients falling in nine projects (BRCA 16, COAD 23, LUAD 23, LUSC 10, KIRC 8, UCEC 8, BLCA 6, PRAD 5, PAAD 1). Both counts are now reported in the manuscript and in the supplement.

**Changes made:**

• Abstract, Methods and Findings: "TCGA; 8,272 samples, 20 tumor types" now reads "TCGA (8,272 tumor samples from 8,172 patients, 20 tumor types)".

• Methods, TCGA subsection: "we analyzed RNA-seq data from 8,272 samples across 20 solid tumor types" now reads "we analyzed RNA-seq data from 8,272 tumor samples contributed by 8,172 patients across 20 solid tumor types in The Cancer Genome Atlas (TCGA); 100 patients contributed more than one tumor sample, and no patient appeared in more than one tumor type".

• Supplementary Materials, S1 Text: new sentence, "In total, 8,272 tumor samples contributed by 8,172 patients were analyzed; 100 patients contributed more than one tumor sample, and no patient appeared in more than one tumor type."

**Comment 16**

*Line 323 Us of AI tools is separate section*

**Response:** Agreed, and it now stands on its own. The statement is the final subsection of the Methods, with its own heading at the same level as the other subsections, so it reads as a declaration about the study as a whole rather than as part of the transcriptomic methods. The text of the statement is unchanged.

**Changes made:**

• Methods: new subsection heading "Use of Artificial Intelligence Tools", placed after "TCGA Pan-Cancer Transcriptomic Analyses" as the last subsection of the Methods.

• Methods: the run-in label "Use of artificial intelligence tools." has been removed from the start of the paragraph, which now begins "Claude (Anthropic) was used, through the Claude Code interface ...". No other wording in the statement has changed.

**Comment 17**

*Line 385: the sentence “ Findings were consistent across the analyses requested during revision” does not add much as reader doesn’t know what is from original and what from revised MS*

**Response:** We take the point, and the sentence is gone. It asked the reader to keep track of which analyses belonged to which round, which the published article gives them no way to do. The paragraph now opens directly on the first sensitivity analysis. The provenance a reader does need is already in one place, the "Prespecified and post hoc analyses" paragraph of the Methods, which names the seven post hoc analyses individually.

**Changes made:**

• Results, Subgroup and Sensitivity Analyses: the opening sentence "Findings were consistent across the analyses requested during revision." has been deleted. The paragraph now begins "Overall survival without any time-window restriction yielded a similar estimate ...".

**Comment 18**

*Line 399 : Not sure if this heading makes sense, I would rather say with “Immune response”*

**Response:** "Immune response" is the better fit and we have used it. The heading now reads "SLC6A4 Expression and Tumor Immune Response Across Human Cancers", which describes what the section reports and also carries through the wording point you raise under Comment 4.

**Changes made:**

• Results, section heading: "SLC6A4 Expression and Immune Suppression Across Human Cancers" now reads "SLC6A4 Expression and Tumor Immune Response Across Human Cancers".

**Comment 19**

*Line 580 DOI URL is not working, please correct*

**Response:** Thank you for checking this. The identifier itself resolves: https://doi.org/10.5281/zenodo.21882308 returns the Zenodo record, which we confirmed again on 21 August 2026 together with the deposited archive and the linked GitHub repository. What the manuscript printed was the bare identifier, "DOI 10.5281/zenodo.21882308", with no URL and no hyperlink, so there was nothing on the page to follow. It is now written in resolvable URL form, matching how the TriNetX, Genomic Data Commons and GitHub addresses appear in the same statement; we re-checked those three on the same date and all resolve.

**Changes made:**

• Data Availability Statement: "is permanently archived in Zenodo (DOI 10.5281/zenodo.21882308)" now reads "is permanently archived in Zenodo (https://doi.org/10.5281/zenodo.21882308)".
