# Supplementary material for: Selective serotonin reuptake inhibitors and immune checkpoint blockade in solid tumors: A target trial emulation with pan-cancer transcriptomic analyses
Source: PLoS Med. 2026 Sep 29;23(9):e1005262. doi: 10.1371/journal.pmed.1005262 (PMC13645231; doi:10.1371/journal.pmed.1005262)
Supplement: Peer review history

# Editor report: Decision Letter 0

**DOI:** 10.1371/journal.pmed.1005262.r001

**Authors:** Helen Howard

24 Mar 2026

Dear Dr Chen,

Thank you for submitting your manuscript entitled "Serotonin transporter inhibition enhances immune checkpoint blockade: target trial emulation with pan-cancer transcriptomic validation" for consideration by PLOS Medicine.

Your manuscript has now been evaluated by the PLOS Medicine editorial staff, and I am writing to let you know that we would like to send your submission out for external peer review.

However, before we can send your manuscript to reviewers, we need you to complete your submission by providing the metadata that is required for full assessment. To this end, please login to Editorial Manager where you will find the paper in the 'Submissions Needing Revisions' folder on your homepage. Please click 'Revise Submission' from the Action Links and complete all additional questions in the submission questionnaire.

For clinical studies, please upload a copy of your trial study protocol as a supporting information file. The study protocol should be the version submitted for approval to the institutional review board or ethics committee, should include any amendments to the study protocol, as well as the date of their approval by the institutional review or ethics committee. Please also detail any deviations from the study protocol in the Methods section of your manuscript. The editors will consider the protocol and study conduct prior to a final decision for external review.

Please re-submit your manuscript within two working days, i.e. by Mar 26 2026 11:59PM.

Login to Editorial Manager here: https://www.editorialmanager.com/pmedicine

Once your full submission is complete, your paper will undergo a series of checks in preparation for peer review. Once your manuscript has passed all checks it will be sent out for review.

Feel free to email us at plosmedicine@plos.org if you have any queries relating to your submission.

Kind regards,

Helen Howard

PLOS Medicine

# Peer review: Decision Letter 1

**DOI:** 10.1371/journal.pmed.1005262.r002

**Authors:** Katarzyna Starowicz

19 Jun 2026

Dear Dr Chen,

Many thanks for submitting your manuscript "Serotonin transporter inhibition enhances immune checkpoint blockade: target trial emulation with pan-cancer transcriptomic validation" (PMEDICINE-D-26-00932R1) to PLOS Medicine. The paper has been reviewed by subject experts and a statistician; their comments are included below and can also be accessed here: [LINK]

After discussing the paper with the editorial team and an academic editor with relevant expertise, I'm pleased to invite you to revise the paper in response to the reviewers' comments. As you will see, the reviewers and academic editor finds your study of potential interest however, the have raise some concerns around methodology and analysis. A We plan to send the revised paper to some or all of the original reviewers, and we cannot provide any guarantees at this stage regarding publication.

Please note, after reviewers comments there are listed editorial general requests and study-type specific requests at the end of this letter. You must provide point-by-point response to each of them. Some of them might not be applicable to your study, in that case please respond "not applicable". Not providing this in form of rebuttal will significantly delay your manuscript.

In addition to these revisions, you may need to complete some formatting changes, which you will receive in a follow up email. A member of our team will be in touch with a set of requests shortly. If you do not receive a separate email within a few days, please assume that checks have been completed, and no additional changes are required.

When you upload your revision, please include a point-by-point response that addresses all of the reviewer and editorial points, indicating the changes made in the manuscript and either an excerpt of the revised text or the location (eg: page and line number) where each change can be found. Please also be sure to check the general editorial comments at the end of this letter and include these in your point-by-point response. When you resubmit your paper, please include a clean version of the paper as the main article file and a version with changes tracked as a marked-up manuscript. It may also be helpful to check the guidelines for revised papers at http://journals.plos.org/plosmedicine/s/revising-your-manuscript for any that apply to your paper.

We ask that you submit your revision by Jul 10 2026 11:59PM. However, if this deadline is not feasible, please contact me by email, and we can discuss a suitable alternative.

Don't hesitate to contact me directly with any questions (kstarowicz@plos.org).

Best regards,

Katarzyna

Katarzyna Starowicz, PhD

Associate Editor

PLOS Medicine

kstarowicz@plos.org

-----------------------------------------------------------

Comments from the academic editor:

Overall, I found this to be a novel and clinically relevant study. After reading the manuscript and the reviews, I also felt that the main findings are of interest.

I agree with Reviewer 2 that further clarification is needed regarding the interpretation of the metastasis findings, the relationship between the clinical findings and the TCGA analyses, and the consistency of the observed associations across ICI classes.

The manuscript reports a survival benefit, a reduction in new metastases, a thyroid-predominant irAE signal, consistent findings across individual SSRIs, and negative control analyses without significant associations. These findings all point in a similar direction and are discussed within a common biological framework. I believe the distinction between the observed clinical associations and the proposed biological mechanism should be stated as clearly as possible.

One of the strengths of the study is the direct comparison between SSRI and BZD users. However, I believe the discussion would benefit from further consideration of how this comparison should be interpreted clinically, particularly given the different clinical circumstances in which these medications may be prescribed. Additional discussion of this point would help readers better understand the strengths and limitations of the comparison.

-----------------------------------------------------------

Comments from the reviewers:

Reviewer #1: The manuscript titled "Serotonin transporter inhibition enhances immune checkpoint blockade: target trial emulation with pan-cancer transcriptomic validation" investigates the clinical outcome between SSRI and benzodiazepine (BZD) users among ICI-treated solid tumor patients with depression or anxiety using target trial emulation method. This aims to evaluate if the SSRI can be repurposed to as an ICI adjuvant. The study is well designed, and the statistical methods used are sound and robust. I have two minor comments.

The concurrent medication of SSRI and BZD in method study population was not clear. The concurrent medication of SSRI or BZD was mutually exclusive at baseline (prior to the ICI) or throughout the evaluation period (2-year window)?

The in the method outcome section, the follow-up was restricted to 2-year window. All participants were censored at 730 days (2-year window), why? Perhaps a sensitivity analysis without restricting time-window would be preferable.

Reviewer #2: This manuscript reports a TriNetX target-trial emulation comparing SSRI-only vs benzodiazepine (BZD)-only use among immune checkpoint inhibitor (ICI)-treated solid tumor patients with depression/anxiety, with complementary TCGA analyses linking SLC6A4 expression to immune suppression across cancers. The work is potentially practice-relevant; however, several key claims, particularly those connecting reduced metastases and TCGA correlations to "direct antitumor immune enhancement", would benefit from additional analyses and clearer endpoint framing.

Major points

*The primary endpoint is 2-year overall survival, with an additional "new distant metastasis" analysis in those without baseline metastases. What does SSRI use predict for progression-free survival (PFS)? If PFS cannot be directly measured in TriNetX, please provide the closest available, prespecified proxies (e.g., time-to-next-treatment or other progression surrogates already defined in your outcomes framework) and clarify how they relate to the metastasis endpoint and overall survival.

*On Page 23, the manuscript states that reduced metastatic progression "supports direct antitumor immune enhancement." Many factors influence metastasis detection and documentation (e.g., baseline staging intensity, surveillance imaging frequency, etc.). How do the authors rule out detection bias or residual confounding as explanations for fewer coded "new metastases"?

*The TCGA results show inverse correlations between SLC6A4 and T-cell inflammation in many tumor types and organ-level concordance with clinical irAE patterns. Does the observed SLC6A4-immune suppression signal transform toward SSRI-treated patients (i.e., does SSRI use convert immune-desert tumors toward inflamed phenotypes)?

*The included ICI therapies span multiple classes. How heterogeneous are treatment regimens in practice, and how might this affect observed associations? Are SSRI-associated benefits similar or different across ICI classes? Please provide evidence.

Minor points

*Are there sensitivity analyses comparing SSRI users to patients with depression/anxiety on neither SSRI nor BZD?

Reviewer #3: In their work Chen et al analysed 3134 matched patients from a huge database to compare ICI outcome of SSRI intake verse benzodiazepines. I con gratulate the authors to this tremendous effort finding the patients and a matching control cohort.

The manuscript is well written in regards of the research question, data collection, results and discussion.

I have only one minor point: for somebody not totally in the field the introduction is a little bit too short and especially the role and why an analysis on SLC6A4 was undertaken, and e.g. downstream signalling, is not conclusive for a reader. This must be more in depth elaborated.

Any attachments provided with reviews can be seen via the following link: [LINK]

--------------------------------------------------------- ---

General editorial requests:

(Note: not all will apply to your paper, but please check each item carefully)

*We ask every co-author listed on the manuscript to fill in a contributing author statement, making sure to declare all competing interests. If any of the co-authors have not filled in the statement, we will remind them to do so when the paper is revised. If all statements are not completed in a timely fashion this could hold up the re-review process. If new competing interests are declared later in the revision process, this may also hold up the submission. Should there be a problem getting one of your co-authors to fill in a statement we will be in contact. Please do not add or remove authors without first discussing this with the handling editor. You can see our competing interests policy here: http://journals.plos.org/plosmedicine/s/competing-interests.

*Please upload any figures associated with your paper as individual TIF or EPS files with 300dpi resolution at resubmission; please read our figure guidelines for more information on our requirements: http://journals.plos.org/plosmedicine/s/figures. While revising your submission, we strongly recommend that you use PLOS's NAAS tool (https://ngplosjournals.pagemajik.ai/artanalysis) to test your figure files. NAAS can convert your figure files to the TIFF file type and meet basic requirements (such as print size, resolution), or provide you with a report on issues that do not meet our requirements and that NAAS cannot fix.

After uploading your figures to PLOS's NAAS tool - https://ngplosjournals.pagemajik.ai/artanalysis, NAAS will process the files provided and display the results in the "Uploaded Files" section of the page as the processing is complete.

If the uploaded figures meet our requirements (or NAAS is able to fix the files to meet our requirements), the figure will be marked as "fixed" above. If NAAS is unable to fix the files, a red "failed" label will appear above.

When NAAS has confirmed that the figure files meet our requirements, please download the file via the download option, and include these NAAS processed figure files when submitting your revised manuscript.

*Please ensure that the paper adheres to the PLOS Data Availability Policy (see http://journals.plos.org/plosmedicine/s/data-availability), which requires that all data underlying the study's findings be provided in a repository or as Supporting Information. For data residing with a third party, authors are required to provide instructions with contact information (web or email address) for obtaining the data. Please note that a study author cannot be the contact person for the data. PLOS journals do not allow statements supported by "data not shown" or "unpublished results." For such statements, authors must provide supporting data or cite public sources that include it.

*We expect all researchers with submissions to PLOS in which author-generated code underpins the findings in the manuscript to make all author-generated code available without restrictions upon publication of the work. In cases where code is central to the manuscript, we require the code to be made available as a condition of publication. Authors are responsible for ensuring that the code is reusable and well documented. Please make any custom code available, either as part of your data deposition or as a supplementary file. Please add a sentence to your data availability statement regarding any code used in the study, e.g. "The code used in the analysis is available from Github [URL] and archived in Zenodo [DOI link]" Please review our guidelines at https://journals.plos.org/plosmedicine/s/materials-software-and-code-sharing and ensure that your code is shared in a way that follows best practice and facilitates reproducibility and reuse. Because Github depositions can be readily changed or deleted, we encourage you to make a permanent DOI'd copy (e.g. in Zenodo) and provide the URL.

*Please ensure that the study is reported according to the TRAGET guideline and include the completed checklist as Supporting Information. When completing the checklist, please use section and paragraph numbers, rather than page numbers. Please add the following statement, or similar, to the Methods: "This study is reported as per TARGET guideline (S1 Checklist)."

FORMATTING - GENERAL

*Abstract: Please structure your abstract using the PLOS Medicine headings (Background, Methods and Findings, Conclusions). Please combine the Methods and Findings sections into one section.

*At this stage, we ask that you include a short, non-technical Author Summary of your research to make findings accessible to a wide audience that includes both scientists and non-scientists. The Author Summary should immediately follow the Abstract in your revised manuscript. This text is subject to editorial change and should be distinct from the scientific abstract. Ideally each sub-heading should contain 2-3 single sentence, concise bullet points containing the most salient points from your study. In the final bullet point of 'What Do These Findings Mean?', please include the main limitations of the study in non-technical language. Please see our author guidelines for more information: https://journals.plos.org/plosmedicine/s/revising-your-manuscript#loc-author-summary.

*Please express the main results with 95% CIs as well as p values. When reporting p values please report as p<0.001 and where higher as the exact p value p=0.002, for example. Throughout, suggest reporting statistical information as follows to improve clarity for the reader "22% (95% CI [13%,28%]; p</=)". Please be sure to define all numerical values at first use.

*Please include page numbers and line numbers in the manuscript file. Use continuous line numbers (do not restart the numbering on each page).

*Please cite the reference numbers in square brackets. Citations should precede punctuation.

FIGURES AND TABLES

*Please provide titles and legends for all figures and tables (including those in Supporting Information files).

*Please define all abbreviations used in each figure/table (including those in Supporting Information files).

*Please consider avoiding the use of red and green in order to make your figure more accessible to those with color blindness.

SUPPLEMENTARY MATERIAL

*Please note that supplementary material will be posted as supplied by the authors. Therefore, please amend it according to the relevant comments outlined here.

*Please cite your Supporting Information as outlined here: https://journals.plos.org/plosmedicine/s/supporting-information

REFERENCES

*PLOS uses the numbered citation (citation-sequence) method and first six authors, et al.

*Please ensure that journal name abbreviations match those found in the National Center for Biotechnology Information (NCBI) databases (http://www.ncbi.nlm.nih.gov/nlmcatalog/journals), and are appropriately formatted and capitalised.

*Where website addresses are cited, please include the complete URL and specify the date of access (e.g. [accessed: 12/06/2023]).

*Please also see https://journals.plos.org/plosmedicine/s/submission-guidelines#loc-references for further details on reference formatting.

RESULTS

* In your study you have performed the expression analysis and you choose one gene. What about all of the other genes that potentially modulate its signaling? Please explain and provide better background and justification for looking at it. If you have transcriptomic data, why is it limited to just one gene?

STUDY TYPE-SPECIFIC REQUESTS - TTE

* Please confirm that the study adheres to the TARGET guidelines for reporting emulation of a target trial.

* Please complete the TARGET checklist with paragraph/sections references indicating where each item is addressed. Please include the completed checklist with the supporting information.

*Please rewrite the abstract to meet the criteria for an abstract for a TTE--describe what that is, the primary outcomes etc.

*Please provide protocol and clarify if it was prespecified. Also you do per protocol analysis rather than ITT - explain why.

* Please include a clearly specified hypothetical target trial protocol in the Methods detailing eligibility criteria, intervention strategies, treatment assignment, follow-up period, outcome definitions, causal contrast(s) (estimands), assumptions, and analysis plan. Please describe how each of these components is emulated using the observational data (including mapping decisions or limitations). The mapping should include discussion of unmeasured variables, timing decisions (e.g., “time zero”), potential selection or measurement biases, and sensitivity or robustness of analyses.

* It might be beneficial if you explain in the Introduction what a TTE is and how it can be used in the absence of clinical trial data so that someone unfamiliar with it can better appreciate the use.

# Peer review: Decision Letter 2

**DOI:** 10.1371/journal.pmed.1005262.r003

**Authors:** Katarzyna Starowicz

6 Aug 2026

Dear Dr. Lee,

Thank you very much for re-submitting your manuscript "Serotonin transporter inhibition and immune checkpoint blockade outcomes: a target trial emulation with pan-cancer transcriptomic validation" (PMEDICINE-D-26-00932R2) for review by PLOS Medicine.

I have discussed the paper with my colleagues and the academic editor and it was also seen again by three reviewers. I am pleased to say that provided the remaining editorial and production issues are dealt with we are planning to accept the paper for publication in the journal.

The remaining issues that need to be addressed are listed at the end of this email. Any accompanying reviewer attachments can be seen via the link below. Please take these into account before resubmitting your manuscript:

[LINK]

***Please note while forming your response, if your article is accepted, you may have the opportunity to make the peer review history publicly available. The record will include editor decision letters (with reviews) and your responses to reviewer comments. If eligible, we will contact you to opt in or out.***

In revising the manuscript for further consideration here, please ensure you address the specific points made by each reviewer and the editors. In your rebuttal letter you should indicate your response to the reviewers' and editors' comments and the changes you have made in the manuscript. Please submit a clean version of the paper as the main article file. A version with changes marked must also be uploaded as a marked up manuscript file.

Please also check the guidelines for revised papers at http://journals.plos.org/plosmedicine/s/revising-your-manuscript for any that apply to your paper. If you haven't already, we ask that you provide a short, non-technical Author Summary of your research to make findings accessible to a wide audience that includes both scientists and non-scientists. The Author Summary should immediately follow the Abstract in your revised manuscript. This text is subject to editorial change and should be distinct from the scientific abstract.

In addition to these revisions, you may need to complete some formatting changes, which you will receive in a follow up email. A member of our team will be in touch with a set of requests shortly. If you do not receive a separate email within a few days, please assume that checks have been completed, and no additional changes are required.

We expect to receive your revised manuscript within 1 week. Please email us (plosmedicine@plos.org) if you have any questions or concerns.

We ask every co-author listed on the manuscript to fill in a contributing author statement. If any of the co-authors have not filled in the statement, we will remind them to do so when the paper is revised. If all statements are not completed in a timely fashion this could hold up the re-review process. Should there be a problem getting one of your co-authors to fill in a statement we will be in contact. YOU MUST NOT ADD OR REMOVE AUTHORS UNLESS YOU HAVE ALERTED THE EDITOR HANDLING THE MANUSCRIPT TO THE CHANGE AND THEY SPECIFICALLY HAVE AGREED TO IT.

Please ensure that the paper adheres to the PLOS Data Availability Policy (see http://journals.plos.org/plosmedicine/s/data-availability), which requires that all data underlying the study's findings be provided in a repository or as Supporting Information. For data residing with a third party, authors are required to provide instructions with contact information for obtaining the data. PLOS journals do not allow statements supported by "data not shown" or "unpublished results." For such statements, authors must provide supporting data or cite public sources that include it.

To enhance the reproducibility of your results, we recommend that you deposit your laboratory protocols in protocols.io, where a protocol can be assigned its own identifier (DOI) such that it can be cited independently in the future. Additionally, PLOS ONE offers an option to publish peer-reviewed clinical study protocols. Read more information on sharing protocols at https://plos.org/protocols?utm_medium=editorial-email&utm_source=authorletters&utm_campaign=protocols

Please review your reference list to ensure that it is complete and correct. If you have cited papers that have been retracted, please include the rationale for doing so in the manuscript text, or remove these references and replace them with relevant current references. Any changes to the reference list should be mentioned in the rebuttal letter that accompanies your revised manuscript.

Please note, when your manuscript is accepted, an uncorrected proof of your manuscript will be published online ahead of the final version, unless you've already opted out via the online submission form. If, for any reason, you do not want an earlier version of your manuscript published online or are unsure if you have already indicated as such, please let the journal staff know immediately at plosmedicine@plos.org.

If you have any questions in the meantime, please contact me or the journal staff on plosmedicine@plos.org.

We look forward to receiving the revised manuscript by Aug 13 2026 11:59PM.

Sincerely,

Katarzyna Starowicz, PhD

Senior Editor

PLOS Medicine

plosmedicine.org

------------------------------------------------------------

Requests from Editors:

GENERAL EDITORIAL REQUESTS

* Please confirm that your title complies with PLOS Medicine's style. Your title must be nondeclarative and not a question. It should begin with main concept if possible. "Effect of" should be used only if causality can be inferred, i.e., for an RCT. Please place the study design ("A randomized controlled trial," "A retrospective study," "A modelling study," etc.) in the subtitle (ie, after a colon).

* Please confirm that your abstract complies with our requirements, including format (three sections: Background, Methods and Findings, and Conclusions) and providing all the information relevant to this study type https://journals.plos.org/plosmedicine/s/submission-guidelines#loc-abstract

* Please ensure that the Introduction ends with a clear description of the study question or hypothesis.

* Please ensure that all abbreviations are defined at first use throughout the text.

* Please confirm that all numbers presented in the abstract are present and identical to numbers presented in the main manuscript text.

GENERAL

* Please include a statement regarding use or non-use of AI tools and technologies at the end of the Methods. Please specify the name(s) and version(s) of any tool(s) used and a description of their use, including how the AI-assisted or generated content was evaluated by authors, and a clear statement of which aspects of the study, article contents, data, or supporting files were affected or generated by AI tool usage. Please also include a statement specifying who retains responsibility for the final manuscript. See our guidelines for more information: https://journals.plos.org/plosmedicine/s/ethical-publishing-practice#loc-artificial-intelligence-tools-and-technologies.

* Please review your text for claims of novelty or primacy (e.g. 'for the first time') and remove this language. In addition, please check that any use of statistical terms (such as trend or significant) are supported by the data, and if not please remove them.

* Statistical reporting: Please revise throughout the manuscript, including tables and figures.

- Please report statistical information as follows to improve clarity for the reader ""22% (95% CI [13,28]; p</=)"".

- Please separate upper and lower bounds with commas instead of hyphens as the latter can be confused with reporting of negative values.

- Please repeat statistical definitions (HR, CI etc.) for each set of parentheses."

* In the last sentence of the Abstract Methods and Findings section, please describe the main limitation(s) of the study's methodology.

* In the author summary, please revise formatting and ensure you use bullet points.

* In the author summary, in the final bullet point of 'What Do These Findings Mean?', please include the main limitations of the study in non-technical language.

Please see our author guidelines for more information: https://journals.plos.org/plosmedicine/s/revising-your-manuscript#loc-author-summary."

FUNDING STATEMENT

* The funding statement should include: specific grant numbers, initials of authors who received each award, URLs to sponsors’ websites. Also, please state whether any sponsors or funders (other than the named authors) played any role in study design, data collection and analysis, the decision to publish, or preparation of the manuscript. If they had no role in the research, include this sentence: “The funders had no role in study design, data collection and analysis, decision to publish, or preparation of the manuscript.”

* It appears that one or more study authors is affiliated with one or more of the agencies that funded the study. Thus, the statement “The funders had no role in study design, data collection and analysis, decision to publish, or preparation of the manuscript” does not apply. Please revise the Financial Disclosure accordingly, as in "[Author name] is [author's role] at [funding agency]. The funders had no other role in study design…..”

COMPETING INTERESTS STATEMENT

* All authors must declare their relevant competing interests per the PLOS policy, which can be seen here: https://journals.plos.org/plosmedicine/s/competing-interests For authors with ties to industry, please indicate whether any of the interests has a financial stake in the results of the current study.

* Please amend your competing interests statement. Funding information should go to the relevant section and include URLs.

DATA AVAILABILITY

* PLOS defines the “minimal data set” to consist of the data set used to reach the conclusions drawn in the manuscript with related metadata and methods, and any additional data required to replicate the reported study findings in their entirety. Authors do not need to submit their entire data set, or the raw data collected during an investigation. Please submit the following data:

The values behind the means, standard deviations and other measures reported;

The values used to build graphs;

The points extracted from images for analysis.

* For studies in which a novel model is central to the manuscript's findings, as is the case here, authors are responsible for providing the source code needed to replicate the study's findings in a repository (such as GitHub, SourceForge or Bitbucket) or a cloud computing service (such as Code Ocean). Please include a citable digital object identifier (DOI) or other persistent identifier of the code (such as through linkage with Zenodo). Protection of authors’ intellectual property will not be cause for exception. Please explain in the manuscript’s Data Availability Statement how readers can access the shared code.

* Thank you for agreeing to make your data available. At this time, please provide the link to the data repository and accession numbers required for access.

* Please include the statement on code availability in the data availability statement. Please see guidance here:https://journals.plos.org/plosmedicine/s/code-sharing-guidance#loc-data-availability-statement-examples

ETHICS AND CONSENT

* Please revise for use of patient-centered language. Please note that patient-centered language is constructed with the use of post-modified nouns (e.g. 'patients with psoriasis’ (or similar) instead of ‘psoriasis patients’) putting the person first in the sentence structure.

FIGURES

* When a p value is given, please specify the statistical test used to determine it in the legend

* Where data points are discrete, please ensure that they are depicted in the figures as discrete data and not as a continuous line.

* Please provide the unadjusted comparisons as well as the adjusted comparisons in all relevant Tables

* Please specify the variables controlled for in all relevant Tables

* Please consider if moving some of the figures shared in the supplementary information into the main text would aid the reader. I am referring to Sup F1 and F2 to be Figure 1 and 2 as this will give potential reader good overview of your study from the beginning. When you do that please revise if remaining reference are correct in whole manuscript.

ACKNOWLEDGMENT

* Please include an acknowledgment of study participants.

OBSERVATIONAL, COHORT, CROSS-SECTIONAL, AND CASE CONTROL STUDIES

* Your study is observational and therefore causality cannot be inferred. Please remove language that implies causality and refer to associations instead.

* For all observational studies, in the manuscript text, please indicate: (1) the specific hypotheses you intended to test, (2) the analytical methods by which you planned to test them, (3) the analyses you actually performed, and (4) when reported analyses differ from those that were planned, transparent explanations for differences that affect the reliability of the study's results. If a reported analysis was performed based on an interesting but unanticipated pattern in the data, please be clear that the analysis was data-driven.

CHECKLISTS

* Thank you for providing TARGET and STROBE Checklists in Supporting information files. Please extract them from this one file and upload as separate files together with revised manuscript.

OTHER

* Study Design and Data Source section – Line 144 and 148 repetitions of TARGET guideline following. Revise from Line 143 to “This study is reported in accordance with the STROBE reporting guideline [16] (S2 Checklist)and TARGET guideline for emulating a target trial [17] (S1 Checklist). Remove line 148 and revise the Checklists tables references – probably need to swap order so it is chronological.

* I am wondering if inclusion and exclusion criteria, and outcomes are not better suited in main body of the manuscript rather than in Supplementary text. Please consider moving it.

--------------------------------

Comments from Reviewers:

Reviewer #1: Thank you for the detailed response to my comments. The authors have adequately addressed all of my previous comments. I have no further comments or revisions to suggest.

Reviewer #2: I am satisfied with the revision.

Reviewer #3: The authors have addressed all my concerns sufficiently

Any attachments provided with reviews can be seen via the following link:

[LINK]

# Author comment: Author response to Decision Letter 3

**DOI:** 10.1371/journal.pmed.1005262.r004

12 Aug 2026

Attachment Submitted filename: Response_to_Editors_v6.docx

# Editor report: Decision Letter 3

**DOI:** 10.1371/journal.pmed.1005262.r005

**Authors:** Katarzyna Starowicz

21 Aug 2026

Dear Dr. Lee,

Thank you very much for re-submitting your manuscript "Serotonin transporter inhibition and immune checkpoint blockade outcomes: a target trial emulation with pan-cancer transcriptomic validation" (PMEDICINE-D-26-00932R3) for review by PLOS Medicine.

As mentioned in my previous decision email we are planning to accept your manuscript for the publication but there are some details in a text which require clarification before publication.

The remaining issues that need to be addressed are listed at the end of this email.

***Please note while forming your response, if your article is accepted, you may have the opportunity to make the peer review history publicly available. The record will include editor decision letters (with reviews) and your responses to reviewer comments. If eligible, we will contact you to opt in or out.***

In revising the manuscript for further consideration here, please ensure you address the specific points made by each reviewer and the editors. In your rebuttal letter you should indicate your response to the reviewers' and editors' comments and the changes you have made in the manuscript. Please submit a clean version of the paper as the main article file. A version with changes marked must also be uploaded as a marked up manuscript file.

Please also check the guidelines for revised papers at http://journals.plos.org/plosmedicine/s/revising-your-manuscript for any that apply to your paper. If you haven't already, we ask that you provide a short, non-technical Author Summary of your research to make findings accessible to a wide audience that includes both scientists and non-scientists. The Author Summary should immediately follow the Abstract in your revised manuscript. This text is subject to editorial change and should be distinct from the scientific abstract.

As this are only text changes we expect to receive your revised manuscript by Wednesday 26th August. Please email us (plosmedicine@plos.org) if you have any questions or concerns.

Please note, when your manuscript is accepted, an uncorrected proof of your manuscript will be published online ahead of the final version, unless you've already opted out via the online submission form. If, for any reason, you do not want an earlier version of your manuscript published online or are unsure if you have already indicated as such, please let the journal staff know immediately at plosmedicine@plos.org.

If you have any questions in the meantime, please contact me or the journal staff on plosmedicine@plos.org.

We look forward to receiving the revised manuscript by Aug 26 2026 11:59PM.

Sincerely,

Katarzyna Starowicz, PhD

Associate Editor

PLOS Medicine

plosmedicine.org

------------------------------------------------------------

Requests from Editors:

•Please revise the title to: Selective serotonin reuptake inhibitors and immune checkpoint blockade in solid tumors: A target trial emulation with pan-cancer transcriptomic analyses [or analysis, singular]

•line 33 of Abstract, instead of "We compared..." you need to add "Using a target trial emulation framework, we compared..." [or "In a target trial emulation, we compared..."

•line 35: if this statement "and examined SLC6A4 expression and immune suppression...." is not from the same patients as the TTE, then they need to so state. e.g. "and examined SLC6A4 expression and immune suppression across cancers in The Cancer Genome Atlas."

•also--it's not immune suppression if you are just looking at elevated PD-L1, for e.g.

•and did patients really receive the antidepressant for the first time on the first day of ICI infusion? an active prescription for an SSRI or a benzodiazepine overlapping the index date or starting within the preceding 90 days; (line 200)

•Not sure if “transcriptomic validation” term is correct here. You are looking more at association.

•line 47: 2-year mortality--all cause or cancer specific. You should state that.

•lines 51, 52--you need to say in which groups these outcomes were less frequent and not leave it open to interpretation.

•"3 negative control outcomes" is not very informative

•line 57:"performance status is not recorded in the network," what does this mean? clarify for general reader

•also you should add a comment on the limitations of the TCGA data.

•Line 159: all cause or cancer specific?

•Line 161 not immune suppression

•Line 304 not validation

•Line 306 are that 8272 samples from 8272 patients

•Line 323 Us of AI tools is separate section

•Line 385: the sentence “ Findings were consistent across the analyses requested during revision” does not add much as reader doesn’t know what is from original and what from revised MS

•Line 399 : Not sure if this heading makes sense, I would rather say with “Immune response”

•Line 580 DOI URL is not working, please correct

# Author comment: Author response to Decision Letter 4

**DOI:** 10.1371/journal.pmed.1005262.r006

21 Aug 2026

Attachment Submitted filename: Response_to_Editors_v7.docx

# Editor report: Decision Letter 4

**DOI:** 10.1371/journal.pmed.1005262.r007

**Authors:** Katarzyna Starowicz

27 Aug 2026

Dear Dr Lee,

On behalf of my colleagues and the Academic Editor, Tessho Maruyama, I am pleased to inform you that we have agreed to publish your manuscript "Selective serotonin reuptake inhibitors and immune checkpoint blockade in solid tumors: A target trial emulation with pan-cancer transcriptomic analyses" (PMEDICINE-D-26-00932R4) in PLOS Medicine.

Before your manuscript can be formally accepted you will need to complete some formatting changes, which you will receive in a follow up email. Please be aware that it may take several days for you to receive this email; during this time no action is required by you. Once you have received these formatting requests, please note that your manuscript will not be scheduled for publication until you have made the required changes.

In the meantime, please log into Editorial Manager at http://www.editorialmanager.com/pmedicine/, click the "Update My Information" link at the top of the page, and update your user information to ensure an efficient production process.

PRESS

We frequently collaborate with press offices. If your institution or institutions have a press office, please notify them about your upcoming paper at this point, to enable them to help maximise its impact. If the press office is planning to promote your findings, we would be grateful if they could coordinate with medicinepress@plos.org. If you have not yet opted out of the early version process, we ask that you notify us immediately of any press plans so that we may do so on your behalf.

We also ask that you take this opportunity to read our Embargo Policy regarding the discussion, promotion and media coverage of work that is yet to be published by PLOS. As your manuscript is not yet published, it is bound by the conditions of our Embargo Policy. Please be aware that this policy is in place both to ensure that any press coverage of your article is fully substantiated and to provide a direct link between such coverage and the published work. For full details of our Embargo Policy, please visit http://www.plos.org/about/media-inquiries/embargo-policy/.

To enhance the reproducibility of your results, we recommend that you deposit your laboratory protocols in protocols.io, where a protocol can be assigned its own identifier (DOI) such that it can be cited independently in the future. Additionally, PLOS ONE offers an option to publish peer-reviewed clinical study protocols. Read more information on sharing protocols at https://plos.org/protocols?utm_medium=editorial-email&utm_source=authorletters&utm_campaign=protocols

Thank you again for submitting to PLOS Medicine. We look forward to publishing your paper.

Sincerely,

Katarzyna Starowicz, PhD

Associate Editor

PLOS Medicine
